# Comparative effects of different intensities of aerobic and resistance exercise on glycemic control and cardiorespiratory fitness in middle-aged older patients with type 2 diabetes: a network meta-analysis

**DOI:** 10.3389/fpubh.2026.1818686

**Published:** 2026-05-25

**Authors:** Jiacheng Yu, Xinchun Li, Hao Yu, Yijun Huang

**Affiliations:** School of Physical Education, Ludong University, Yantai, China

**Keywords:** aerobic exercise, cardiorespiratory fitness, type 2 diabetes mellitus, network meta-analysis, older adults, resistance training

## Abstract

**Introduction:**

Type 2 diabetes mellitus (T2DM) is highly prevalent among middle-aged and older adults and is associated with adverse metabolic, cardiovascular, and functional outcomes. Although aerobic exercise, resistance training, and combined training are commonly recommended, comparative evidence regarding the efficacy and safety of different exercise intensities remains limited. Therefore, we conducted a systematic review and network meta-analysis to compare the effects of different aerobic and resistance exercise modalities and intensities in this population.

**Methods:**

PubMed, Embase, Cochrane Library, and Web of Science were systematically searched from inception to May 5, 2026, without language restrictions. A combined strategy of controlled vocabulary and free-text terms was applied. The primary outcomes were glycated hemoglobin (HbA1c), fasting plasma glucose (FPG), peak oxygen uptake (VO₂peak), systolic blood pressure (SBP), and resting heart rate (HR). Risk of bias was assessed using the Cochrane Risk of Bias 2 tool, and the certainty of evidence was evaluated with the CINeMA framework. The protocol was registered in PROSPERO (CRD420251270452).

**Results:**

Twenty-nine randomized controlled trials comprising 1,301 participants and 10 exercise interventions were included. For HbA1c, moderate certainty evidence indicated that high-intensity resistance training (HIRT; MD − 0.62, 95% CI − 0.93 to −0.30), low-certainty evidence indicated that moderate-intensity aerobic training (MIAT; MD − 0.58, 95% CI − 1.10 to −0.05), high-intensity aerobic training combined with moderate-intensity resistance training (HIAT–MIRT; MD − 0.54, 95% CI − 1.02 to −0.06), and low-intensity resistance training (LIRT; MD − 0.54, 95% CI − 1.00 to −0.09) significantly reduced HbA1c compared with usual care (UC), with HIRT ranking highest (SUCRA 78.6%). For FPG, moderate certainty evidence indicated that only moderate-intensity resistance training (MIRT; MD − 29.13 mg/dL, 95% CI − 57.68 to −0.58) showed a statistically significant reduction versus UC and ranked first (SUCRA 80.5%). For VO₂peak, high certainty evidence indicated that HIAT–HIRT (MD 3.75 mL/kg/min, 95% CI 1.11 to 6.38), HIAT (MD 3.14 mL/kg/min, 95% CI 1.33 to 4.95), and moderate certainty evidence indicated that HIAT–MIRT (MD 1.80 mL/kg/min, 95% CI 0.11 to 3.48) were associated with significant improvements compared with UC. For SBP, low certainty evidence indicated that HIRT demonstrated a significant reduction (MD − 3.68 mmHg, 95% CI − 7.20 to −0.16). No intervention significantly reduced resting HR relative to usual care. Global inconsistency testing did not indicate major network-wide incoherence; however, loop-specific analysis suggested potential inconsistency in the FPG network. Sensitivity analyses generally supported the stability of the main findings when network connectivity was preserved, although sparse networks, particularly for VO₂peak and resting HR, warrant cautious interpretation.

**Conclusion:**

Among middle-aged and older adults with T2DM, exercise interventions demonstrate outcome-specific effects across metabolic control, cardiorespiratory fitness, and cardiovascular parameters. Moderate-certainty evidence indicated that HIRT was associated with clinically meaningful reductions in HbA1c, whereas low-certainty evidence suggested similar associations for MIAT, HIAT–MIRT, and LIRT. Moderate certainty evidence indicated that MIRT showed may be associated with a potential advantage for improving FPG. For VO₂peak, HIAT–HIRT and HIAT were associated with the largest improvements compared with usual care, but these findings were based on a sparse evidence network and should be considered exploratory. However, because only a limited number of studies were included for this outcome and the evidence network was sparse, these results should be interpreted with caution. For blood pressure control, low-certainty evidence suggested that HIRT may be the only modality associated with a significant reduction in SBP. Although HIAT showed potential benefits for resting HR, the current evidence remains inconclusive. However, the certainty of evidence for most comparisons involving FPG and SBP was rated as low or very low; therefore, these findings should be interpreted cautiously. To bolster the credibility of the evidence and guide tailored exercise recommendations for this group, we require extensive, meticulously planned randomized studies featuring uniform outcome measures, extended follow-up periods, and direct comparisons among primary exercise types.

**Systematic review registration:**

https://www.crd.york.ac.uk/PROSPERO/view/CRD420261306289.

## 1 Introduction

Adult-onset diabetes, known medically as type 2 diabetes mellitus, represents a complicated metabolic condition where the body either does not produce enough insulin or does not use it effectively, throwing carbohydrate, fat, and protein processing out of whack throughout the system (1). T2DM is highly prevalent among middle-aged and older adults and is closely associated with progressive declines in physical function (2). In this population, the high burden of T2DM largely reflects age-related physiological deterioration and adverse changes in body composition, both of which exacerbate pancreatic *β*-cell dysfunction and insulin resistance (3–5). These metabolic and functional derangements contribute to skeletal muscle atrophy, a process that is further amplified by the presence of T2DM itself (6). Consequently, T2DM poses a substantial threat to healthy aging, increasing the risk of functional impairment, injurious falls, and reduced quality of life (7, 8).

In individuals with T2DM, cardiorespiratory fitness is widely recognized as a central determinant of metabolic control, cardiovascular risk, functional capacity, and long-term prognosis, and it represents a key therapeutic target of exercise-based interventions. A range of non-pharmacological strategies have been proposed for the prevention and management of T2DM in middle-aged and older adults, including nutritional counseling, structured lifestyle modification, and moderate-intensity aerobic exercise. Given the combined neuromuscular consequences of aging and T2DM, resistance training has emerged as another cornerstone intervention, as improvements in muscle strength, mass, and function translate directly into enhanced functional performance (9–12). Conventional resistance training, typically performed at 60–80% of one-repetition maximum (1RM), has been shown to increase skeletal muscle mass and strength while contributing to improved glycemic control (13). In addition to its metabolic effects, resistance training may reduce systemic inflammation by lowering circulating inflammatory biomarkers. This is especially important since persistent inflammation has been linked to disrupted insulin response and an increased likelihood of heart problems (14–16). Aerobic exercise, in contrast, has demonstrated robust benefits for cardiovascular health, mitigation of brain atrophy, and preservation of white matter volume (17, 18). Through its effects on metabolic regulation and adaptive remodeling of the cardiovascular and neuromuscular systems, aerobic training remains one of the most established and widely implemented exercise modalities for improving cardiorespiratory fitness (19). Importantly, combined aerobic and resistance training appears to confer additive—or even synergistic—benefits in patients with T2DM. Beyond glycemic control, this multimodal approach enhances muscle strength, cardiorespiratory capacity, and exercise tolerance, thereby contributing to overall functional improvement and potentially reducing cardiovascular risk (20).

While traditional research methods like randomized controlled trials and standard meta-analyses point to resistance and aerobic exercises potentially improving body composition and physical function, there’s a notable gap when it comes to direct comparisons between various exercise types and intensity levels. Most studies pit a single exercise regimen against standard care or generic lifestyle recommendations, resulting in limited evidence for direct head-to-head comparisons. Network meta-analysis provides a statistical framework that can seamlessly blend both direct and indirect evidence, allowing us to rank interventions by their relative effectiveness and probability of success. Therefore, this study used network meta-analysis to evaluate how different exercise intensities and modalities impact HbA1c, fasting plasma glucose, VO₂peak, systolic blood pressure, and heart rate in middle-aged and older adults with type 2 diabetes. The aim was to provide comparative evidence to inform individualized exercise prescription and rehabilitation strategies for middle-aged and older adults with T2DM.

## 2 Materials and methods

The NMA was meticulously executed and documented, adhering strictly to the guidelines outlined in the PRISMA extension specifically for network meta-analyses (PRISMA-NMA; see Appendix S1). To promote clarity and ensure the replicability of our methodology, we proactively registered our study protocol in the PROSPERO registry (CRD420251270452).

### 2.1 Data sources and search strategy

We systematically searched PubMed, Embase, the Cochrane Library, and Web of Science, up until May 5, 2026. Our search was language agnostic. We used a combination of specific subject terms, like MeSH and Emtree, alongside more general keywords. Our key search terms were “Type 2 Diabetes Mellitus,” “Adult-Onset Diabetes,” “Non-Insulin-Dependent Diabetes Mellitus,” “Aged,” “Older Adults” “Resistance Training,” “Aerobic Exercise,” “Exercise,” and “Randomized Controlled Trial.” The full search strategy is provided in Supplementary Table S2.

### 2.2 Selection criteria

Inclusion Criteria:

1. Randomized controlled trials enrolling middle-aged and older adults with type 2 diabetes mellitus. Although conventional definitions classify older adults as ≥60 or ≥65 years, a substantial proportion of trials in this field report mean participant ages between 55 and 58 years. To ensure adequate evidence synthesis while maintaining clinical relevance, we defined eligible populations as those with a mean age ≥55 years. Although 60 or 65 years is often used in geriatric research, the use of 55 years as a lower threshold for later-life/older-adult populations has also been adopted in published evidence syntheses (21). T2DM was defined according to established diagnostic criteria: glycated hemoglobin (HbA1c) ≥ 6.5% and/or fasting plasma glucose (FPG) ≥ 126 mg/dL (7.0 mmol/L) (22).
2. Eligible interventions included structured exercise programs categorized by modality and intensity: Moderate-intensity aerobic training (MIAT); High-intensity aerobic training (HIAT); Low-intensity resistance training (LIRT); Moderate-intensity resistance training (MIRT); High-intensity resistance training (HIRT); HIAT combined with HIRT (HIAT-HIRT); HIAT combined with MIRT (HIAT-MIRT); HIAT combined with LIRT (HIAT-LIRT). Resistance training intensity was classified according to the American College of Sports Medicine (ACSM) guidelines: Low intensity was defined as <50% of one-repetition maximum (1RM) or >12 repetition maximum (RM), including ≥13–15RM; Moderate intensity: 50–69% of one-repetition maximum (1RM) or 8–12 repetition maximum (RM)High intensity: ≥70% 1RM or ≤6–8 RM. Aerobic training intensity was defined based on ACSM criteria as: Moderate intensity: 40–59% heart rate reserve (HRR) or oxygen uptake reserve (VO₂R), 64–76% of maximal heart rate (HRmax), or rating of perceived exertion (RPE) 12–13. High intensity: ≥60% HRR or VO₂R, ≥77% HRmax, or RPE ≥ 14 (23) If a resistance exercise program had overlapping or progressively increasing intensity levels with a clearly described progression plan to achieve a higher target intensity—for example, progression from 50% to ≥70% 1RM, or from 12RM to ≤6–8RM across the intervention period—it was classified as progressive-intensity resistance training and grouped according to the highest target intensity achieved. If an exercise program had overlapping intensity levels with a clear progression plan to achieve a given intensity (e.g., 12 weeks of 40–80%HRR, with 10% increase in %HRR every 4 weeks), it was grouped as the highest target intensity. To enhance transparency and reproducibility of node classification, a trial-arm-level classification table was added to the Supplementary material. Supplementary Table S60 presents, for each included trial arm, the original intervention description, the reported intensity metric, the rule used for classification, and the final assigned network node. Control conditions included usual care, health education, standard dietary advice, non-specific interventions defined by investigators, or active exercise control protocols not meeting predefined intensity criteria. Active control was defined as a comparator condition involving participant contact, supervision, education, sham exercise, stretching, flexibility/toning activities, or low-intensity physical activity that did not satisfy the prespecified criteria for structured aerobic or resistance exercise training. These conditions were grouped as an active control node because they were intended to control for attention, contact time, expectation, or nonspecific activity effects, rather than to deliver a target aerobic or resistance training dose. This approach is consistent with methodological literature on control condition design in exercise trials, where active controls are distinguished from inactive controls such as no treatment, usual care, or wait-list controls (24).
3. Studies were required to report at least one of the following outcomes: HbA1c: Glycated hemoglobin formed through non-enzymatic glycation of hemoglobin, reflecting average glycemic exposure over approximately 2–3 months. VO₂peak: The highest rate of oxygen uptake attained during incremental exercise testing, typically measured via cardiopulmonary exercise testing, representing cardiorespiratory fitness Systolic blood pressure (SBP): The peak arterial pressure during ventricular systole, reflecting the hemodynamic load imposed on arterial walls (25) FPG: Plasma glucose concentration measured after at least 8 h of fasting, widely used as an index of glycemic control (26) Resting heart rate (HR): The number of heartbeats per minute at rest, reflecting autonomic regulation of cardiac function (27)

Exclusion Criteria:

1. Multiple publications reported data from the same cohort; in such cases, only the most comprehensive and most recent report was retained.
2. Target outcome data were unavailable, non-extractable, or non-convertible (e.g., missing means, standard deviations, change scores), and could not be obtained from study authors.
3. The study design was observational (including cohort, case–control, or cross-sectional studies), case series, case reports, narrative reviews, or conference abstracts lacking full data.

### 2.3 Data extraction

Two investigators independently screened the randomized controlled trials and extracted relevant data in line with the PRISMA guidelines. They meticulously double-checked each data entry, and any discrepancies were resolved through discussion, with arbitration by a third reviewer when necessary, or resolved by a third-party mediator if need be. They meticulously extracted the basics for every trial, including the lead author’s name, the publication date, the mean age of the participants, their geographic locale, how long the follow-up period was, what the intervention was all about, its counterpart, and what results were measured.

When it came to measuring continuous outcomes—like HbA1c, FPG, VO₂peak, SBP, and resting HR—they zeroed in on the average change from the starting point, as well as the standard deviation. If a study only offered baseline and follow-up means and SDs, they computed the mean change by taking the difference between these two points. The standard deviation of the change score was calculated from the baseline and follow-up standard deviations using an assumed correlation coefficient from the baseline and follow-up SDs, and a fancy correlation coefficient (R), using a standard formula. Sensitivity analyses were conducted using alternative correlation coefficients to assess the robustness of results to this assumption. In this study, the default *R* value was set at 0.5, and sensitivity analyses were further conducted using *R* = 0.25 and *R* = 0.75 (28). For multi-arm trials, pairwise comparisons were generated using the augment approach, while preserving the within-study correlation structure during estimation to avoid underestimating standard errors through repeated use of the same control group.


MEANchange=Endpoint Mean−Baseline Mean

$$\text{SDchange}=\sqrt{\text{Baseline}\;{\mathrm{SD}}^{2}+\text{Endpoint}\;{\mathrm{SD}}^{2}-2R\cdot \text{Baseline}\;\mathrm{SD}\cdot \text{Endpoint}\;\mathrm{SD}}$$

### 2.4 Quality assessment

We assessed the potential for bias in each randomized controlled trial included in our analysis using the Cochrane Risk of Bias 2.0 (RoB 2) assessment tool. This methodology evaluates bias across five key areas: (1) the randomization process; (2) any deviations from the planned interventions; (3) issues with missing outcome data; (4) how the outcomes were measured; and (5) the selection of which results were reported. Each domain, along with the overall risk of bias at the study level, was categorized as either “low risk,” “some concerns,” or “high risk” following the specific guidelines provided by RoB 2.

### 2.5 Assessment of transitivity and potential effect modifiers

The transitivity assumption was assessed *a priori* by examining whether trials contributing to different treatment comparisons were sufficiently comparable with respect to key clinical and methodological characteristics that could modify the relative effects of exercise interventions. Potential effect modifiers were selected based on clinical relevance and prior evidence in exercise interventions for type 2 diabetes, including mean age, baseline glycemic status, diabetes duration, medication background, intervention duration, exercise frequency, session duration, supervision level, dietary or nutritional co-interventions, and comparator condition. These variables were extracted at the study or trial-arm level where available and compared descriptively across treatment nodes and direct comparisons. Statistical inconsistency tests were interpreted as assessments of coherence between direct and indirect evidence, but the plausibility of transitivity was judged primarily according to the distribution of these potential effect modifiers across the network.

To further explore potential study-level effect modification, univariable network meta-regression analyses were performed for covariates with sufficient reporting across trials, including geographical region, follow-up duration, mean age, baseline HbA1c, session duration, and weekly training frequency. Variables that were inconsistently reported, such as medication background, diabetes duration, dietary co-interventions, and supervision level, were assessed narratively rather than quantitatively.

### 2.6 Statistical analysis

We executed all network meta-analyses with Stata 17.0 MP (Stata Corp, College Station, TX, USA). For continuous outcomes that shared the same measurement scale and units across studies, we estimated pooled effects as mean differences (MDs) complete with 95% confidence intervals (CIs). When researchers employed different scales or units for measuring the same outcome, we calculated standardized mean differences (SMDs) alongside 95% CIs. Our primary analyses operated under the consistency assumption employing a random-effects model. Between-study variance (τ^2^) was derived through restricted maximum likelihood (REML) estimation. To assess global inconsistency in networks featuring closed loops, we utilized the design-by-treatment interaction model. Local inconsistency underwent scrutiny via node-splitting methodology, with two-sided *p*-values below 0.10 suggesting possible inconsistency. Additionally, we quantified loop-specific inconsistency using inconsistency factors (IFs); a 95% CI encompassing zero signaled no statistically discernible divergence between direct and indirect evidence. We visually represented network geometry, where node size corresponded to the total sample size for each intervention, and edge thickness indicated the number of studies supporting each direct comparison. To establish rankings among competing interventions, we computed the surface under the cumulative ranking curve (SUCRA), the probability of being the best treatment (PrBest), and mean rank values, thereby offering complementary metrics to bolster interpretability and robustness. When at least 10 studies were available for a particular outcome, we probed publication bias and small-study effects using comparison-adjusted funnel plots. The stability of our pooled estimates underwent rigorous examination through leave-one-out sensitivity analyses. This approach involved systematically excluding each study and re-estimating the random-effects consistency model to verify the stability of both effect direction and magnitude. To investigate potential effect modification at the study level, we conducted univariable network meta-regression analyses. We reported regression coefficients, 95% CIs, and Wald test *p*-values, with statistical significance established at *p* < 0.05.

### 2.7 GRADE assessment

The assessment of evidence certainty for our network estimates was carried out with the GRADE framework, bolstered by the CINeMA (Confidence in Network Meta-Analysis) method. Given that all studies involved were randomized controlled trials, we began with a high level of certainty. We examined certainty across six key areas: study bias, the level of indirectness, precision, heterogeneity, inconsistency between direct and indirect evidence, and reporting bias. To gauge within-study bias, we employed RoB 2 at the domain level, merging the findings using the CINeMA’s contribution matrix to reflect each study’s impact on our network estimates. The concept of indirectness was evaluated based on the assumption of transitivity, taking into account factors like disease severity at baseline, intervention strength, and duration of follow-up. Imprecision was measured against a predefined minimum important difference (MID). For clinical interpretation and assessment of imprecision, minimally important thresholds were prespecified for each continuous outcome: 0.5 percentage points for HbA1c, 10 mg/dL for fasting plasma glucose, 3.5 mL/kg/min for VO₂peak, 5 mmHg for systolic blood pressure, and 5 beats/min for resting heart rate. The HbA1c threshold was selected because a 0.5 percentage-point difference is commonly regarded as clinically meaningful in diabetes care (29). The VO₂peak threshold corresponds to 1 metabolic equivalent, a widely used clinically meaningful difference in cardiorespiratory fitness research (30). The SBP threshold was based on randomized evidence showing that a 5 mmHg reduction in systolic blood pressure is associated with a meaningful reduction in major cardiovascular events (31). Because no universally accepted MCID exists for FPG or resting HR, pragmatic thresholds of 10 mg/dL and 5 beats/min were selected based on clinical interpretability and established glycemic or prognostic associations (32, 33). Heterogeneity was analyzed using the (τ^2^) from the random-effects model and the position of the prediction interval in relation to the MID. For incoherence, we looked at networks with closed loops, using node-splitting (SIDE) or the design-by-treatment interaction model to juxtapose direct and indirect estimates. We tackled between-study bias by examining trial registration details, searching through grey literature, and scrutinizing funnel plots for small-study effects. Each domain received a rating from “no concerns” to “major concerns,” which aligned with the GRADE criteria and resulted in downgrades of 0, 1, or 2 levels, respectively. The overall certainty of the evidence was then categorized as high, moderate, low, or very low.

## 3 Results

### 3.1 Systematic review and characteristics of the included studies

**Figure 1:**
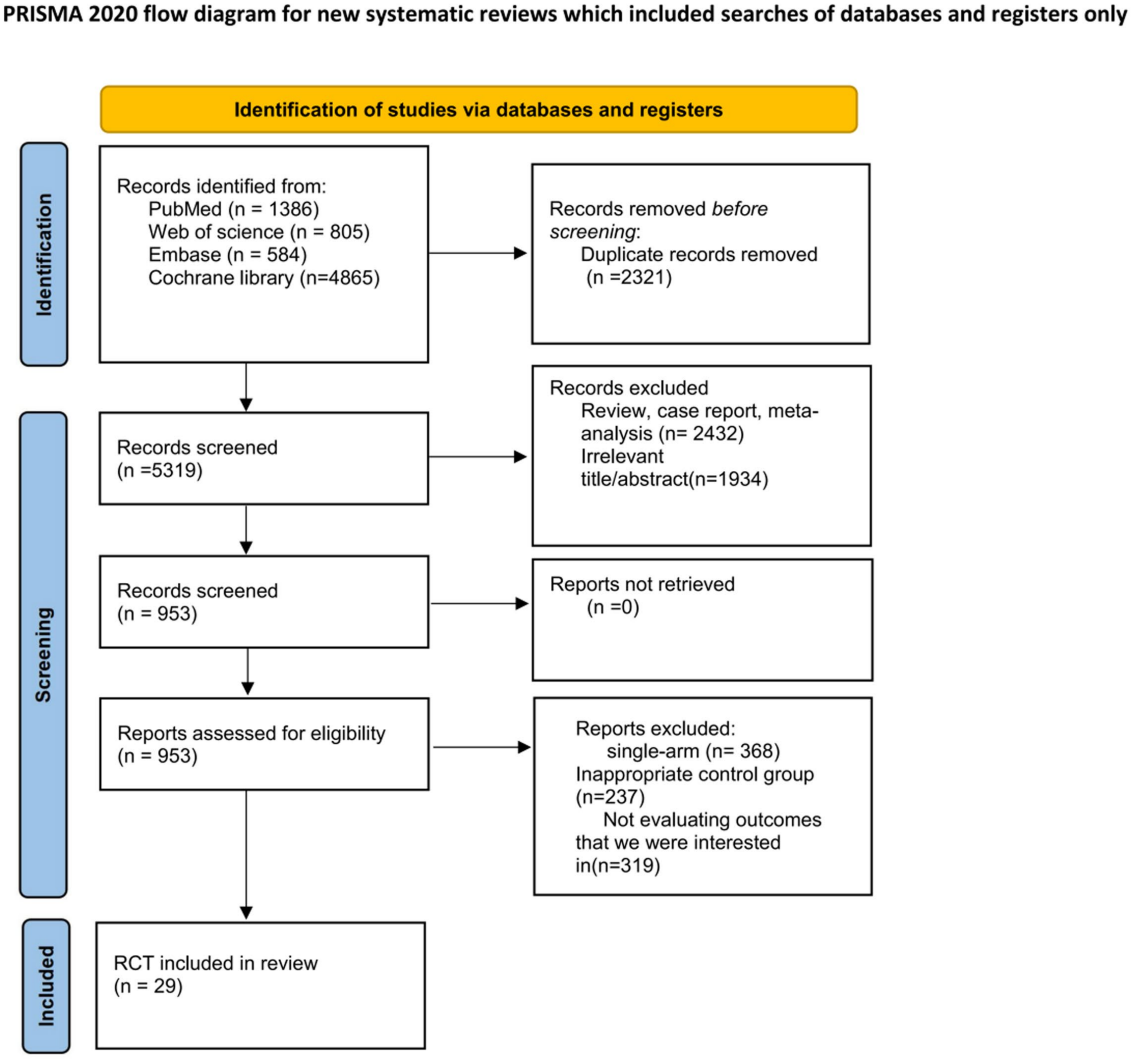
PRISMA flow diagram of study selection.

A total of 7,640 records were initially identified. After removal of duplicates and screening of titles and abstracts, 5,319 articles were assessed in full text. Out of the lot, 29 randomized controlled trials were deemed eligible and made the cut for the meta-analysis (see Figure 1). The studies included 1,301 participants and examined eight different exercise regimens, categorized by type and intensity: low, moderate, and high-intensity resistance training, as well as moderate and high-intensity aerobic training. Additionally, we looked at two control groups: active and standard care. The participants’ average age spanned from 55 to 72.9 years. The trials were conducted across 14 countries, with Australia contributing the largest number of studies. The follow-up periods ranged from 1.5 to 12 months. A comprehensive breakdown of all the studies is provided in Table 1.

**Table 1:** Baseline characteristics of the included studies.

**First author**	**Year**	**Mean age**	**Country**	**Follow-up**	**Session duration (min)**	**Frequency (weekly)**	**Experimental group (*n*)**	**Control group (*n*)**	**Experimental intervention**	**Control intervention**	**Outcomes**
Cíntia E Botton (46)	2018	69.7	Brazil	3 M	45	3	13	13	Conventional resistance training plus functional training, 3 sessions/week at 12–15 RM.	Once weekly joint-mobility exercises plus static stretching for major muscle groups.	HbA1c
N W Cheung (47)	2009	60.5	Australia	4 M	30	5	20	17	Exercise program 5 days/week, 30 min/session (5-min warm-up; resistance exercise; 30-min relaxation; 5-min cool-down).	No exercise intervention.	HbA1c
Yutaro Yamamoto (48)	2021	72.9	Japan	12 M	15	7	18	17	Home-based elastic-band resistance training, 15 min/day.	No lifestyle intervention.	HbA1c
Yu-Hsuan Chien (49)	2022	67	China	3 M	30	3	19	18	12-week home-based progressive sandbag resistance training, 3 sessions/week (~30 min/session), upper-limb (curls, shoulder press) and lower-limb (hip abduction/adduction, step-ups, heel raises) exercises. Initial load 0.5 kg (RPE ≈ 13); 3 sets of 8–15 repetitions per exercise, progressed to 20 repetitions before increasing load to 1 kg; included 5–10 min warm-up and cool-down.	Usual lifestyle without structured exercise.	HbA1c
Nikolaos P. E. Kadoglou (50)	2012	61.3	Greece	3 M	60	3	23	24	Supervised moderate-intensity resistance training, 3 sessions/week for 3 months; 2–3 sets of 8 machine-based exercises at 60–80% 1RM (6–8 repetitions).	Structured exercise counseling aiming for ≥150 min/week self-reported moderate physical activity; no supervised resistance training.	HbA1c、VO_2_peak、SBP、FPG
Ping-Lun Hsieh (51)	2018	71.2	China	3 M	45	3	15	15	Supervised progressive resistance training, 3 sessions/week for 12 weeks; 8 exercises, 3 sets of 8–12 repetitions, progressing from 40 to 50 to 75% 1RM.	Maintenance of habitual lifestyle.	HbA1c、VO_2_peak、SBP、HR
Anderson Rech (52)	2019	69	Brazil	3 M	45	3	18	21	Supervised progressive resistance training, 3 sessions/week for 12 weeks, combining functional and conventional resistance exercises; 2–3 sets of 10–12 repetitions with load progression based on 15RM testing.	Active control: once-weekly low-intensity joint mobilization and static stretching (~45 min/session), with no aerobic or resistance component.	HbA1c、FPG
George Mavros (53)	2013	69	Australia	12 M	30	3	36	48	Supervised high-intensity progressive resistance (power) training, 3 sessions/week on pneumatic equipment at 80% 1RM; 3 sets of 8 repetitions targeting major muscle groups.	Supervised sham exercise at the same frequency/duration using the same equipment; resistance set as low as possible and not progressed.	HbA1c
Theng Choon Ooi (54)	2021	61.68	Malaysia	4 M	15	5	28	31	16-week high-intensity progressive resistance training using elastic resistance tubing; 12 exercises, 3 sets of 8 repetitions (Borg 16–18) with progressive overload.	Usual care without structured exercise.	HbA1c、HR、SBP、FPG
Xiaojun Ma (55)	2024	66.19	China	6 M	50	3	31	33	Moderate-intensity resistance training at 60–70% 1RM (upper limb, lower limb, and core), 50 min/session including warm-up/cool-down, 3 sessions/week for 6 months, in addition to standard diabetes education.	Diabetes education and lifestyle counseling (diet, exercise advice, medication use, glucose monitoring) without supervised structured resistance training; usual lifestyle maintained.	HbA1c、FPG、SBP
Carmen Castaneda (34)	2002	66	USA	4 M	45	3	31	31	Supervised 16-week high-intensity progressive resistance training, 3 sessions/week (~45 min/session): 5-min warm-up; ~35 min machine-based resistance exercises (chest press, leg press, upper back, knee extension, knee flexion), 3 sets of 8 repetitions, progressing from 60–80% to 70–80% 1RM; 5-min cool-down.	Usual diabetes care with self-management advice.	HbA1c、FPG、SBP、HR
David W Dunstan (56)	2002	67.2	Australia	3 M	55	3	16	13	Supervised high-intensity progressive resistance training, 3 sessions/week: first 2 weeks at 50–60% 1RM, then 75–85% 1RM; 9 upper- and lower-body exercises, 3 sets of 8–10 repetitions, 90–120 s inter-set rest; 5-min warm-up and 5-min cool-down.	Low-intensity control activity, 3 sessions/week (5-min unloaded cycle ergometry plus ~30 min static stretching); no resistance training (reported duration 6 months).	HbA1c、FPG
Kenneth M Madden (57)	2010	71.5	Canada	3 M	60	3	19	20	Supervised moderate-to-high intensity aerobic training, 3 sessions/week for 12 weeks (10-min warm-up; 20-min treadmill; 20-min cycle ergometer; 10-min cool-down stretching); intensity progressed from 50–60% HRmax (weeks 1–2) to 80–85% HRmax.	Supervised low-intensity non-aerobic activity (gentle core stabilization and very light dumbbell exercises), with deliberate avoidance of aerobic exercise.	FPG、SBP、HR
Kenneth M Madden (58)	2013	69.3	Canada	3 M	60	3	20	20	Supervised aerobic training, 3 sessions/week for 3 months (10-min warm-up; 40-min continuous treadmill/cycle exercise; 10-min cool-down stretching) at 60–75% heart rate reserve (Karvonen method), supervised by a clinical exercise physiologist.	Supervised low-intensity non-aerobic activity (gentle core exercises and very light dumbbell work, non-progressive), avoiding aerobic components.	FPG、SBP、HR
Kiwol Sung (59)	2012	70	Korea	6 M	50	3	22	18	Structured walking program, 3 sessions/week for 50 min/session (5–10 min warm-up; 30–40 min progressive walking at 55–75% HRmax, RPE 11–15; 5-min cool-down), plus dietary education (weekly, 20 min/session for 4 weeks) and diabetes complications management education (weekly, 20 min/session for 4 weeks).	Routine outpatient follow-up and usual diabetes care; no structured walking program or systematic education intervention.	HbA1c、FPG
Hwi Ryun Kwon (60)	2010	56.4	Korea	3 M	60	3	13	15	12-week low-intensity resistance training using elastic bands, 3 sessions/week (~60 min/session), at 40–50% 1RM with gradual progression; 3 sets of 10–15 repetitions targeting upper limb, lower limb, and trunk muscles; 10-min warm-up and 10-min cool-down.	Usual lifestyle and standard pharmacotherapy (stable metformin dose) without structured exercise.	HbA1c
Maryam Nadi (61)	2019	55	Iran	3 M	30	3	15	15	12-week low-intensity resistance training, 3 sessions/week, including 10-min warm-up and 10-min cool-down; multi-joint resistance exercises at 30% 1RM with progression matched to the EPN group.	Usual daily activities only; no structured exercise.	HbA1c、HR、SBP、FPG
R C Plotnikoff (62)	2010	55	Australia	4 M	45	3	27	21	16-week home-based multicomponent progressive resistance training, 3 sessions/week (non-consecutive days), using a multigym and dumbbells; tapering home visits by an accredited exercise professional (18 supervised sessions total). Intensity progressed from 50–60% 1RM (week 1) to 70–85% 1RM; 8 exercises, 3 sets of 8–12 repetitions; 60–120 s rest.	Usual lifestyle and routine medical management; no structured resistance training.	HbA1c、FPG、SBP
Lauren M Sparks (63)	2013	57.6	Netherlands	9 M	50	3	12/18/12	10	AT: Moderate-intensity aerobic training, 12 kcal/kg/week (~150 min/week) at 50–80% VO₂peak, including warm-up and cool-down. RT: Supervised progressive resistance training, 3 sessions/week (~45–50 min/session), 4 upper-limb exercises, 3 lower-limb exercises, plus core training; 10–12 repetitions per set with load increased after achieving 12 repetitions AT + RT: Combined program, aerobic dose 10 kcal/kg/week at 50–80% VO₂peak plus resistance training.	Usual lifestyle over 9 months; stretching/relaxation advice once weekly; no structured aerobic or resistance training.	HbA1c、VO_2_peak
Vanessa Neves de Oliveira (64)	2012	55	Brazil	3 M	60	3	11/10/10	12	AT: Supervised cycling, 3 sessions/week for 60 min/session, intensity prescribed at lactate threshold heart rate; aerobic duration progressed from 20 to 50 min. RT: Whole-body circuit resistance training, 3 sessions/week: first 2 weeks at 50% 1RM (2 sets × 10 reps), then 4 sets × 8–12 reps with progressive increases to volitional fatigue. AT + RT: Combined training, 3 sessions/week: aerobic at lactate threshold for half the AT duration (progressed to 25 min) plus resistance training at half the RT volume (2 sets × 15 reps at 8–12 RM intensity).	Low-intensity stretching sessions 3 times/week, plus health behavior counseling; no structured aerobic or resistance training.	HbA1c、VO_2_peak、SBP、FPG
Alireza Mehdizadeh (65)	2016	58	Iran	3 M	60	3	10/10/10	10	AT: Treadmill training, 3 sessions/week; after a 10-min warm-up, intensity progressed from 40–50% HRmax to 70–80% HRmax for 45–50 min, monitored via heart rate. **RT:** Progressive resistance training, 3 sessions/week (~60 min/session). AT **+ RT:** Alternating combined schedule matched to the single-modality prescriptions: during the first 2 weeks, 2 aerobic + 1 resistance session/week;	Usual lifestyle and medication; no structured exercise.	HbA1c
Giorgio Orlando (66)	2023	55	UK	2 M	50	3	5	7	Supervised combined resistance training plus high-intensity interval training (HIIT), 3 sessions/week for 8 weeks (~75 min/session): ~50 min resistance training as per RT arm (70–80% 1RM; 3 sets × 10 reps plus one set to failure), followed by cycling HIIT (10 × 1 min at 90% HRmax with 1 min active recovery at 30–40% HRmax).	Supervised high-intensity progressive resistance training, 3 sessions/week for 8 weeks (24 sessions), ~50 min/session plus warm-up/cool-down; 6 exercises (e.g., unilateral leg extension, unilateral step-up, lat pulldown, chest press), 3 sets × 10 reps at 70–80% 1RM plus one set to failure; 2-min rests; load increased when ≥12 reps were achieved.	VO_2_peak
Hwi Ryun Kwon (67)	2011	57	Korea	3 M	60	5	13/12	15	AT: Moderate-intensity brisk walking, 5 sessions/week for 60 min/session, monitored using an accelerometer (3.6–6.0 METs); weekly hospital visit during the first 4 weeks, then follow-up every 2 weeks for the remaining 8 weeks. RT: Elastic-band resistance training, 3 sessions/week for 60 min/session (10-min warm-up; 40-min resistance; 10-min cool-down), 3 sets of 10–15 reps per exercise with gradual resistance progression (~40–50% intensity), targeting upper limb, lower limb, and core.	Usual lifestyle without structured exercise.	HbA1c
Y H Ku (68)	2010	56	Korea	3 M	60	5	15/13	16	AT: Moderate-intensity walking, 5 sessions/week for 60 min/session (3.6–5.2 METs). RT: Moderate-intensity elastic-band resistance training, 5 sessions/week; 10 exercises, 3 sets of 15–20 repetitions.	Sedentary lifestyle maintained with standard diabetes education only; no structured exercise	HbA1c、FPG
Niloufar Ghadamyari (69)	2025	56	Iran	2 M	60	3	10/10	10	AT: Supervised moderate-intensity endurance training, 3 sessions/week; intensity progressed from 50–60% to 70–75% HRmaxR; included 10-min warm-up and 5-min cool-down, with heart rate monitoring. RT: Supervised progressive resistance training, 3 sessions/week; 9 machine-based exercises, 3 sets of 8–9 repetitions; intensity progressed from 50–60% to 70–75% 1RM; ~60 min/session; 10-s rests; included 10-min treadmill warm-up.	Sedentary lifestyle and habitual diet maintained; no structured exercise.	VO_2_peak、SBP、HR
C Blioumpa (70)	2023	60.5	Greece	1.5 M	60	3	11	11	Home-based, real-time video-supervised exercise, 3 sessions/week for 60 min/session: 10-min warm-up; 20-min continuous aerobic exercise at 60–80% HRR (Karvonen method); 20-min resistance training (RPE 13–14; 2 sets × 10 reps targeting upper/lower limbs and core); 10-min cool-down.	No supervised exercise for 6 weeks; participants received only general diabetes self-management materials (exercise and diet) in oral and written form.	HbA1c
Karolina S Khan (71)	2022	62	Denmark	3 M	60	2	13	17	Supervised progressive resistance training, 2–3 sessions/week (30 sessions total), ~60 min/session; loads prescribed from individual 1RM and adjusted periodically to maintain the target intensity; supervised by two instructors.	No structured resistance training; usual lifestyle and standard medical care maintained.	HbA1c
Francesca Galle (72)	2019	63.5	Italy	9 M	60	3	69	90	Supervised training 2–3 sessions/week, 60 min/session: 5-min warm-up; 20-min moderate-to-vigorous interval walking (Borg 12–17); 20-min circuit resistance training (20–30 repetitions per exercise).	Usual physical activity advice and routine health education; no supervised exercise or motivational program.	HbA1c
Nafiseh Ghodrati PhD (73)	2023	57.52	Iran	3 M	65	3	12	9	Supervised multicomponent exercise, 3 sessions/week (~65 min/session), including 10-min warm-up and 20-min aerobic training (other components as reported).	No structured exercise during the 12-week period; usual lifestyle maintained and participants were instructed not to initiate any new exercise program.	HbA1c、VO_2_peak、FPG

### 3.2 Risk of bias assessment

**Figure 2:**
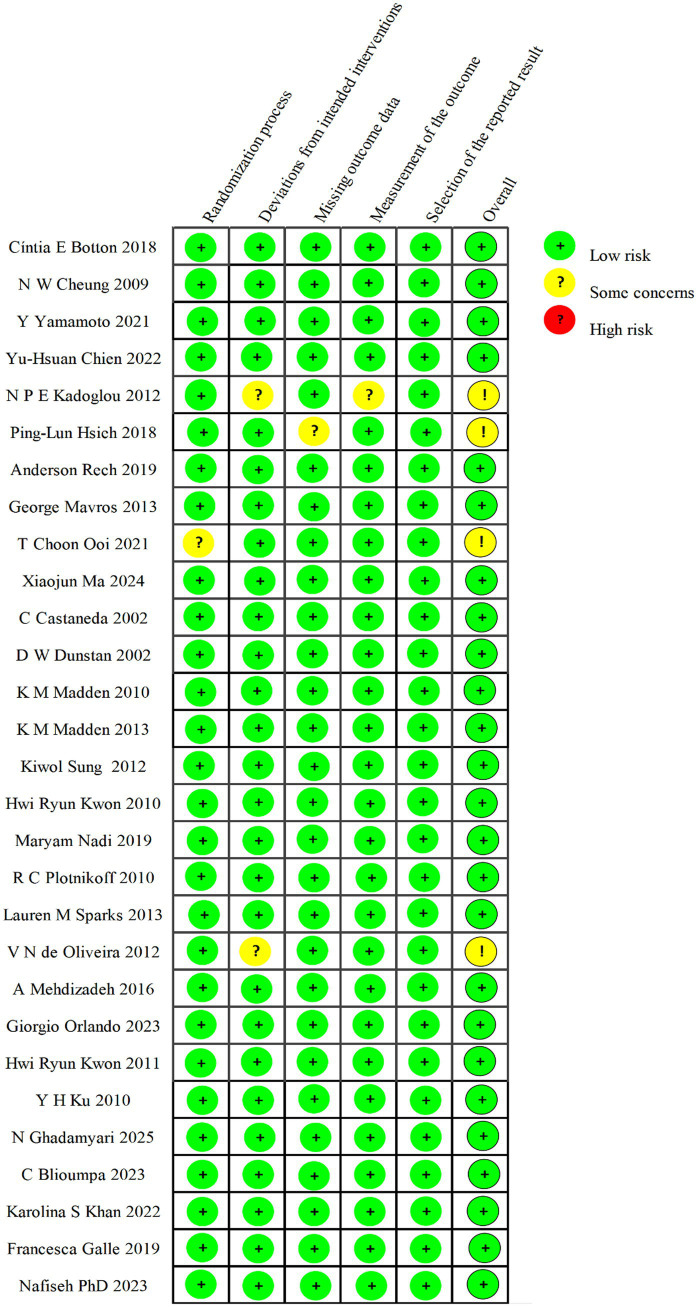
Risk of bias assessment.

To gauge the risk of bias, the researchers turned to the renowned Cochrane Risk of Bias 2 tool. Among the 29 included trials, 25 were judged to have low overall risk of bias. However, there were a handful—four to be precise—that raised some flags and prompted further contemplation. In general, most trials reported clearly defined randomization procedures, implemented the interventions as planned, achieved acceptable data completeness, and used largely objective outcome measures, indicating overall good methodological quality. When it comes to the randomization process, the majority of studies showcased solid allocation concealment and relied on random number tables or computer-generated methods for randomization. Yet, a few trials failed to adequately describe their sequence generation and/or allocation concealment, potentially raising the risk of selection bias. As for staying true to the intended interventions, most trials took an intention-to-treat approach, which is a great way to reduce bias that might arise from participants not sticking to the protocol. However, in some cases, participant blinding was impossible, and at best, trials were only single-blinded, which leaves room for performance bias due to participants changing their behavior based on their group assignment. For missing outcome data, outcome reporting was generally complete, and when attrition occurred, it was commonly handled using intention-to-treat principles, resulting in a low risk of bias in this domain. A minority of studies, however, did not provide an adequate strategy for dealing with missing data. In terms of outcome measurement, most trials employed objective assessment methods and implemented blinding at least at the outcome assessment stage, suggesting a low overall risk of measurement bias. Across the board, the potential for bias remained minimal throughout the various domains, while the evidence quality was deemed sufficiently robust to lend credence to the study’s outcomes. A comprehensive breakdown of RoB 2 evaluations can be found in Figure 2.

### 3.3 Network meta-analyses

#### 3.3.1 Assessment of consistency and inconsistency

**Figure 3:**
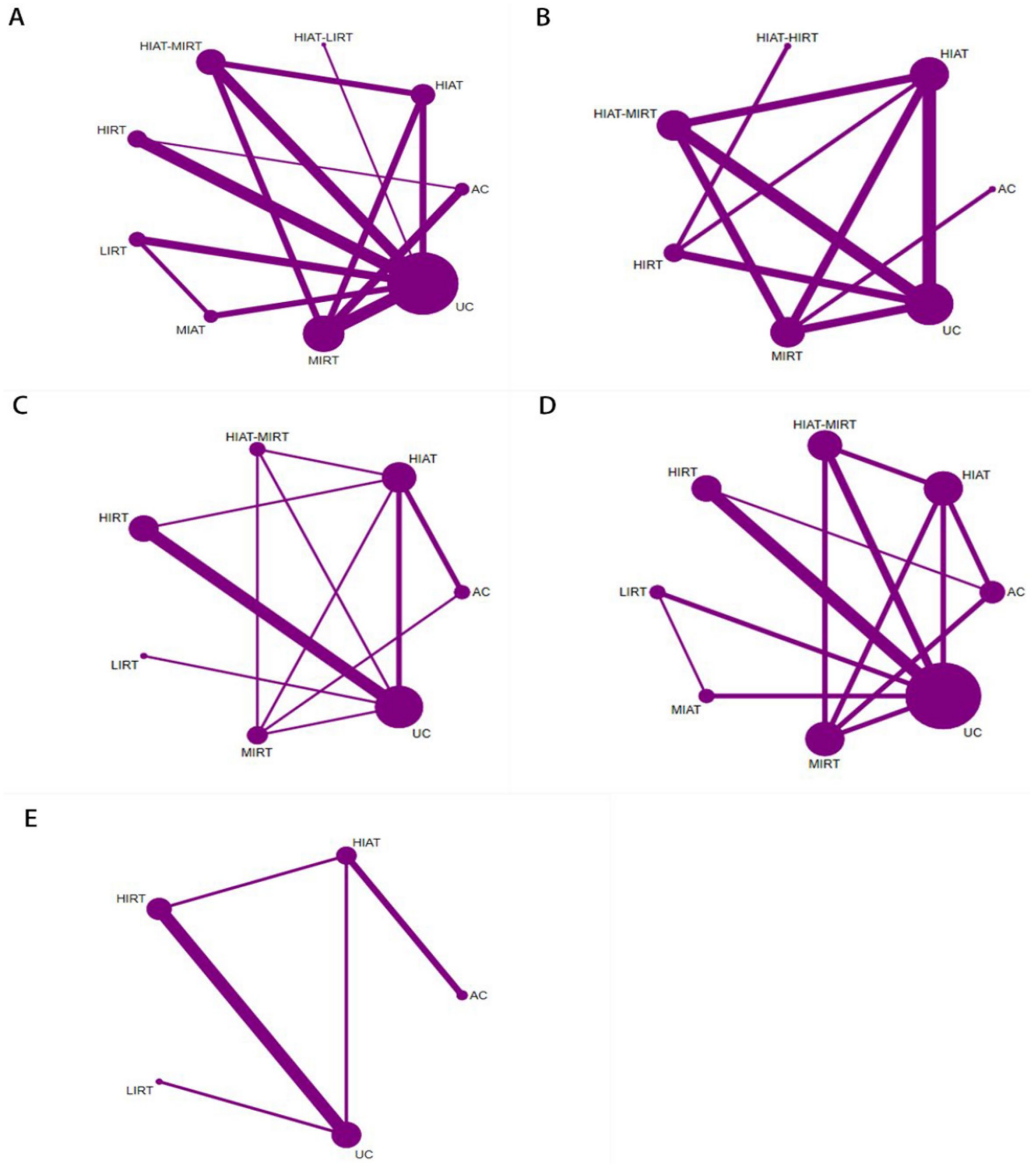
Network plots of different aerobic and resistance exercise intensities in middle-aged and older adults with type 2 diabetes. **(A)** HbA1c; **(B)** VO_2_peak; **(C)** SBP; **(D)** FPG; **(E)** HR.

Primary endpoints included HbA1c and VO₂peak, with SBP, FPG, and resting HR considered secondary endpoints. For each outcome, the network geometry contained at least one closed loop (Figure 3), allowing formal evaluation of the consistency assumption. The evaluation of worldwide discrepancies was conducted through the analysis of the treatment-by-design interaction. For all outcomes, the corresponding *p*-values exceeded 0.05 (Supplementary Table S3), indicating no statistically significant evidence against the assumption of consistency. The node-splitting method served as a follow-up investigation into local inconsistency in our analysis. For all pairwise comparisons conducted, the *p*-values remained above the 0.05 threshold (see Supplementary Tables S4–S8), which indicates that direct and indirect estimates were in lockstep with one another. Finally, loop-specific inconsistency was evaluated by calculating inconsistency factors (IFs). Loop-specific inconsistency was further evaluated by calculating inconsistency factors (IFs) and their 95% confidence intervals. For most closed loops, the 95% confidence intervals of the IFs included zero, suggesting no clear evidence of loop-specific inconsistency. However, for the FPG outcome, the 95% confidence interval of the IF for the LIRT–MIAT–UC loop did not include zero, indicating potential loop-specific inconsistency. Therefore, network estimates involving FPG, particularly those related to this loop, should be interpreted with caution. For the remaining outcomes, the 95% confidence intervals of the IFs for all identified loops included zero, supporting the overall consistency of the corresponding network models (see Supplementary Tables S9–S13). When you put all these results together. Taken together, these findings support the consistency assumption of the network model across outcomes.

#### 3.3.2 Assessment of transitivity

The distribution of potential effect modifiers was examined across the treatment network before interpreting indirect comparisons. Across the 29 included trials, mean participant age ranged from 55 to 72.9 years, and follow-up duration ranged from 1.5 to 12 months. Most interventions were delivered three times per week, although exercise frequency ranged from two to seven sessions per week, and session duration varied from approximately 15 to 65 min. Baseline glycemic status was broadly comparable across studies, with all trials enrolling participants with type 2 diabetes, although baseline HbA1c levels and diabetes duration varied across populations. Medication background was generally reported as stable or unchanged during the intervention period in most trials, but the level of detail varied across studies.

Training supervision also varied across trials, including supervised, home-based, and partially supervised interventions. Dietary or nutritional co-interventions were uncommon, but some trials included dietary education or nutritional supplementation. Comparator conditions included usual care, no structured exercise, health education, stretching, sham exercise, and low-intensity activities. These comparator conditions were classified according to their methodological role in the trial design, and trial-arm-level details are provided in Supplementary Table S60.

**Figure 6:**
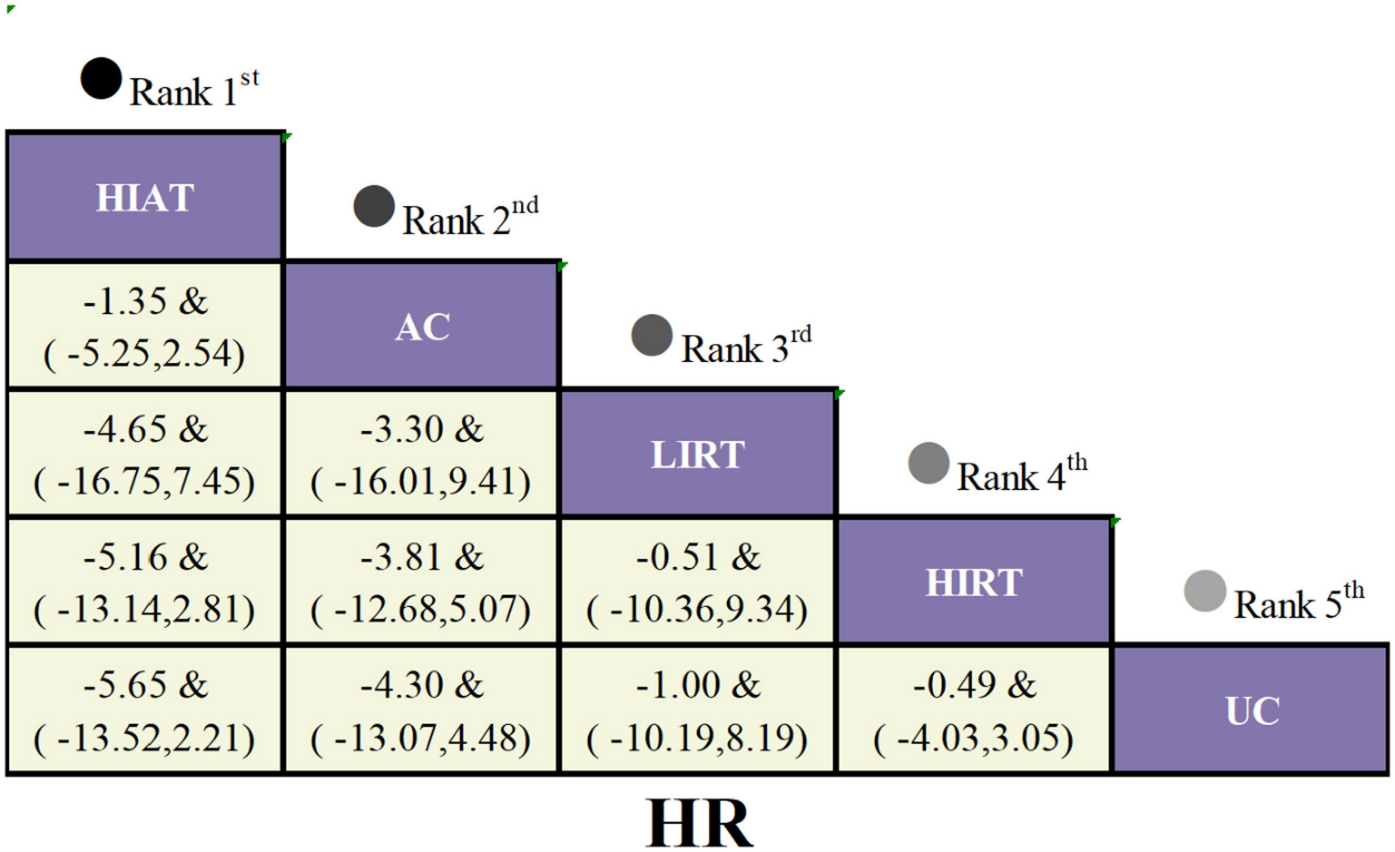
The table below showcases the comparative treatment effects of varying exercise intensities for middle-aged and older adults individuals diagnosed with type 2 diabetes, taking resting heart rate into account. The level of certainty in the evidence has been evaluated according to the GRADE methodology, and is denoted as follows: # indicates high certainty; & denotes moderate certainty; * signifies low certainty; $ represents very low certainty.

Univariable network meta-regression analyses were conducted for available study-level covariates, including geographical region, follow-up duration, mean age, baseline HbA1c, session duration, and weekly training frequency. None of these covariates showed a statistically significant association with treatment effects, suggesting that the available evidence did not identify strong effect modification by these factors. However, because some clinically relevant variables, including medication background, diabetes duration, supervision level, dietary co-interventions, and comparator characteristics, were inconsistently reported, residual imbalance across comparisons cannot be fully excluded (Figures 3–6).

**Figure 7:**
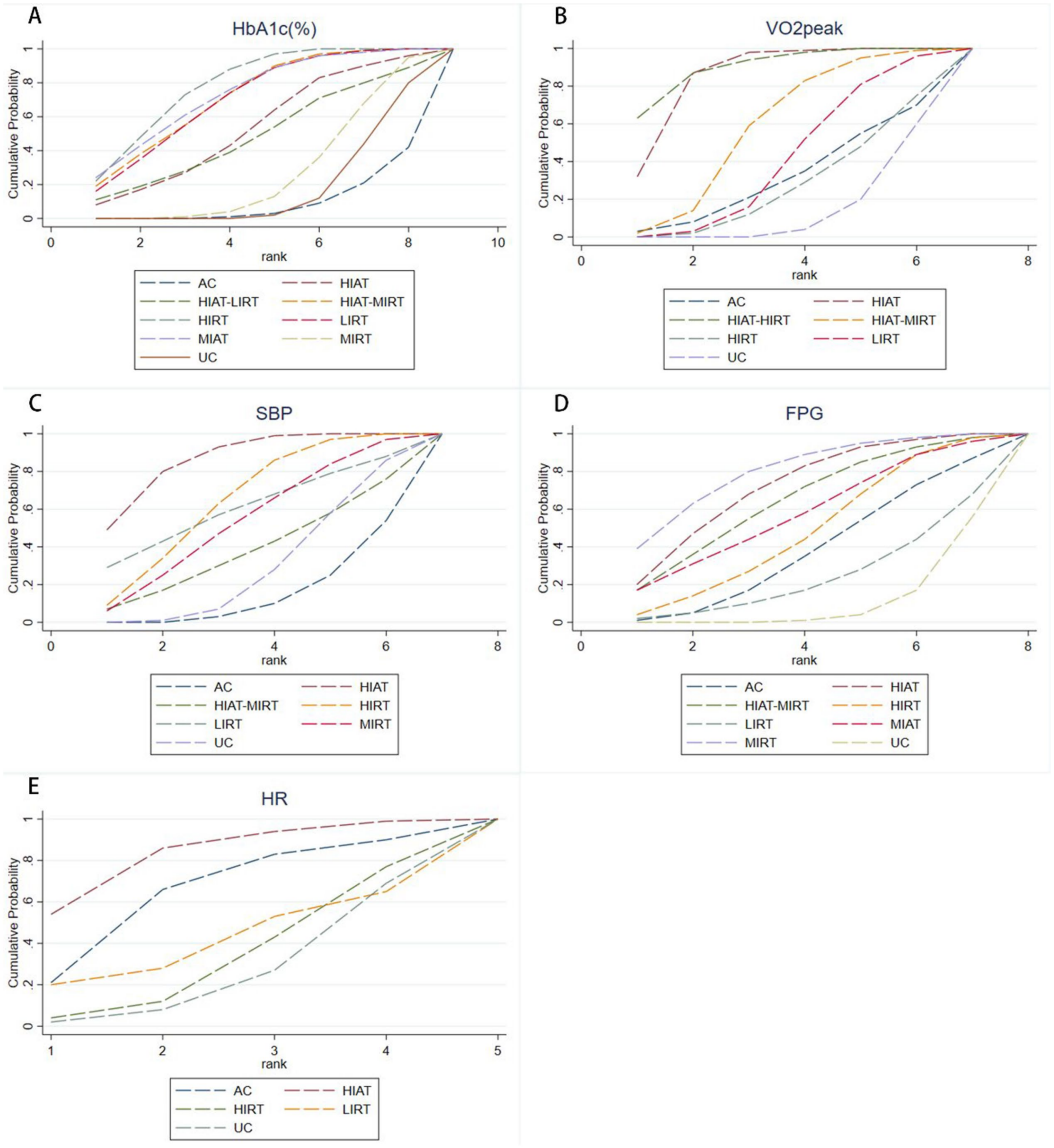
Surface under the cumulative ranking curve (SUCRA) plots for exercise interventions of different intensities across outcomes in middle-aged and older adults with type 2 diabetes: **(A)** HbA1c; **(B)** VO_2_peak; **(C)** SBP; **(D)** FPG; **(E)** resting HR.

#### 3.3.3 HbA1c

**Figure 4:**
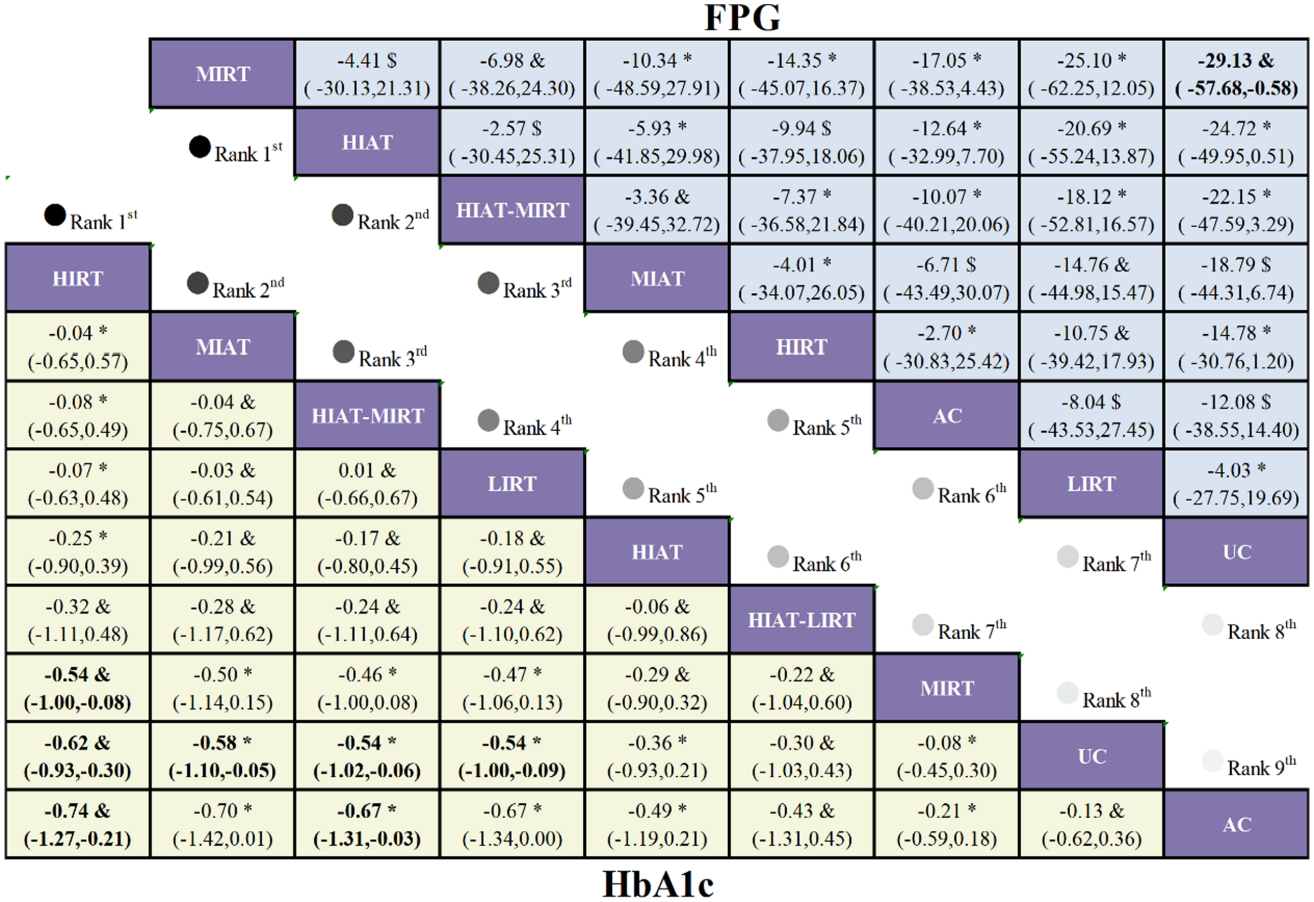
In this study, we present a league table featuring pairwise comparisons of exercise interventions at varying intensities for middle-aged and older adults individuals with type 2 diabetes. The bottom-left quadrant, highlighted in yellow, showcases the outcomes related to HbA1c levels, while the top-right quadrant, marked in blue, displays findings concerning FPG. The level of evidence certainty is denoted as follows: # signifies high certainty, and denotes moderate certainty, * indicates low certainty, and $ represents very low certainty, all in accordance with the GRADE system.

Twenty-five randomized controlled trials evaluating nine interventions contributed to the HbA1c network (Figure 3A). Moderate certainty evidence indicated that, compared with UC, HIRT(MD − 0.62, 95% CI − 0.93 to −0.30), low-certainty evidence indicated that MIAT(MD − 0.58, 95% CI − 1.10 to −0.05), low-certainty evidence indicated that HIAT–MIRT(MD − 0.54, 95% CI − 1.02 to −0.06), and low-certainty evidence indicated that LIRT(MD − 0.54, 95% CI − 1.00 to −0.09) were associated with statistically significant reductions in HbA1c in middle-aged and older adults with type 2 diabetes (Figure 4). Although low-certainty evidence indicated that HIAT (MD − 0.36, 95% CI − 0.93 to 0.21) and MIRT (MD − 0.08, 95% CI − 0.45 to 0.30) showed favorable point estimates, these differences did not reach statistical significance. Ranking based on SUCRA values indicated that HIRT had the highest probability of being the ranked highest (78.6%), followed by MIAT (73.3%) and HIAT–MIRT (71.4%). However, the probability analysis indicated that MIAT had the highest likelihood of being the ranked highest (best-treatment probability, 24.0%), followed by HIRT (22.2%) and HIAT–MIRT (19.4%). Thus, although HIRT ranked highly overall, MIAT may be the may warrant further investigation under certain circumstances. This pattern was further supported by the mean rank analysis, which likewise highlighted the relative advantages of HIRT and MIAT: MIAT ranked first with the lowest mean rank (2.7), followed by HIRT (3.1) and HIAT–MIRT (3.3) (Figure 7 and Supplementary Table S14).

#### 3.3.4 FPG

Sixteen randomized controlled trials encompassing eight interventions contributed to the FPG network (Figure 3D). Moderate certainty evidence indicated that compared with UC, only MIRT was associated with a statistically significant reduction in fasting plasma glucose (MD − 29.13 mg/dL, 95% CI − 57.68 to −0.58; Figure 4). Low-certainty evidence indicated that HIAT (MD − 24.72 mg/dL, 95% CI − 49.95 to 0.51) and HIAT–MIRT (MD − 22.15 mg/dL, 95% CI − 47.59 to 3.29) showed favorable point estimates but did not achieve statistical significance. SUCRA-based ranking indicated that MIRT had the highest probability of being the ranked highest (80.5%), followed by HIAT (72.5%) and HIAT–MIRT (65.1%). However, probability analysis indicated that MIRT had the highest likelihood of being the ranked highest (best-treatment probability, 39.4%), followed by HIAT (19.6%) and MIAT (17.4%). MIRT also remained highly ranked overall, further suggesting that it may represent the may be a promising option, but should be interpreted cautiously. This pattern was reinforced by the mean rank analysis, which likewise demonstrated the relative advantages of MIRT and HIAT: MIRT ranked first with the lowest mean rank (2.4), followed by HIAT (2.9) and HIAT–MIRT (3.4) (Figure 7 and Supplementary Table S15). However, because loop-specific analysis identified potential inconsistency in the LIRT–MIAT–UC loop of the FPG network, the FPG estimates and rankings should be interpreted with caution.

#### 3.3.5 VO_2_peak

**Figure 5:**
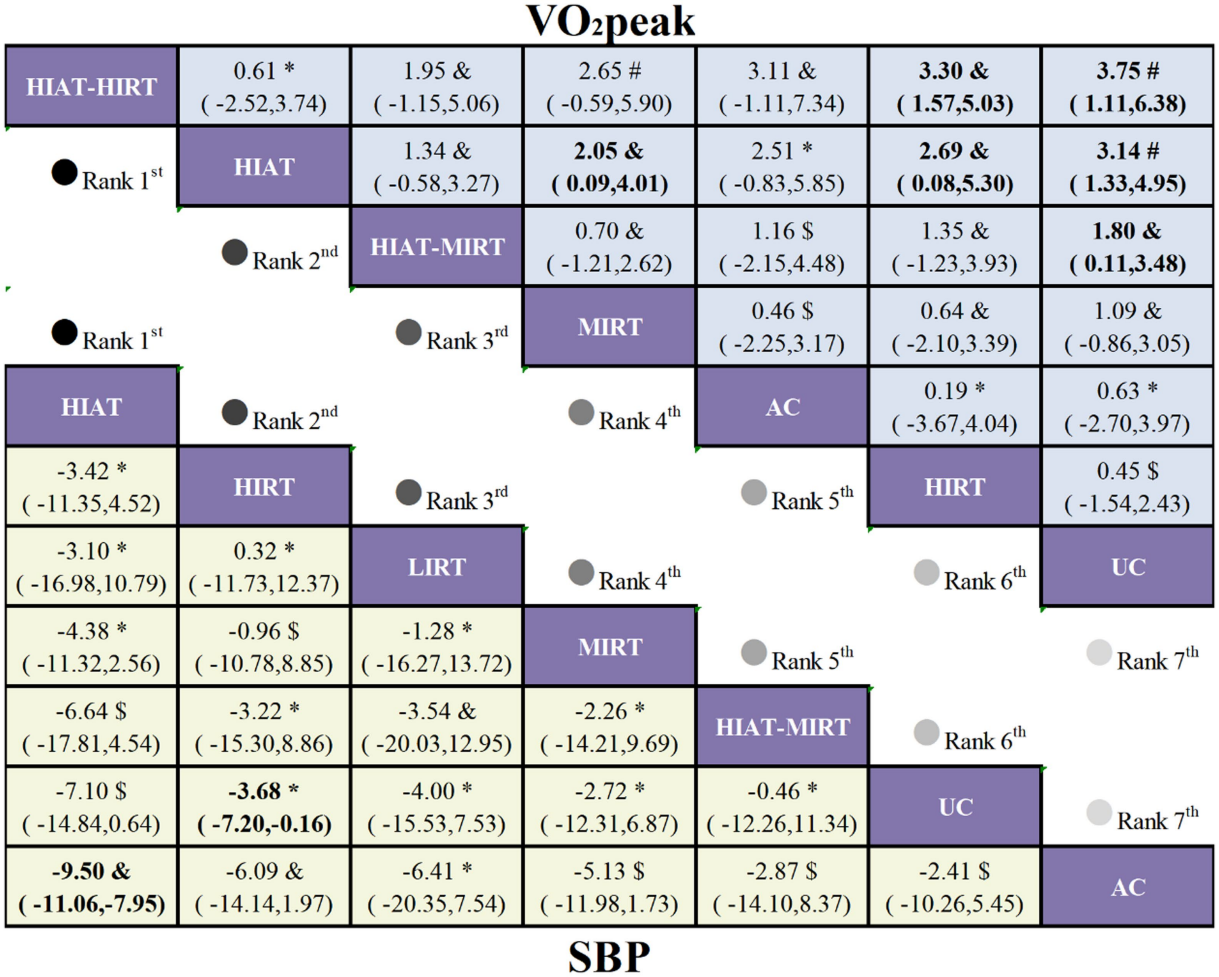
Here's the passage rephrased: the comparison of the relative impact of exercise regimens at different levels of intensity on the middle-aged and older adults with type 2 diabetes, as depicted in a league table, includes SBP assessments displayed in the lower quadrant, highlighted in yellow. In the upper quadrant, which is colored blue, we find VO_2_peak. The level of certainty regarding the study results is denoted according to the GRADE system: ‘#’ denotes high certainty, ‘&’ denotes moderate certainty, ‘*’ indicates low certainty, and ‘$’ signifies very low certainty.

Seven randomized controlled trials involving seven interventions informed the VO₂peak analysis (Figure 3B). High certainty evidence indicated that relative to UC, HIAT–HIRT (MD 3.75 mL·kg^−1^·min^−1^, 95% CI 1.11 to 6.38), HIAT alone (MD 3.14 mL·kg^−1^·min^−1^, 95% CI 1.33 to 4.95), and moderate certainty evidence indicated that HIAT–MIRT (MD 1.80 mL·kg^−1^·min^−1^, 95% CI 0.11 to 3.48) produced statistically significant improvements in VO₂peak (Figure 5). Although moderate certainty evidence indicated that MIRT (MD 1.09 mL·kg^−1^·min^−1^, 95% CI − 0.86 to 3.05) and very low certainty evidence indicated that HIRT (MD 0.45 mL·kg^−1^·min^−1^, 95% CI − 1.54 to 2.43) were associated with increases in VO₂peak, these effects were not statistically significant. Based on SUCRA values, HIAT–HIRT ranked highest (90.3%), followed by HIAT (85.9%) and HIAT–MIRT (58.8%). However, probability analysis indicated that HIAT–HIRT had the highest likelihood of being the ranked highest (best-treatment probability, 63.4%), followed by HIAT (31.7%) and AC (2.8%). HIAT–HIRT ranked highly across SUCRA, best-treatment probability, and mean rank analyses; however, because the VO₂peak network was sparse and informed by only seven trials, these rankings should be interpreted as exploratory rather than definitive. This pattern was further supported by the mean rank analysis, which likewise highlighted the relative advantages of HIAT–HIRT and HIAT: HIAT–HIRT ranked first with the lowest mean rank (1.6), followed by HIAT (1.8) and HIAT–MIRT (3.5) (Figure 7 and Supplementary Table S16).

#### 3.3.6 SBP

Ten randomized controlled trials involving seven interventions contributed to the SBP network (Figure 3C). Low certainty evidence indicated that compared with UC, only HIRT significantly reduced SBP (MD − 3.68 mmHg, 95% CI − 7.20 to −0.16; Figure 5). Very low certainty evidence indicated that HIAT (MD − 7.10 mmHg, 95% CI − 14.84 to 0.64) and low certainty evidence indicated that MIRT (MD − 2.72 mmHg, 95% CI − 12.31 to 6.87) showed reductions in SBP relative to UC, but the confidence intervals included the null. SUCRA rankings suggested that HIAT had the highest probability of being most effective (86.7%), followed by HIRT (64.7%) and MIRT (54.2%). However, probability analysis indicated that HIAT had the highest likelihood of being the ranked highest (best-treatment probability, 49.0%), followed by LIRT (28.9%) and HIRT (9.1%). Thus, although HIRT ranked highly overall, HIAT may be the may warrant further investigation in specific contexts. This pattern was further supported by the mean rank analysis, which likewise demonstrated the relative advantages of HIAT and HIRT: HIAT ranked first with the lowest mean rank (1.8), followed by HIRT (3.1) and LIRT (3.4) (Figure 7 and Supplementary Table S17).

#### 3.3.7 HR

Seven randomized controlled trials comprising five interventions informed the resting HR analysis (Figure 3E). Moderate certainty evidence indicated that compared with UC, HIAT showed a modest reduction in resting HR (MD − 1.35 beats/min, 95% CI − 5.25 to 2.54), but this difference was not statistically significant (Figure 6). Given the small number of trials and sparse network structure, the HR findings should be interpreted cautiously, and treatment rankings for this outcome should be considered exploratory.

### 3.4 Sensitivity analyses, network meta-regression, and publication bias

A leave-one-out sensitivity analysis was performed for all outcomes, scrutinizing the impact of individual studies on the network estimates. During each round, a single study was omitted, and the random-effects network meta-analysis was rerun. Regardless of the iterations, the direction of the combined effects for both aerobic and resistance training, compared to standard care, stayed consistent. The magnitude of these effects varied only slightly, with the confidence intervals displaying considerable overlap, and the statistical significance remained unaffected. This suggests that the outcomes were resilient to the exclusion of any single study (see Supplementary Tables S19–S23). In addition, sensitivity analyses were performed using *R* values of 0.25 and 0.75 to reassess the effect estimates for each intervention relative to the control group and to determine whether the direction of effect changed under alternative assumptions. The direction of effect remained materially unchanged across all comparisons, indicating that the findings were robust (see Supplementary Tables 59).

To further explore the potential sources of variation, we conducted univariate network meta-regression analyses using geographical area, follow-up duration, mean age of participants, baseline HbA1c level, exercise session duration, and weekly training frequency as covariates. None of these factors demonstrated a significant link to treatment outcomes when compared to the control group, as evidenced in the Supplementary Tables S24 through 38 and Supplementary Tables 44 through 58. We created adjusted funnel plots for HbA1c, fasting plasma glucose, VO_2_ peak, systolic blood pressure, and heart rate to gauge any potential publication bias. The study effects were spread evenly without any notable extremes, pointing to a slim chance of major publication bias, as illustrated in the Supplementary Figures S1–S5.

### 3.5 Subgroup analysis

Subgroup analyses were performed in middle-aged and older adults with type 2 diabetes aged >60 years to determine which interventions were associated with statistically significant differences in diabetes-related outcomes. Compared with the control group, HIRT was associated with significant improvements in FPG and HbA1c in studies involving participants aged >60 years. These findings suggest that, in middle-aged and older adults with type 2 diabetes, HIRT may confer greater benefits for both short-term glycemic regulation and long-term glycemic control (Supplementary Figure S6).

### 3.6 GRADE assessment

The certainty of evidence for the primary outcomes was evaluated using the CINeMA framework. The distribution of certainty ratings varied across outcomes (Supplementary Tables S39–S43). For HbA1c, among 36 comparisons, 19 (53%) were rated as moderate certainty and 17 (47%) as low certainty. For FPG, across 28 comparisons, 5 (18%) were moderate certainty, 16 (57%) low certainty, and 7 (25%) very low certainty. For VO₂peak (21 comparisons), 3 (14%) were high certainty, 11 (53%) moderate certainty, 4 (19%) low certainty, and 3 (14%) very low certainty. For SBP (21 comparisons), certainty was generally lower, with 3 (14%) moderate certainty, 12 (57%) low certainty, and 6 (29%) very low certainty. For resting HR (10 comparisons), all comparisons were rated as moderate certainty. Overall, the certainty of evidence ranged from moderate to very low across outcomes, with higher confidence observed primarily for VO₂peak comparisons and comparatively lower certainty for SBP and FPG.

## 4 Discussion

This network meta-analysis synthesized evidence from 29 randomized controlled trials involving 1,301 middle-aged and older adults with type 2 diabetes across 14 countries. We compared 10 exercise modalities defined by intensity and training mode and evaluated their relative effects on HbA1c, FPG, VO₂peak, SBP, and resting HR within a consistency-based random-effects framework. Participants had mean ages ranging from 55 to 72.9 years, and follow-up periods were predominantly 1.5–12 months; accordingly, our findings primarily reflect short- to mid-term intervention effects.

### 4.1 Principal findings and clinical interpretation

HbA1c Compared with usual care, HIRT, MIAT, HIAT–MIRT, and LIRT were associated with statistically significant reductions in HbA1c. In contrast, HIAT and MIRT showed favorable but non-significant trends. These results suggest that glycemic improvement in older adults with type 2 diabetes is influenced by both training intensity and modality. High-intensity resistance training may confer superior glycemic benefits through enhanced skeletal muscle glucose uptake and improved insulin sensitivity, given the central role of muscle mass and contractile activity in glucose disposal (34, 35). The statistically significant effect observed with MIAT—but not HIAT—may reflect better tolerability and adherence in this age group, where sustained participation is critical for metabolic benefit (36). Notably, combined aerobic and resistance training also produced significant HbA1c reductions, plausibly through complementary effects on peripheral and hepatic insulin resistance (20). For fasting plasma glucose, only MIRT achieved a statistically significant reduction relative to usual care and ranked highest by SUCRA. This pattern suggests that FPG may be particularly responsive to interventions that improve basal insulin sensitivity rather than those primarily increasing acute energy expenditure. Moderate-intensity resistance training has been shown to enhance skeletal muscle glucose transport capacity and insulin-mediated glucose clearance, mechanisms directly relevant to fasting glycemia (34, 35). In older populations, moderate intensities may also promote better adherence, which is essential for sustained improvements in fasting metabolic control (36). VO₂peak Interventions centered on high-intensity aerobic training—particularly HIAT–HIRT and HIAT alone—demonstrated the most pronounced improvements in VO₂peak. These findings are physiologically plausible. High-intensity aerobic stimuli induce robust central cardiovascular adaptations and peripheral mitochondrial remodeling, both of which are key determinants of maximal oxygen uptake (37, 38). The addition of resistance training may further support these adaptations by improving muscular strength and exercise tolerance, thereby enabling higher-quality aerobic stimulus over time (39). SBP, Only HIRT significantly reduced systolic blood pressure relative to usual care. This finding suggests that blood pressure reduction in this population may depend on achieving sufficient training intensity. High-intensity resistance exercise may promote favorable vascular and autonomic adaptations, including improved arterial compliance and endothelial function—both critical determinants of systolic pressure (40, 41). Lower-intensity protocols may not provide an adequate stimulus within relatively short intervention periods (36). Evidence for resting HR was limited (seven trials), and no intervention demonstrated a statistically significant reduction relative to usual care, although HIAT ranked highest. Resting HR may be less responsive to exercise in older adults with type 2 diabetes, particularly given the high prevalence of autonomic dysfunction and the frequent use of rate-modifying medications in this population (36, 42). The relatively small number of trials and short follow-up durations further reduce statistical power. The favorable SUCRA ranking for HIAT likely reflects consistency in the direction of effect rather than a clearly superior intervention effect, underscoring the need for cautious interpretation (43).

### 4.2 Comparison with previous meta-analyses

Our findings align in part with prior conventional meta-analyses. Feng et al. reported that resistance training improves HbA1c and FPG in middle-aged and older adults with type 2 diabetes (44). However, that analysis included fewer trials and did not distinguish between training intensities. In contrast, our network meta-analysis incorporated a broader range of aerobic and resistance modalities and identified potential intensity-dependent differences—HIRT appearing most favorable for HbA1c reduction and MIRT for FPG. Similarly, Mercede et al. concluded that aerobic exercise improves HbA1c, FPG, and SBP and applied GRADE to assess certainty of evidence (45). However, aerobic exercise was analyzed as a single category without stratification by intensity. By integrating multiple intensities and training modes within a unified analytical framework, our study provides more granular insight into the relative performance of specific exercise prescriptions. Importantly, we also extended the scope beyond traditional metabolic outcomes by incorporating VO₂peak, thereby capturing functional and cardiorespiratory adaptations. This broader outcome framework offers a more comprehensive perspective on the health impact of exercise in older adults with type 2 diabetes and enhances the clinical relevance of exercise prescription decisions.

### 4.3 Strengths and clinical implications

Across outcomes, distinct but cautiously interpreted patterns emerged. HIRT, MIAT, HIAT–MIRT, and LIRT were associated with reductions in HbA1c versus usual care, with certainty ranging from moderate to low. MIRT was associated with a reduction in FPG, but interpretation is limited by potential loop-specific inconsistency. HIAT–HIRT and HIAT were associated with improvements in VO₂peak, although these findings were based on a sparse evidence network and should be considered exploratory. HIRT was the only intervention associated with a statistically significant reduction in SBP, supported by low-certainty evidence. Overall, these findings may help inform individualized exercise prescription, but they should not be interpreted as establishing a single clinically superior intervention across outcomes. Especially if the aim is to beef up muscle strength, boost insulin action, and keep blood pressure in check. Aerobic training, particularly at higher intensities when feasible, may be layered onto resistance training to optimize cardiorespiratory fitness and overall metabolic benefit. Exercise prescriptions should remain individualized. Comorbidities common in this age group—such as cardiovascular disease, osteoarthritis, and reduced exercise tolerance—necessitate careful intensity stratification and professional supervision to maximize benefit while minimizing risk. Methodologically, strengths of this study include the focus on a clinically high-burden population, inclusion of multinational samples, application of a network meta-analytic framework enabling both direct and indirect comparisons, formal consistency assessment, sensitivity analyses, network meta-regression, and structured certainty evaluation using CINeMA. Notably, certainty was lower for FPG and SBP comparisons, warranting cautious interpretation of those findings.

### 4.4 Limitations

Multiple limitations are worth noting. Initially, it’s nearly impossible to completely blind exercise interventions, which can lead to potential biases in performance and detection. Nonetheless, the majority of the primary outcomes were evaluated in an objective manner, which helps ease these worries. Additionally, the evidence for resting heart rate was scarce and lacked substantial power. The absence of statistically significant differences likely reflects both physiological and methodological factors, including autonomic impairment, medication effects, small sample sizes, and short intervention durations. Definitive conclusions regarding HR cannot therefore be drawn. Third, follow-up durations were generally limited to 1.5–12 months, precluding assessment of long-term sustainability and adverse event profiles. Reporting of adherence and safety was inconsistent across trials. Fourth, although older adulthood is conventionally defined as ≥60 or ≥65 years, we adopted a mean age threshold of ≥55 years. This approach may introduce some heterogeneity in age classification; however, many trials in this field enroll participants with mean ages between 55 and 58 years. Applying stricter criteria would have substantially reduced the available evidence base. Fifth, another limitation of this network meta-analysis is the sparsity of the evidence network for several comparisons, such as HIAT–LIRT and HIAT–HIRT, for which only one or two studies were available. Sparse connections within the network may reduce the precision and stability of the estimated treatment effects, leading to wider confidence intervals and greater uncertainty in the corresponding comparisons. In addition, network sparsity may weaken the robustness of indirect evidence and reduce confidence in the ranking of interventions. Therefore, findings derived from these sparsely informed comparisons should be interpreted with caution. Sixth, another limitation of the present study is that the long-term effects of exercise interventions were not adequately addressed, as follow-up durations in the included trials were generally limited. Therefore, the current findings primarily reflect short- to medium-term intervention effects, and the sustainability of these benefits beyond 12 months remains uncertain. Future randomized controlled trials with longer follow-up periods are warranted to determine whether the observed improvements can be maintained over time. Another limitation of this study is that, although SBP was included in the quantitative synthesis, DBP was not examined because too few studies reported this outcome to allow a robust network analysis. As a result, the effects of different exercise interventions on DBP remain uncertain and warrant further investigation as more data accumulate. Although global inconsistency tests did not indicate major network-wide incoherence, loop-specific inconsistency analysis suggested potential inconsistency in the LIRT–MIAT–UC loop of the FPG network. This discrepancy may reflect clinical and methodological heterogeneity among trials contributing to FPG, including differences in baseline glycemic control, medication background, exercise supervision, intervention dose, dietary advice, and comparator conditions. Therefore, FPG estimates and rankings, particularly those relying on indirect evidence through this loop, should be interpreted with caution. Although multi-arm trials were appropriately handled using the augment approach to preserve within-study correlations, this method does not overcome the fundamental limitation of a small number of primary studies. For example, in the VO₂peak analysis, a relatively large number of comparisons (*n* = 21) were generated from only seven studies. This may give an impression of a well-connected evidence network, while the underlying evidence base remains limited. Therefore, the stability and interpretability of these results may be constrained, and the findings should be interpreted with caution. A further methodological consideration is the heterogeneity of comparator conditions. In the included trials, comparator arms included usual care, stretching, health education, sham exercise, and low-intensity activities. Although these comparators may not be fully interchangeable from a clinical perspective, further subdivision of these control conditions would have resulted in a highly sparse and fragmented network, with several disconnected or weakly connected nodes, thereby precluding a stable and interpretable network meta-analysis. Therefore, comparator conditions were grouped according to their common function within the trial design: usual care/no-treatment conditions represented routine or minimal care, whereas active control conditions involved participant contact, attention, or low-level activity without meeting the prespecified criteria for structured aerobic or resistance training. This approach was intended to preserve network connectivity while maintaining clinically meaningful distinctions between comparator types. Transitivity was assessed by examining the distribution of potential clinical and methodological effect modifiers across treatment comparisons, including mean age, baseline glycemic status, diabetes duration, medication background, training supervision, intervention duration, exercise frequency, dietary co-interventions, and comparator condition. Although formal inconsistency tests did not show significant disagreement between direct and indirect evidence, we acknowledge that statistical consistency alone does not establish transitivity. Univariable network meta-regression analyses for available study-level covariates, including geographical region, follow-up duration, mean age, baseline HbA1c, session duration, and weekly training frequency, did not identify significant effect modification. However, several potentially important modifiers, such as medication background, diabetes duration, dietary co-interventions, supervision level, and comparator characteristics, were inconsistently reported and could not be fully examined quantitatively. Therefore, residual violations of the transitivity assumption cannot be excluded, and indirect comparisons and rankings should be interpreted with appropriate caution. Moreover, individuals with type 2 diabetes often exhibit earlier functional decline, supporting the clinical relevance of this threshold.

## 5 Conclusion

Exercise interventions produced outcome-specific effects in middle-aged and older adults with type 2 diabetes. HIRT was associated with favorable reductions in HbA1c, whereas MIAT, HIAT–MIRT, and LIRT also showed potentially beneficial effects. MIRT showed a favorable effect on FPG; however, this finding should be interpreted cautiously because loop-specific inconsistency was detected in the FPG network. For VO₂peak, HIAT–HIRT and HIAT appeared to provide the greatest improvements, but these findings were based on a sparse evidence network and should be considered exploratory. HIRT was the only modality with a statistically supported reduction in SBP, while evidence for resting HR remained inconclusive. Future large-scale randomized trials with longer follow-up, standardized outcome definitions, and direct head-to-head comparisons between key exercise modalities are needed to strengthen the certainty of evidence and refine individualized exercise prescription in this population.

## Data Availability

The original contributions presented in the study are included in the article/Supplementary material, further inquiries can be directed to the corresponding author.
