# Supplementary material for: Comparative effects of different intensities of aerobic and resistance exercise on glycemic control and cardiorespiratory fitness in middle-aged older patients with type 2 diabetes: a network meta-analysis
Source: Front Public Health. 2026 May 25;14:1818686. doi: 10.3389/fpubh.2026.1818686 (PMC13243112; doi:10.3389/fpubh.2026.1818686)
Supplement: Supplementary file 1 [file Table_1.docx]

Comparative Effects of Different Intensities of Aerobic and Resistance Exercise on Glycemic Control and Cardiorespiratory Fitness in middle-aged Older Patients with Type 2 Diabetes: A Network Meta-Analysis

| **Table of Contents** | | |
| --- | --- | --- |
| Title | Content | page |
| Table S1 | PRISMA NMA Checklist of Items to Include When Reporting a Systematic Review Involving a Network Meta-analysis | 3-6 |
| Table S2 | Literature Search Strategy | 6-10 |
| Table S3 | Global inconsistency assessment for HbA1c, FPG, VO₂peak, SBP, and resting HR in middle-aged and older adults with type 2 diabetes. | 10 |
| Table S4 | Node-splitting analysis for HbA1c in middle-aged and older adults with type 2 diabetes. | 11 |
| Table S5 | Node-splitting analysis for FPG in middle-aged and older adults with type 2 diabetes. | 11 |
| Table S6 | Node-splitting analysis for VO_2_peak in middle-aged and older adults with type 2 diabetes. | 11-12 |
| Table S7 | Node-splitting analysis for SBP in middle-aged and older adults with type 2 diabetes | 12 |
| Table S8 | Node-splitting analysis for HR in middle-aged and older adults with type 2 diabetes | 12 |
| Table S9 | Loop-specific inconsistency analysis for HbA1c in middle-aged and older adults with type 2 diabetes. | 12 |
| Table S10 | Loop-specific inconsistency analysis for FPG in middle-aged and older adults with type 2 diabetes. | 12-13 |
| Table S11 | Loop-specific inconsistency analysis for VO_2_peak in middle-aged and older adults with type 2 diabetes. | 13 |
| Table S12 | Loop-specific inconsistency analysis for SBP in middle-aged and older adults with type 2 diabetes. | 13 |
| Table S13 | Loop-specific inconsistency analysis for HR in middle-aged and older adults with type 2 diabetes. | 13 |
| Table S14 | SUCRA values and ranking probabilities for HbA1c in middle-aged and older adults with type 2 diabetes. | 13 |
| Table S15 | SUCRA values and ranking probabilities for FPG in middle-aged and older adults with type 2 diabetes. | 14 |
| Table S16 | SUCRA values and ranking probabilities for VO_2_peak in middle-aged and older adults with type 2 diabetes. | 14 |
| Table S17 | SUCRA values and ranking probabilities for SBP in middle-aged and older adults with type 2 diabetes. | 14 |
| Table S18 | SUCRA values and ranking probabilities for HR in middle-aged and older adults with type 2 diabetes. | 14 |
| Table S19 | Leave-one-out sensitivity analysis for HbA1c in middle-aged and older adults with type 2 diabetes. | 15-19 |
| Table S20 | Leave-one-out sensitivity analysis for FPG in middle-aged and older adults with type 2 diabetes. | 19-22 |
| Table S21 | Leave-one-out sensitivity analysis for VO_2_peak in middle-aged and older adults with type 2 diabetes. | 22-23 |
| Table S22 | Leave-one-out sensitivity analysis for SBP in middle-aged and older adults with type 2 diabetes. | 23-24 |
| Table S23 | Leave-one-out sensitivity analysis for HR in middle-aged and older adults with type 2 diabetes. | 24-25 |
| Table S24 | Meta-regression analysis of country for HbA1c in middle-aged and older adults with type 2 diabetes. | 25 |
| Table S25 | Meta-regression analysis of follow-up duration for HbA1c in middle-aged and older adults with type 2 diabetes. | 25-26 |
| Table S26 | Meta-regression analysis of mean age for HbA1c in middle-aged and older adults with type 2 diabetes. | 2 |
| Table S27 | Meta-regression analysis of country for FPG in middle-aged and older adults with type 2 diabetes. | 26-27 |
| Table S28 | Meta-regression analysis of follow-up duration for FPG in middle-aged and older adults with type 2 diabetes. | 27 |
| Table S29 | Meta-regression analysis of mean age for FPG in middle-aged and older adults with type 2 diabetes. | 27 |
| Table S30 | Meta-regression analysis of country for VO_2_peak in middle-aged and older adults with type 2 diabetes. | 27-28 |
| Table S31 | Meta-regression analysis of follow-up duration for VO_2_peak in middle-aged and older adults with type 2 diabetes. | 28 |
| Table S32 | Meta-regression analysis of mean age for VO_2_peak in middle-aged and older adults with type 2 diabetes. | 28 |
| Table S33 | Meta-regression analysis of country for SBP in middle-aged and older adults with type 2 diabetes. | 29 |
| Table S34 | Meta-regression analysis of follow-up duration for SBP in middle-aged and older adults with type 2 diabetes. | 29 |
| Table S35 | Meta-regression analysis of mean age for SBP in middle-aged and older adults with type 2 diabetes. | 29 |
| Table S36 | Meta-regression analysis of country for HR in middle-aged and older adults with type 2 diabetes. | 30 |
| Table S37 | Meta-regression analysis of follow-up duration for HR in middle-aged and older adults with type 2 diabetes. | 30 |
| Table S38 | Meta-regression analysis of mean age for HR in middle-aged and older adults with type 2 diabetes. | 30 |
| Figure S1 | Comparison-adjusted funnel plot for the HbA1c outcome in middle-aged and older adults with type 2 diabetes. | 31 |
| Figure S2 | Comparison-adjusted funnel plot for the FPG outcome in middle-aged and older adults with type 2 diabetes. | 31 |
| Figure S3 | Comparison-adjusted funnel plot for the VO_2_peak outcome in middle-aged and older adults with type 2 diabetes. | 32 |
| Figure S4 | Comparison-adjusted funnel plot for the SBP outcome in middle-aged and older adults with type 2 diabetes. | 32 |
| Figure S5 | Comparison-adjusted funnel plot for the HR outcome in middle-aged and older adults with type 2 diabetes. | 33 |
| Table S39 | GRADE certainty of evidence for the HbA1c outcome in middle-aged and older adults with type 2 diabetes. | 33-36 |
| Table S40 | GRADE certainty of evidence for the FPG outcome in middle-aged and older adults with type 2 diabetes. | 36-38 |
| Table S41 | GRADE certainty of evidence for the VO_2_peak outcome in middle-aged and older adults with type 2 diabetes. | 38-39 |
| Table S42 | GRADE certainty of evidence for the SBP outcome in middle-aged and older adults with type 2 diabetes. | 39-41 |
| Table S43 | GRADE certainty of evidence for the HR outcome in middle-aged and older adults with type 2 diabetes. | 41 |
| Figure S6 | Subgroup analyses of HbA1c and FPG outcomes in middle-aged and older adults with type 2 diabetes | 42 |
| Table S44 | Meta-regression analysis of baseline HbA1c for HbA1c in middle-aged and older adults with type 2 diabetes | 42 |
| Table S45 | Meta-regression analysis of baseline HbA1c for FPG in middle-aged and older adults with type 2 diabetes | 43 |
| Table S46 | Meta-regression analysis of baseline HbA1c for VO2peak in middle-aged and older adults with type 2 diabetes | 43 |
| Table S47 | Meta-regression analysis of baseline HbA1c for SBP in middle-aged and older adults with type 2 diabetes | 44 |
| Table S48 | Meta-regression analysis of baseline HbA1c for HR in middle-aged and older adults with type 2 diabetes | 44 |
| Table S49 | Meta-regression analysis of session duration for HbA1c in middle-aged and older adults with type 2 diabetes | 44-45 |
| Table S50 | Meta-regression analysis of session duration for FPG in middle-aged and older adults with type 2 diabetes | 45 |
| Table S51 | Meta-regression analysis of session duration for VO2peak in middle-aged and older adults with type 2 diabetes | 45 |
| Table S52 | Meta-regression analysis of session duration for SBP in middle-aged and older adults with type 2 diabetes | 45-46 |
| Table S53 | Meta-regression analysis of session duration for HR in middle-aged and older adults with type 2 diabetes | 46 |
| Table S54 | Meta-regression analysis of frequency for HbA1c in middle-aged and older adults with type 2 diabetes | 46 |
| Table S55 | Meta-regression analysis of frequency for FPG in middle-aged and older adults with type 2 diabetes | 46-47 |
| Table S56 | Meta-regression analysis of frequency for VO2peak in middle-aged and older adults with type 2 diabetes | 47 |
| Table S57 | Meta-regression analysis of frequency for SBP in middle-aged and older adults with type 2 diabetes | 47 |
| Table S58 | Meta-regression analysis of frequency for HR in middle-aged and older adults with type 2 diabetes | 47 |
| Table S59 | Sensitivity analysis among middle-aged and older adults with type 2 diabetes mellitus not using R values | 48 |
| Table S60 | Baseline classification of exercise interventions and assigned network nodes in middle-aged and older adults with type 2 diabetes | 49-65 |

Table S1 PRISMA NMA Checklist of Items to Include When Reporting a Systematic Review Involving a Network Meta-analysis

| Section/Topic | Item # | Checklist Item | Reported on Page # |
| --- | --- | --- | --- |
| TITLE |  |  |  |
| Title | 1 | Identify the report as a systematic review *incorporating*  anetwork meta-analysis (or related form of meta-analysis). | 1 |
|  |  |  |  |
| ABSTRACT |  |  |  |
| Structured summary | 2 | Provide a structured summary including, as applicable:  **Background:** main objectives  **Methods:** data sources; study eligibility criteria, participants, and interventions; study appraisal; and *synthesis methods, such as network meta-analysis.*  **Results:** number of studies and participants identified; summary estimates with corresponding confidence/credible intervals; treatment rankings may also be discussed. Authors may choose to summarize pairwise comparisons against a chosen treatment included in their analyses for brevity.  **Discussion/Conclusions:** limitations; conclusions and implications of findings.  **Other:** systematic review registration number with registry name. | 1-2 |
|  |  |  |  |
| INTRODUCTION |  |  |  |
| Rationale | 3 | Describe the rationale for the review in the context of what is already known*, including mention of why a network meta-analysis has been conducted.* | 2 |
| Objectives | 4 | Provide an explicit statement of questions being addressed, with reference to participants, interventions, comparisons, outcomes, and study design (PICOS). | 3 |
|  |  |  |  |
| METHODS |  |  |  |
| Protocol and registration | 5 | Indicate whether a review protocol exists and if and where it can be accessed (e.g., Web address); and, if available, provide registration information, including registration number. | 3 |
| Eligibility criteria | 6 | Specify study characteristics (e.g., PICOS, length of follow-up) and report characteristics (e.g., years considered, language, publication status) used as criteria for eligibility, giving rationale. *Clearly describe eligible treatments included in the treatment network, and note whether any have been clustered or merged into the same node (with justification).* | 3-4 |
| Information sources | 7 | Describe all information sources (e.g., databases with dates of coverage, contact with study authors to identify additional studies) in the search and date last searched. | 4 |
| Search | 8 | Present full electronic search strategy for at least one database, including any limits used, such that it could be repeated. | 3，Supplementary  TableS2 |
| Study selection | 9 | State the process for selecting studies (i.e., screening, eligibility, included in systematic review, and, if applicable, included in the meta-analysis). | 3-4，Table 1 |
| Data collection process | 10 | Describe method of data extraction from reports (e.g., piloted forms, independently, in duplicate) and any processes for obtaining and confirming data from investigators. | 4 |
| Data items | 11 | List and define all variables for which data were sought (e.g., PICOS, funding sources) and any assumptions and simplifications made. | 3-4 |
| Geometry of the network | S1 | Describe methods used to explore the geometry of the treatment network under study and potential biases related to it. This should include how the evidence base has been graphically summarized for presentation, and what characteristics were compiled and used to describe the evidence base to readers. | 4 |
| Risk of bias within individual studies | 12 | Describe methods used for assessing risk of bias of individual studies (including specification of whether this was done at the study or outcome level), and how this information is to be used in any data synthesis. | 5 |
| Summary measures | 13 | State the principal summary measures (e.g., risk ratio, difference in means). Also describe the use of additional summary measures assessed, such as treatment rankings and surface under the cumulative ranking curve (SUCRA) values, as well as modified approaches used to present summary findings from meta-analyses. | 5 |
| Planned methods of analysis | 14 | Describe the methods of handling data and combining results of studies for each network meta-analysis. This should include, but not be limited to:   - Handling of multi-arm trials; - Selection of variance structure; - Selection of prior distributions in Bayesian analyses; and - Assessment of model fit. | 5 |
| Assessment of Inconsistency | S2 | Describe the statistical methods used to evaluate the agreement of direct and indirect evidence in the treatment network(s) studied. Describe efforts taken to address its presence when found. | 5 |
| Risk of bias across studies | 15 | Specify any assessment of risk of bias that may affect the cumulative evidence (e.g., publication bias, selective reporting within studies). | 5 |
| Additional analyses | 16 | Describe methods of additional analyses if done, indicating which were pre-specified. This may include, but not be limited to, the following:   - Sensitivity or subgroup analyses; - Meta-regression analyses; - Alternative formulations of the treatment network; and - Use of alternative prior distributions for Bayesian analyses (if applicable). | 5 |
| RESULTS† |  |  |  |
| Study selection | 17 | Give numbers of studies screened, assessed for eligibility, and included in the review, with reasons for exclusions at each stage, ideally with a flow diagram. | 6-8，Fig1 |
| Presentation of network structure | S3 | Provide a network graph of the included studies to enable visualization of the geometry of the treatment network. | 11，14，Fig3and 6 |
| Summary of network geometry | S4 | Provide a brief overview of characteristics of the treatment network. This may include commentary on the abundance of trials and randomized patients for the different interventions and pairwise comparisons in the network, gaps of evidence in the treatment network, and potential biases reflected by the network structure. | 10-13 |
| Study characteristics | 18 | For each study, present characteristics for which data were extracted (e.g., study size, PICOS, follow-up period) and provide the citations. | 5-7,Table 1 |
| Risk of bias within studies | 19 | Present data on risk of bias of each study and, if available, any outcome level assessment. | 10,Figure 2 |
| Results of individual studies | 20 | For all outcomes considered (benefits or harms), present, for each study: 1) simple summary data for each intervention group, and 2) effect estimates and confidence intervals. *Modified approaches may be needed to deal with information from larger networks.* | 10-16 |
| Synthesis of results | 21 | Present results of each meta-analysis done, including confidence/credible intervals. In larger networks, authors may focus on comparisons versus a particular comparator (e.g. placebo or standard care), with full findings presented in an appendix. League tables and forest plots may be considered to summarize pairwise comparisons. If additional summary measures were explored (such as treatment rankings), these should also be presented. | 10-16 |
| Exploration for inconsistency | S5 | Describe results from investigations of inconsistency. This may include such information as measures of model fit to compare consistency and inconsistency models, *P* values from statistical tests, or summary of inconsistency estimates from different parts of the treatment network. | 17 |
| Risk of bias across studies | 22 | Present results of any assessment of risk of bias across studies for the evidence base being studied. | 16,Supplementary Figure S7-12 |
| Results of additional analyses | 23 | Give results of additional analyses, if done (e.g., sensitivity or subgroup analyses, meta-regression analyses*, alternative network geometries studied, alternative choice of prior distributions for Bayesian analyses,* and so forth). | 17 |
|  |  |  |  |
| DISCUSSION |  |  |  |
| Summary of evidence | 24 | Summarize the main findings, including the strength of evidence for each main outcome; consider their relevance to key groups (e.g., healthcare providers, users, and policy-makers). | 17-18 |
| Limitations | 25 | Discuss limitations at study and outcome level (e.g., risk of bias), and at review level (e.g., incomplete retrieval of identified research, reporting bias). *Comment on the validity of the assumptions, such as transitivity and consistency. Comment on any concerns regarding network geometry (e.g., avoidance of certain comparisons).* | 19-20 |
| Conclusions | 26 | Provide a general interpretation of the results in the context of other evidence, and implications for future research. | 21 |
|  |  |  |  |
| FUNDING |  |  |  |
| Funding | 27 | Describe sources of funding for the systematic review and other support (e.g., supply of data); role of funders for the systematic review. This should also include information regarding whether funding has been received from manufacturers of treatments in the network and/or whether some of the authors are content experts with professional conflicts of interest that could affect use of treatments in the network. | 22 |

PICOS = population, intervention, comparators, outcomes, study design.

* Text in italics indicate S wording specific to reporting of network meta-analyses that has been added to guidance from the PRISMA statement.

† Authors may wish to plan for use of appendices to present all relevant information in full detail for items in this section.

| **Table S2.Literature Search Strategy** | |
| --- | --- |
| Pubmed | ((((((((((((((((((((((((((((((((((("Diabetes Mellitus, Type 2"[Mesh]) OR (Diabetes Mellitus, Type 2[Title/Abstract])) OR (Diabetes Mellitus, Adult-Onset[Title/Abstract])) OR (Adult-Onset Diabetes Mellitus[Title/Abstract])) OR (Diabetes Mellitus, Adult Onset[Title/Abstract])) OR (Diabetes Mellitus, Ketosis-Resistant[Title/Abstract])) OR (Diabetes Mellitus, Ketosis Resistant[Title/Abstract])) OR (Ketosis-Resistant Diabetes Mellitus[Title/Abstract])) OR (NIDDM[Title/Abstract])) OR (Diabetes Mellitus, Maturity-Onset[Title/Abstract])) OR (Diabetes Mellitus, Maturity Onset[Title/Abstract])) OR (Diabetes Mellitus, Non Insulin Dependent[Title/Abstract])) OR (Diabetes Mellitus, Non-Insulin-Dependent[Title/Abstract])) OR (Non-Insulin-Dependent Diabetes Mellitus[Title/Abstract])) OR (Diabetes Mellitus, Noninsulin Dependent[Title/Abstract])) OR (Diabetes Mellitus, Noninsulin-Dependent[Title/Abstract])) OR (Diabetes Mellitus, Slow-Onset[Title/Abstract])) OR (Diabetes Mellitus, Slow Onset[Title/Abstract])) OR (Slow-Onset Diabetes Mellitus[Title/Abstract])) OR (Diabetes Mellitus, Stable[Title/Abstract])) OR (Stable Diabetes Mellitus[Title/Abstract])) OR (Diabetes Mellitus, Type II[Title/Abstract])) OR (Maturity-Onset Diabetes[Title/Abstract])) OR (Diabetes, Maturity-Onset[Title/Abstract])) OR (Maturity Onset Diabetes[Title/Abstract])) OR (Maturity-Onset Diabetes Mellitus[Title/Abstract])) OR (Maturity Onset Diabetes Mellitus[Title/Abstract])) OR (MODY (Maturity-onset diabetes of the young[Title/Abstract]))) OR (Noninsulin-Dependent Diabetes Mellitus[Title/Abstract])) OR (Noninsulin Dependent Diabetes Mellitus[Title/Abstract])) OR (Type 2 Diabetes[Title/Abstract])) OR (Diabetes, Type 2[Title/Abstract])) OR (Type 2 Diabetes Mellitus[Title/Abstract])) AND ((("Aged"[Mesh]) OR (Elderly[Title/Abstract])) OR (Aged[Title/Abstract]))) AND (((((((((((((((((((((((((("Resistance Training"[Mesh]) OR (Resistance Training[Title/Abstract])) OR (Training, Resistance[Title/Abstract])) OR (Strength Training[Title/Abstract])) OR (Training, Strength[Title/Abstract])) OR (Weight-Lifting Strengthening Program[Title/Abstract])) OR (Strengthening Programs, Weight-Lifting[Title/Abstract])) OR (Strengthening Program, Weight-Lifting[Title/Abstract])) OR (Weight Lifting Strengthening Program[Title/Abstract])) OR (Weight-Lifting Strengthening Programs[Title/Abstract])) OR (Weight-Lifting Exercise Program[Title/Abstract])) OR (Exercise Programs, Weight-Lifting[Title/Abstract])) OR (Exercise Program, Weight-Lifting[Title/Abstract])) OR (Weight Lifting Exercise Program[Title/Abstract])) OR (Weight-Lifting Exercise Programs[Title/Abstract])) OR (Weight-Bearing Strengthening Program[Title/Abstract])) OR (Strengthening Programs, Weight-Bearing[Title/Abstract])) OR (Strengthening Program, Weight-Bearing[Title/Abstract])) OR (Weight Bearing Strengthening Program[Title/Abstract])) OR (Weight-Bearing Strengthening Programs[Title/Abstract])) OR (Weight-Bearing Exercise Program[Title/Abstract])) OR (Exercise Programs, Weight-Bearing[Title/Abstract])) OR (Exercise Program, Weight-Bearing[Title/Abstract])) OR (Weight Bearing Exercise Program[Title/Abstract])) OR (Weight-Bearing Exercise Programs[Title/Abstract])) OR ((((((((((((((((((((((((((((("Exercise"[Mesh]) OR (Exercise[Title/Abstract])) OR (Exercises[Title/Abstract])) OR (Exercise, Physical[Title/Abstract])) OR (Exercises, Physical[Title/Abstract])) OR (Physical Exercise[Title/Abstract])) OR (Physical Exercises[Title/Abstract])) OR (Exercise, Isometric[Title/Abstract])) OR (Exercises, Isometric[Title/Abstract])) OR (Isometric Exercises[Title/Abstract])) OR (Isometric Exercise[Title/Abstract])) OR (Exercise, Aerobic[Title/Abstract])) OR (Aerobic Exercise[Title/Abstract])) OR (Aerobic Exercises[Title/Abstract])) OR (Exercises, Aerobic[Title/Abstract])) OR (Exercise Training[Title/Abstract])) OR (Exercise Trainings[Title/Abstract])) OR (Training, Exercise[Title/Abstract])) OR (Trainings, Exercise[Title/Abstract])) OR (Physical Activity[Title/Abstract])) OR (Activities, Physical[Title/Abstract])) OR (Activity, Physical[Title/Abstract])) OR (Physical Activities[Title/Abstract])) OR (Active Breaks[Title/Abstract])) OR (Activity Breaks[Title/Abstract])) OR (Acute Exercise[Title/Abstract])) OR (Acute Exercises[Title/Abstract])) OR (Exercise, Acute[Title/Abstract])) OR (Exercises, Acute[Title/Abstract])))) |
|  |  |
|  |  |
|  |  |
|  |  |
|  |  |
|  |  |
| Web of Science | 1: TS=("Diabetes Mellitus Type 2" OR "Diabetes Mellitus, Type 2" OR "Diabetes Mellitus,  Adult-Onset" OR "Adult-Onset Diabetes Mellitus" OR "Diabetes Mellitus, Adult Onset" OR  "Diabetes Mellitus, Ketosis-Resistant" OR "Diabetes Mellitus, Ketosis Resistant" OR  "Ketosis-Resistant Diabetes Mellitus" OR NIDDM OR "Diabetes Mellitus, Maturity-Onset" OR  "Diabetes Mellitus, Maturity Onset" OR "Diabetes Mellitus, Non Insulin Dependent" OR  "Diabetes Mellitus, Non-Insulin-Dependent" OR "Non-Insulin-Dependent Diabetes Mellitus" OR  "Diabetes Mellitus, Noninsulin Dependent" OR "Diabetes Mellitus, Noninsulin-Dependent" OR  "Diabetes Mellitus, Slow-Onset" OR "Diabetes Mellitus, Slow Onset" OR "Slow-Onset Diabetes  Mellitus" OR "Diabetes Mellitus, Stable" OR "Stable Diabetes Mellitus" OR "Diabetes Mellitus,  Type II" OR "Maturity-Onset Diabetes" OR "Diabetes, Maturity-Onset" OR "Maturity Onset  Diabetes" OR "Maturity-Onset Diabetes Mellitus" OR "Maturity Onset Diabetes Mellitus" OR  "Maturity-onset diabetes of the young" OR MODY OR "Noninsulin-Dependent Diabetes Mellitus"  OR "Noninsulin Dependent Diabetes Mellitus" OR "Type 2 Diabetes" OR "Diabetes, Type 2" )  2: TS=("aged" OR elderly)  3:#1AND#2  4: TS=("resistance training" OR "training resistance" OR "strength training" OR "trainingstrength" OR "weight lifting strengthening program*" OR "weight-lifting strengthening program*"  OR "weight lifting exercise program*" OR "weight-lifting exercise program*" OR "weight bearing  strengthening program*" OR "weight-bearing strengthening program*" OR "weight bearing  exercise program*" OR "weight-bearing exercise program*" OR exercise OR exercises OR  "physical exercise" OR "exercise physical" OR "exercise training" OR "training exercise" OR  "physical activity" OR "activity physical" OR "active break*" OR "activity break*" OR "acute  exercise" OR "aerobic exercise*" OR "isometric exercise*")  5: TS=(random* OR randomi* OR randomly OR placebo* OR sham OR trial OR "clinical trial"  OR "controlled clinical trial" OR "double blind" OR "single blind" OR "triple blind" OR crossover  OR "cross over" OR "parallel group" OR cluster random* OR RCT)  6:#3AND#4AND#5 |
|  |  |
|  |  |
|  |  |
|  |  |
|  |  |
|  |  |
|  |  |
| Cochrane | #1 MeSH descriptor: [Diabetes Mellitus, Type 2] explode all trees  #2 (Diabetes Mellitus, Type 2):ti,ab,kw OR ("Maturity-Onset Diabetes Mellitus" OR "Diabetes Mellitus, Slow-Onset" OR "Type 2 Diabetes" OR "Adult-Onset Diabetes Mellitus" OR "Ketosis-Resistant Diabetes Mellitus" OR "Diabetes Mellitus, Type II" OR "Slow-Onset Diabetes Mellitus" OR "Type 2 Diabetes Mellitus" OR "Diabetes Mellitus, Noninsulin-Dependent" OR "Maturity Onset Diabetes" OR "Diabetes Mellitus, Non Insulin Dependent" OR "Diabetes Mellitus, Slow Onset" OR "Diabetes Mellitus, Noninsulin Dependent" OR "Non-Insulin-Dependent Diabetes Mellitus" OR "Diabetes Mellitus, Ketosis-Resistant" OR "Maturity-Onset Diabetes" OR "Diabetes Mellitus, Stable" OR "Diabetes Mellitus, Ketosis Resistant" OR "Diabetes Mellitus, Maturity Onset" OR "Noninsulin Dependent Diabetes Mellitus" OR "Diabetes Mellitus, Adult Onset" OR NIDDM OR "Diabetes Mellitus, Adult-Onset" OR "Diabetes Mellitus, Maturity-Onset" OR "Maturity Onset Diabetes Mellitus" OR "Diabetes, Type 2" OR "Diabetes, Maturity-Onset" OR MODY OR "Noninsulin-Dependent Diabetes Mellitus" OR "Diabetes Mellitus, Non-Insulin-Dependent" OR "Stable Diabetes Mellitus"):ti,ab,kw  #3 #1OR#2  #4 MeSH descriptor: [Aged] explode all trees  #5 ("aged"):ti,ab,kw OR ("elderly"):ti,ab,kw  #6 #4OR#5  #7 #3AND#6  #8 MeSH descriptor: [Exercise] explode all trees  #9 ("exercise"):ti,ab,kw OR ("Physical Activity" OR "Physical Activities" OR "Activity, Physical" OR "Activities, Physical" OR "Isometric Exercise" OR "Exercise, Isometric" OR "Exercises, Isometric" OR "Isometric Exercises" OR "Exercises, Physical" OR "Exercises" OR "Physical Exercises" OR "Exercise, Physical" OR "Physical Exercise" OR "Exercises, Acute" OR "Exercise, Acute" OR "Acute Exercises" OR "Acute Exercise" OR "Aerobic Exercises" OR "Exercise, Aerobic" OR "Exercises, Aerobic" OR "Aerobic Exercise" OR "Training, Exercise" OR "Trainings, Exercise" OR "Exercise Trainings" OR "Exercise Training"):ti,ab,kw  #10 #8OR#9  #11 MeSH descriptor: [Resistance Training] explode all trees  #12 (Resistance Training):ti,ab,kw OR ("Training, Resistance" OR "Strength Training" OR "Training, Strength" OR "Weight-Bearing Exercise Program" OR "Weight Bearing Exercise Program" OR "Weight-Bearing Strengthening Program" OR "Weight-Bearing Exercise Programs" OR "Exercise Program, Weight-Bearing" OR "Strengthening Program, Weight-Bearing" OR "Strengthening Programs, Weight-Bearing" OR "Weight Bearing Strengthening Program" OR "Exercise Programs, Weight-Bearing" OR "Weight-Bearing Strengthening Programs" OR "Weight-Lifting Exercise Programs" OR "Weight Lifting Exercise Program" OR "Exercise Program, Weight-Lifting" OR "Weight-Lifting Exercise Program" OR "Weight Lifting Strengthening Program" OR "Weight-Lifting Strengthening Program" OR "Strengthening Programs, Weight-Lifting" OR "Weight-Lifting Strengthening Programs" OR "Exercise Programs, Weight-Lifting" OR "Strengthening Program, Weight-Lifting"):ti,ab,kw  #13 #11OR#12  #14 #13OR#10  #15 #7AND#14 |
|  |  |
|  |  |
|  |  |
| Embase | #1 'non insulin dependent diabetes mellitus'/exp  #2 (((('adult onset diabetes':ti,ab,kw OR 'adult onset diabetes mellitus':ti,ab,kw OR 'diabetes mellitus type 2':ti,ab,kw OR 'diabetes mellitus type ii':ti,ab,kw OR 'diabetes mellitus, maturity onset':ti,ab,kw OR 'diabetes mellitus, non insulin dependent':ti,ab,kw OR 'diabetes mellitus, non-insulin-dependent':ti,ab,kw OR 'diabetes mellitus, type 2':ti,ab,kw OR 'diabetes mellitus, type ii':ti,ab,kw OR 'diabetes type 2':ti,ab,kw OR 'diabetes type ii':ti,ab,kw OR 'diabetes, adult onset':ti,ab,kw OR 'dm 2':ti,ab,kw OR 'insulin independent diabetes':ti,ab,kw OR 'insulin independent diabetes mellitus':ti,ab,kw OR 'ketosis resistant diabetes mellitus':ti,ab,kw OR 'maturity onset diabetes':ti,ab,kw OR 'maturity onset diabetes mellitus':ti,ab,kw OR 'niddm':ti,ab,kw OR niddm:ti,ab,kw) AND 'non insulin dependent diabetes mellitus':ti,ab,kw OR 'non insulin dependent':ti,ab,kw) AND 'type 2':ti,ab,kw AND 'diabetes mellitus':ti,ab,kw OR 'non insulin dependent diabetes':ti,ab,kw OR 'non-insulin-dependent diabetes mellitus':ti,ab,kw OR 'noninsulin dependent':ti,ab,kw) AND 'type 2':ti,ab,kw AND 'diabetes mellitus':ti,ab,kw OR 'noninsulin dependent diabetes':ti,ab,kw OR 'noninsulin dependent diabetes mellitus':ti,ab,kw OR 't2dm':ti,ab,kw OR 'tiidm':ti,ab,kw OR 'type 2':ti,ab,kw) AND 'insulin independent':ti,ab,kw AND diabetes:ti,ab,kw OR 'type 2 diabetes':ti,ab,kw OR 'type 2 diabetes mellitus':ti,ab,kw OR 'type ii diabetes':ti,ab,kw OR 'type ii diabetes mellitus':ti,ab,kw OR 'non insulin dependent diabetes mellitus':ti,ab,kw  #3 #1 OR #2  #4 'aged'/exp  #5 'aged patient':ti,ab,kw OR 'aged people':ti,ab,kw OR 'aged person':ti,ab,kw OR 'aged subject':ti,ab,kw OR 'elderly':ti,ab,kw OR 'elderly patient':ti,ab,kw OR 'elderly people':ti,ab,kw OR 'elderly person':ti,ab,kw OR 'elderly subject':ti,ab,kw OR 'senior citizen':ti,ab,kw OR 'senium':ti,ab,kw OR 'aged':ti,ab,kw  #6 #4 OR #5  #7 'aerobic exercise'/exp  #8 'aerobic dance':ti,ab,kw OR 'aerobic dancing':ti,ab,kw OR 'aerobics':ti,ab,kw OR 'aerobics exercise':ti,ab,kw OR 'dancing, aerobic':ti,ab,kw OR 'exercise, aerobic':ti,ab,kw OR 'low impact aerobic exercise':ti,ab,kw OR 'low impact aerobics':ti,ab,kw OR 'step aerobics':ti,ab,kw OR 'aerobic exercise':ti,ab,kw  #9 #7 OR #8  #10 'resistance training'/exp  #11 'resistance exercise':ti,ab,kw OR 'resistance exercise training':ti,ab,kw OR 'resistance-type exercise':ti,ab,kw OR 'resistance-type training':ti,ab,kw OR 'strength training':ti,ab,kw OR 'strength-type exercise':ti,ab,kw OR 'strength-type training':ti,ab,kw OR 'resistance training':ti,ab,kw  #12 #10 OR #11  #13 #9 OR #12  #14 #3 AND #6 AND #13  #15 #14 AND 'randomized controlled trial'/de |
|  |  |
|  |  |
|  |  |
|  |  |
|  |  |
|  |  |
|  |  |
|  |  |
|  |  |
|  |  |

Supplementary Table3.Global inconsistency assessment for HbA1c, FPG, VO₂peak, SBP, and resting HR in middle-aged and older adults with type 2 diabetes.

| **结局指标名称** | **全局不一致性分析的P值** |
| --- | --- |
| HbA1c | 0.8048 |
| FPG | 0.7123 |
| VO_2_peak | 0.5263 |
| SBP | 0.2391 |
| HR | 0.2770 |

Supplementary Table4.Node-splitting analysis for HbA1c in middle-aged and older adults with type 2 diabetes.

| **Side** | **Direct** |  | **Indirect** |  | **Difference** |  |  | **tau** |
| --- | --- | --- | --- | --- | --- | --- | --- | --- |
|  | **Coef.** | **Std.Err.** | **Coef.** | **Std.Err.** | **Coef.** | **Std. Err.** | **P>z** |  |
| A E | -0.53 | 0.4512468 | -0.8626268 | 0.3438194 | 0.3326268 | 0.5673055 | 0.558 | 0.3518067 |
| A H | -0.2533125 | 0.2134471 | 0.0822475 | 0.5238115 | -0.3355601 | 0.5657963 | 0.553 | 0.3517069 |
| B D | -0.1073386 | 0.3311054 | -0.9679319 | 1.097666 | 0.8605933 | 1.139653 | 0.45 | 0.3486818 |
| B H | 0.1934065 | 0.3653514 | 0.6065802 | 0.7197633 | -0.4131737 | 0.8299918 | 0.619 | 0.3589587 |
| B I | 0.3602162 | 0.315857 | 0.3841583 | 0.6815201 | -0.0239422 | 0.7291392 | 0.974 | 0.3555822 |
| C I | 0.3 | 0.3721136 | -0.2148682 | 54.88073 | 0.5148682 | 54.88203 | 0.993 | 0.3465495 |
| D H | 0.2924393 | 0.3541443 | 0.7773894 | 0.4943286 | -0.4849501 | 0.6223997 | 0.436 | 0.3556524 |
| D I | 0.5772554 | 0.2600757 | 0.1635517 | 0.7951215 | 0.4137037 | 0.8299961 | 0.618 | 0.3589646 |
| E I | 0.6448173 | 0.1705897 | 0.3106458 | 0.540709 | 0.3341715 | 0.5674041 | 0.556 | 0.3517954 |
| F G | -0.1930698 | 0.3397403 | 0.4660786 | 0.6043786 | -0.6591484 | 0.6968052 | 0.344 | 0.3460415 |
| F I | 0.5531271 | 0.2400666 | 0.2470194 | 1.256416 | 0.3061077 | 1.274735 | 0.81 | 0.3548432 |
| G I | 0.4878598 | 0.2778382 | 1.504585 | 0.8898998 | -1.016725 | 0.9305831 | 0.275 | 0.343915 |
| H I | 0.0076863 | 0.2075193 | 0.464301 | 0.4735016 | -0.4566147 | 0.5125279 | 0.373 | 0.3468586 |

Supplementary Table5.Node-splitting analysis for FPG in middle-aged and older adults with type 2 diabetes.

| **Side** | **Direct** |  | **Indirect** |  | **Difference** |  |  | **tau** |
| --- | --- | --- | --- | --- | --- | --- | --- | --- |
|  | Coef. | Std. Err. | Coef. | Std. Err. | Coef. | Std. Err. | P>z |  |
| A B | -14.03872 | 12.7176 | -8.982528 | 20.23334 | -5.056193 | 23.89896 | 0.832 | 16.53103 |
| A D | -14.4 | 23.46807 | 4.468842 | 18.50436 | -18.86884 | 29.88582 | 0.528 | 16.11121 |
| A G | -11.98024 | 12.89628 | -31.24199 | 21.46705 | 19.26175 | 25.04261 | 0.442 | 15.97472 |
| B C | 2.749859 | 16.2137 | 1.982106 | 33.3196 | 0.7677525 | 37.11331 | 0.983 | 16.44861 |
| B G | -12.12031 | 19.75681 | 1.902816 | 18.1676 | -14.02312 | 26.82267 | 0.601 | 16.31814 |
| B H | 24.88606 | 15.76301 | 24.08281 | 24.29742 | 0.8032483 | 28.96906 | 0.978 | 16.51009 |
| C G | -15.43049 | 19.28241 | 14.6335 | 31.96078 | -30.064 | 38.35748 | 0.433 | 15.96738 |
| C H | 23.89641 | 13.43713 | -13.91595 | 58.14782 | 37.81236 | 59.48661 | 0.525 | 16.10623 |
| D H | 13.2373 | 8.656645 | 32.12489 | 28.60874 | -18.88759 | 29.89198 | 0.527 | 16.11155 |
| E F | -18.71615 | 19.7408 | -8.150068 | 27.52007 | -10.56608 | 33.99598 | 0.756 | 16.54042 |
| E H | 2.15504 | 12.62952 | 42.10277 | 53.54796 | -39.94773 | 54.52081 | 0.464 | 16.12553 |
| F H | 14.14221 | 13.24703 | 71.16191 | 45.25388 | -57.0197 | 47.22337 | 0.227 | 15.11236 |
| G H | 36.67535 | 18.76818 | 17.12984 | 23.77569 | 19.54551 | 30.4552 | 0.521 | 15.93189 |

Supplementary Table6.Node-splitting analysis for VO_2_peak in middle-aged and older adults with type 2 diabetes.

| **Side** | **Direct** |  | **Indirect** |  | **Difference** |  |  | **tau** |
| --- | --- | --- | --- | --- | --- | --- | --- | --- |
|  | Coef. | Std. Err. | Coef. | Std. Err. | Coef. | Std. Err. | P>z |  |
| A F | 0.46 | 1.382319 | -0.4445747 | 33.66378 | 0.9045747 | 33.69215 | 0.979 | 1.20e-08 |
| B D | -0.8302364 | 1.109866 | -3.184575 | 2.09843 | 2.354339 | 2.371629 | 0.321 | 1.91e-09 |
| B E | -3.616724 | 2.103765 | -2.074041 | 1.720352 | -1.542683 | 2.71942 | 0.571 | 7.95e-08 |
| B F | -1.621364 | 1.049391 | -6.849602 | 3.7489 | 5.228238 | 3.93399 | 0.184 | 1.12e-09 |
| B G | -3.293877 | 0.9614942 | -1.760339 | 2.601661 | -1.533538 | 2.704417 | 0.571 | 1.55e-07 |
| C E | -3.3 | 0.8833378 | -0.9183978 | 91.48169 | -2.381602 | 91.48277 | 0.979 | 3.75e-10 |
| D F | -0.7988668 | 1.034047 | 0.1928541 | 3.316485 | -0.9917209 | 3.504386 | 0.777 | 2.60e-09 |
| D G | -1.482079 | 0.892525 | -6.73737 | 3.803264 | 5.255291 | 3.940031 | 0.182 | 1.21e-09 |
| E G | -0.5599209 | 1.016885 | 4.674608 | 3.977316 | -5.234529 | 3.930587 | 0.183 | 1.11e-09 |
| F G | -0.6666446 | 1.140998 | -2.944832 | 2.611539 | 2.278187 | 2.968114 | 0.443 | 5.90e-10 |

Supplementary Table7.Node-splitting analysis for SBP in middle-aged and older adults with type 2 diabetes.

| **Side** | **Direct** |  | **Indirect** |  | **Difference** |  |  | **tau** |
| --- | --- | --- | --- | --- | --- | --- | --- | --- |
|  | Coef. | Std. Err. | Coef. | Std. Err. | Coef. | Std. Err. | P>z |  |
| A B | -9.508282 | 0.7970897 | -9.016828 | 7.916197 | -0.4914535 | 7.956226 | 0.951 | 4.79e-10 |
| A F | -5 | 4.069817 | -5.481793 | 6.843786 | 0.4817928 | 7.962463 | 0.952 | 2.30e-09 |
| B C | 2.5 | 6.465109 | 20.75665 | 11.85815 | -18.25665 | 13.44294 | 0.174 | 5.78e-09 |
| B D | 3.997468 | 5.179697 | 2.700744 | 5.681669 | 1.296724 | 7.214482 | 0.857 | 6.24e-09 |
| B F | -0.9656634 | 7.189434 | 5.99009 | 4.012465 | -6.955753 | 8.145895 | 0.393 | 6.74e-09 |
| B G | 8.148596 | 4.348736 | 3.271058 | 7.716237 | 4.877538 | 8.450646 | 0.564 | 4.77e-09 |
| C F | -2.5 | 7.280453 | -1.536414 | 13.41209 | -0.9635856 | 15.92493 | 0.952 | 3.89e-09 |
| C G | -4.1 | 6.691773 | 20.93782 | 14.43969 | -25.03783 | 16.04831 | 0.119 | 2.02e-08 |
| D G | 4.079811 | 1.813878 | -20.95476 | 15.89362 | 25.03458 | 16.04797 | 0.119 | 8.13e-09 |
| E G | 4 | 5.881174 | -4.812641 | 1018.744 | 8.812641 | 1018.76 | 0.993 | 4.13e-09 |
| F G | -2.435458 | 7.400169 | 6.796971 | 6.574301 | -9.23243 | 9.941368 | 0.353 | 3.90e-08 |

Supplementary Table8.Node-splitting analysis for HR in middle-aged and older adults with type 2 diabetes.

| **Side** | **Direct** |  | **Indirect** |  | **Difference** |  |  | **tau** |
| --- | --- | --- | --- | --- | --- | --- | --- | --- |
|  | Coef. | Std. Err. | Coef. | Std. Err. | Coef. | Std. Err. | P>z |  |
| A B | -1.351564 | 1.990997 | -5.25647 | 70.05666 | 3.904906 | 70.08495 | 0.956 | 2.744707 |
| B C | 2.711255 | 4.628726 | 13.01982 | 8.326887 | -10.30856 | 9.583843 | 0.282 | 2.672165 |
| B E | 7.601493 | 4.360752 | -2.800579 | 8.7367 | 10.40207 | 9.575476 | 0.277 | 2.671771 |
| C E | 0.4910156 | 1.805209 | -7.107993 | 657.753 | 7.599008 | 657.7547 | 0.991 | 2.743496 |
| D E | 1 | 4.69087 | 8.598954 | 657.5455 | -7.598954 | 657.5652 | 0.991 | 2.743496 |

Supplementary Table9.Loop-specific inconsistency analysis for HbA1c in middle-aged and older adults with type 2 diabetes.

| **Loop** | **IF** | **seIF** | **z_value** | **p_value** | **CI_95** | **Loop_Heterog_tau2** |
| --- | --- | --- | --- | --- | --- | --- |
|  |  |  |  |  |  |  |
| LIRT-MIAT-UC | 0.511 | 0.508 | 1.007 | 0.314 | (0.00,1.51) | 0.085 |
| HIAT-HIAT-MIRT-UC | 0.455 | 0.536 | 0.849 | 0.396 | (0.00,1.51) | 0 |
| AC-HIRT-MIRT-UC | 0.34 | 0.606 | 0.561 | 0.575 | (0.00,1.53) | 0.151 |
| HIAT-MIRT-MIRT-UC | 0.202 | 0.355 | 0.57 | 0.569 | (0.00,0.90) | 0 |
| HIAT-MIRT-UC | 0.096 | 0.377 | 0.255 | 0.799 | (0.00,0.83) | 0 |
| HIAT-HIAT-MIRT-MIRT | . | . | . | . |  | 0 |

Supplementary Table10.Loop-specific inconsistency analysis for FPG in middle-aged and older adults with type 2 diabetes.

| **Loop** | **IF** | **seIF** | **z_value** | **p_value** | **CI_95** | **Loop_Heterog_tau2** |
| --- | --- | --- | --- | --- | --- | --- |
| LIRT-MIAT-UC | 28.314 | 13.005 | 2.177 | 0.029 | (2.82,53.80) | 0 |
| AC-HIRT-MIRT-UC | 28.238 | 36.776 | 0.768 | 0.443 | (0.00,100.32) | 288.632 |
| AC-HIAT-MIRT | 13.973 | 24.262 | 0.576 | 0.565 | (0.00,61.52) | 174.749 |
| AC-HIAT-HIRT-UC | 11.478 | 31.875 | 0.36 | 0.719 | (0.00,73.95) | 200.931 |
| HIAT-HIAT-MIRT-UC | 7.855 | 27.23 | 0.288 | 0.773 | (0.00,61.22) | 0 |
| HIAT-MIRT-MIRT-UC | 3.34 | 31.415 | 0.106 | 0.915 | (0.00,64.91) | 0 |
| HIAT-MIRT-UC | . | . | . | . |  | 791.937 |
| HIAT-HIAT-MIRT-MIRT | . | . | . | . |  | 0 |

Supplementary Table11.Loop-specific inconsistency analysis for VO_2_peak in middle-aged and older adults with type 2 diabetes.

| **Loop** | **IF** | **seIF** | **z_value** | **p_value** | **CI_95** | **Loop_Heterog_tau2** |
| --- | --- | --- | --- | --- | --- | --- |
|  |  |  |  |  |  |  |
| HIAT-MIRT-UC | 2.692 | 2.258 | 1.193 | 0.233 | (0.00,7.12) | 0 |
| HIAT-HIRT-UC | 1.82 | 2.637 | 0.69 | 0.49 | (0.00,6.99) | 0 |
| HIAT-HIAT-MIRT-UC | 1.735 | 1.969 | 0.881 | 0.378 | (0.00,5.60) | 0.023 |
| HIAT-MIRT-MIRT-UC | 0.117 | 2.061 | 0.057 | 0.955 | (0.00,4.16) | 0 |
| HIAT-HIAT-MIRT-MIRT | . | . | . | . |  | 0 |

Supplementary Table12.Loop-specific inconsistency analysis for SBP in middle-aged and older adults with type 2 diabetes.

| **Loop** | **IF** | **seIF** | **z_value** | **p_value** | **CI_95** | **Loop_Heterog_tau2** |
| --- | --- | --- | --- | --- | --- | --- |
|  |  |  |  |  |  |  |
| HIAT-MIRT-UC | 17.03 | 11.857 | 1.436 | 0.151 | (0.00,40.27) | 0 |
| HIAT-HIAT-MIRT-UC | 17.03 | 10.949 | 1.555 | 0.12 | (0.00,38.49) | 0 |
| AC-HIAT-MIRT | 4.508 | 8.328 | 0.541 | 0.588 | (0.00,20.83) | 0 |
| HIAT-HIRT-UC | 1.287 | 7.527 | 0.171 | 0.864 | (0.00,16.04) | 0 |
| HIAT-MIRT-MIRT-UC | . | . | . | . |  | 0 |
| HIAT-HIAT-MIRT-MIRT | . | . | . | . |  | 0 |

Supplementary Table13.Loop-specific inconsistency analysis for HR in middle-aged and older adults with type 2 diabetes.

|  | **Loop** | **IF** | **seIF** | **z_value** | **p_value** | **CI_95** | **Loop_Heterog_tau2** |  |
| --- | --- | --- | --- | --- | --- | --- | --- | --- |
| HIAT | HIRT-UC | 6.241 | 5.151 | 1.212 | 0.226 | (0.00,16.34) | 0 |  |

Supplementary Table14.SUCRA values and ranking probabilities for HbA1c in middle-aged and older adults with type 2 diabetes.

| **Treatment** | **SUCRA** | **PrBest** | **MeanRank** |
| --- | --- | --- | --- |
| HIRT | 78.6 | 22.2 | 2.7 |
| MIAT | 73.3 | 24 | 3.1 |
| HIAT-MIRT | 71.4 | 19.4 | 3.3 |
| LIRT | 70.4 | 15.5 | 3.4 |
| HIAT | 53.4 | 7.7 | 4.7 |
| HIAT-LIRT | 48.9 | 10.9 | 5.1 |
| MIRT | 27.2 | 0.1 | 6.8 |
| UC | 17.2 | 0 | 7.6 |
| AC | 9.6 | 0 | 8.2 |

Supplementary Table15.SUCRA values and ranking probabilities for FPG in middle-aged and older adults with type 2 diabetes.

| **Treatment** | **SUCRA** | **PrBest** | **MeanRank** |
| --- | --- | --- | --- |
| MIRT | 80.5 | 39.4 | 2.4 |
| HIAT | 72.5 | 19.6 | 2.9 |
| HIAT-MIRT | 65.1 | 17 | 3.4 |
| MIAT | 58.4 | 17.4 | 3.9 |
| HIRT | 49.1 | 4.3 | 4.6 |
| AC | 38.7 | 0.8 | 5.3 |
| LIRT | 24.6 | 1.6 | 6.3 |
| UC | 11.1 | 0 | 7.2 |

Supplementary Table16.SUCRA values and ranking probabilities for VO_2_peak in middle-aged and older adults with type 2 diabetes.

| **Treatment** | **SUCRA** | **PrBest** | **MeanRank** |
| --- | --- | --- | --- |
| HIAT-HIRT | 90.3 | 63.4 | 1.6 |
| HIAT | 85.9 | 31.7 | 1.8 |
| HIAT-MIRT | 58.8 | 1.9 | 3.5 |
| MIRT | 41.3 | 0.2 | 4.5 |
| AC | 32 | 2.8 | 5.1 |
| HIRT | 27.6 | 0 | 5.3 |
| UC | 14.2 | 0 | 6.2 |

Supplementary Table17.SUCRA values and ranking probabilities for SBP in middle-aged and older adults with type 2 diabetes.

| **Treatment** | **SUCRA** | **PrBest** | **MeanRank** |
| --- | --- | --- | --- |
| HIAT | 86.7 | 49 | 1.8 |
| HIRT | 64.7 | 9.1 | 3.1 |
| LIRT | 60.4 | 28.9 | 3.4 |
| MIRT | 54.2 | 5.6 | 3.8 |
| HIAT-MIRT | 38.6 | 7.4 | 4.7 |
| UC | 30.1 | 0 | 5.2 |
| AC | 15.3 | 0 | 6.1 |

Supplementary Table18.SUCRA values and ranking probabilities for HR in middle-aged and older adults with type 2 diabetes.

| **Treatm~t** | **SUCRA** | **PrBest** | **MeanRank** |
| --- | --- | --- | --- |
| HIAT | 83.2 | 53.8 | 1.7 |
| AC | 64.9 | 20.5 | 2.4 |
| LIRT | 41.8 | 20.3 | 3.3 |
| HIRT | 34 | 3.9 | 3.6 |
| UC | 26.2 | 1.6 | 4 |

Supplementary Table19.Leave-one-out sensitivity analysis for HbA1c in middle-aged and older adults with type 2 diabetes.

| **dropped_id** | **comparison** | **eff** | **lci** | **uci** | **connected** |
| --- | --- | --- | --- | --- | --- |
| [Cíntia E Botton](https://pubmed.ncbi.nlm.nih.gov/?term=Botton+CE&cauthor_id=30296453) | ACvsUC | 0.10699774 | -0.5880822 | 0.8020777 | 1 |
| [Cíntia E Botton](https://pubmed.ncbi.nlm.nih.gov/?term=Botton+CE&cauthor_id=30296453) | HIATvsUC | -0.27726434 | -0.9452534 | 0.3907247 | 1 |
| [Cíntia E Botton](https://pubmed.ncbi.nlm.nih.gov/?term=Botton+CE&cauthor_id=30296453) | HIAT-LIRTvsUC | -0.34186122 | -1.373829 | 0.6901065 | 1 |
| [Cíntia E Botton](https://pubmed.ncbi.nlm.nih.gov/?term=Botton+CE&cauthor_id=30296453) | HIAT-MIRTvsUC | -0.45440097 | -1.010323 | 0.101521 | 1 |
| [Cíntia E Botton](https://pubmed.ncbi.nlm.nih.gov/?term=Botton+CE&cauthor_id=30296453) | HIRTvsUC | -0.92125457 | -1.376703 | -0.4658063 | 1 |
| [Cíntia E Botton](https://pubmed.ncbi.nlm.nih.gov/?term=Botton+CE&cauthor_id=30296453) | LIRTvsUC | -0.57239268 | -1.18207 | 0.0372847 | 1 |
| [Cíntia E Botton](https://pubmed.ncbi.nlm.nih.gov/?term=Botton+CE&cauthor_id=30296453) | MIATvsUC | -0.58562781 | -1.253873 | 0.0826176 | 1 |
| [Cíntia E Botton](https://pubmed.ncbi.nlm.nih.gov/?term=Botton+CE&cauthor_id=30296453) | MIRTvsUC | -0.12025587 | -0.5854994 | 0.3449877 | 1 |
| N W Cheung | ACvsUC | 0.07129667 | -0.5999559 | 0.7425492 | 1 |
| N W Cheung | HIATvsUC | -0.3216026 | -0.9886701 | 0.3454649 | 1 |
| N W Cheung | HIAT-LIRTvsUC | -0.34186122 | -1.357291 | 0.673568 | 1 |
| N W Cheung | HIAT-MIRTvsUC | -0.48794983 | -1.042377 | 0.0664775 | 1 |
| N W Cheung | HIRTvsUC | -0.9256346 | -1.375051 | -0.4762179 | 1 |
| N W Cheung | LIRTvsUC | -0.57192549 | -1.174919 | 0.0310679 | 1 |
| N W Cheung | MIATvsUC | -0.58521989 | -1.245686 | 0.075246 | 1 |
| N W Cheung | MIRTvsUC | -0.21671269 | -0.7159751 | 0.2825497 | 1 |
| Yutaro Yamamoto | ACvsUC | 0.10939069 | -0.5700189 | 0.7888004 | 1 |
| Yutaro Yamamoto | HIATvsUC | -0.30080537 | -0.9750116 | 0.3734009 | 1 |
| Yutaro Yamamoto | HIAT-LIRTvsUC | -0.34186122 | -1.374509 | 0.6907865 | 1 |
| Yutaro Yamamoto | HIAT-MIRTvsUC | -0.47224796 | -1.0325 | 0.0880045 | 1 |
| Yutaro Yamamoto | HIRTvsUC | -0.92095395 | -1.376224 | -0.4656838 | 1 |
| Yutaro Yamamoto | LIRTvsUC | -0.57241162 | -1.182364 | 0.037541 | 1 |
| Yutaro Yamamoto | MIATvsUC | -0.58564434 | -1.25421 | 0.0829215 | 1 |
| Yutaro Yamamoto | MIRTvsUC | -0.17173223 | -0.6764569 | 0.3329924 | 1 |
| Yu-Hsuan Chien | ACvsUC | 0.16363359 | -0.518075 | 0.8453422 | 1 |
| Yu-Hsuan Chien | HIATvsUC | -0.27127214 | -0.9472194 | 0.4046751 | 1 |
| Yu-Hsuan Chien | HIAT-LIRTvsUC | -0.34186123 | -1.378363 | 0.6946403 | 1 |
| Yu-Hsuan Chien | HIAT-MIRTvsUC | -0.44987734 | -1.011538 | 0.1117828 | 1 |
| Yu-Hsuan Chien | HIRTvsUC | -0.91397597 | -1.37057 | -0.4573819 | 1 |
| Yu-Hsuan Chien | LIRTvsUC | -0.57251862 | -1.184034 | 0.0389971 | 1 |
| Yu-Hsuan Chien | MIATvsUC | -0.58573766 | -1.256122 | 0.0846463 | 1 |
| Yu-Hsuan Chien | MIRTvsUC | -0.10726184 | -0.614123 | 0.3995993 | 1 |
| Nikolaos P.E.K | ACvsUC | -0.0044082 | -0.6862919 | 0.6774755 | 1 |
| Nikolaos P.E.K | HIATvsUC | -0.27001334 | -0.9205971 | 0.3805704 | 1 |
| Nikolaos P.E.K | HIAT-LIRTvsUC | -0.34186122 | -1.331656 | 0.6479338 | 1 |
| Nikolaos P.E.K | HIAT-MIRTvsUC | -0.44872584 | -0.9904557 | 0.093004 | 1 |
| Nikolaos P.E.K | HIRTvsUC | -0.93497715 | -1.376301 | -0.4936536 | 1 |
| Nikolaos P.E.K | LIRTvsUC | -0.57117626 | -1.163882 | 0.0215291 | 1 |
| Nikolaos P.E.K | MIATvsUC | -0.5845641 | -1.233042 | 0.0639139 | 1 |
| Nikolaos P.E.K | MIRTvsUC | -0.10339858 | -0.5555357 | 0.3487386 | 1 |
| Ping-Lun Hsieh | ACvsUC | 0.12167946 | -0.526466 | 0.7698249 | 1 |
| Ping-Lun Hsieh | HIATvsUC | -0.28478004 | -0.942073 | 0.3725129 | 1 |
| Ping-Lun Hsieh | HIAT-LIRTvsUC | -0.34186122 | -1.348219 | 0.664497 | 1 |
| Ping-Lun Hsieh | HIAT-MIRTvsUC | -0.4599946 | -1.007206 | 0.0872164 | 1 |
| Ping-Lun Hsieh | HIRTvsUC | -1.0141785 | -1.494703 | -0.5336543 | 1 |
| Ping-Lun Hsieh | LIRTvsUC | -0.5716639 | -1.171005 | 0.0276777 | 1 |
| Ping-Lun Hsieh | MIATvsUC | -0.58499115 | -1.241204 | 0.0712221 | 1 |
| Ping-Lun Hsieh | MIRTvsUC | -0.13604749 | -0.5926707 | 0.3205757 | 1 |
| Anderson Rech | ACvsUC | 0.16027313 | -0.5453036 | 0.8658499 | 1 |
| Anderson Rech | HIATvsUC | -0.28062465 | -0.9515219 | 0.3902726 | 1 |
| Anderson Rech | HIAT-LIRTvsUC | -0.34186123 | -1.380731 | 0.6970087 | 1 |
| Anderson Rech | HIAT-MIRTvsUC | -0.45697563 | -1.015266 | 0.1013147 | 1 |
| Anderson Rech | HIRTvsUC | -0.91444128 | -1.372416 | -0.4564671 | 1 |
| Anderson Rech | LIRTvsUC | -0.57258403 | -1.185061 | 0.0398927 | 1 |
| Anderson Rech | MIATvsUC | -0.58579468 | -1.257296 | 0.0857071 | 1 |
| Anderson Rech | MIRTvsUC | -0.12776561 | -0.5954274 | 0.3398961 | 1 |
| George Mavros | ACvsUC | 0.25785392 | -0.4487955 | 0.9645033 | 1 |
| George Mavros | HIATvsUC | -0.28655859 | -0.949729 | 0.3766118 | 1 |
| George Mavros | HIAT-LIRTvsUC | -0.34186126 | -1.362075 | 0.6783522 | 1 |
| George Mavros | HIAT-MIRTvsUC | -0.46139939 | -1.013382 | 0.0905832 | 1 |
| George Mavros | HIRTvsUC | -0.90158489 | -1.353475 | -0.4496948 | 1 |
| George Mavros | LIRTvsUC | -0.57206203 | -1.176987 | 0.0328631 | 1 |
| George Mavros | MIATvsUC | -0.58533919 | -1.248053 | 0.0773749 | 1 |
| George Mavros | MIRTvsUC | -0.1402817 | -0.6022307 | 0.3216673 | 1 |
| Theng Choon Ooi | ACvsUC | 0.1886137 | -0.4408103 | 0.8180377 | 1 |
| Theng Choon Ooi | HIATvsUC | -0.27177183 | -0.9127318 | 0.3691881 | 1 |
| Theng Choon Ooi | HIAT-LIRTvsUC | -0.34186122 | -1.308338 | 0.6246155 | 1 |
| Theng Choon Ooi | HIAT-MIRTvsUC | -0.44995637 | -0.9838563 | 0.0839436 | 1 |
| Theng Choon Ooi | HIRTvsUC | -0.7686574 | -1.239475 | -0.2978393 | 1 |
| Theng Choon Ooi | LIRTvsUC | -0.57046732 | -1.153889 | 0.0129543 | 1 |
| Theng Choon Ooi | MIATvsUC | -0.58394175 | -1.221589 | 0.0537056 | 1 |
| Theng Choon Ooi | MIRTvsUC | -0.10664184 | -0.5508136 | 0.3375299 | 1 |
| Xiaojun Ma | ACvsUC | 0.13766389 | -0.5258947 | 0.8012225 | 1 |
| Xiaojun Ma | HIATvsUC | -0.28179356 | -0.9520748 | 0.3884876 | 1 |
| Xiaojun Ma | HIAT-LIRTvsUC | -0.34186123 | -1.37947 | 0.6957478 | 1 |
| Xiaojun Ma | HIAT-MIRTvsUC | -0.45785661 | -1.015655 | 0.0999419 | 1 |
| Xiaojun Ma | HIRTvsUC | -0.95355929 | -1.454181 | -0.452938 | 1 |
| Xiaojun Ma | LIRTvsUC | -0.57254923 | -1.184514 | 0.0394157 | 1 |
| Xiaojun Ma | MIATvsUC | -0.58576434 | -1.256671 | 0.0851422 | 1 |
| Xiaojun Ma | MIRTvsUC | -0.13029147 | -0.5969256 | 0.3363427 | 1 |
| Carmen Castaneda | ACvsUC | 0.22783728 | -0.2812841 | 0.7369587 | 1 |
| Carmen Castaneda | HIATvsUC | -0.26190038 | -0.8020027 | 0.278202 | 1 |
| Carmen Castaneda | HIAT-LIRTvsUC | -0.34186122 | -1.044131 | 0.3604085 | 1 |
| Carmen Castaneda | HIAT-MIRTvsUC | -0.4410018 | -0.8928968 | 0.0108932 | 1 |
| Carmen Castaneda | HIRTvsUC | -0.62150076 | -0.9947223 | -0.2482792 | 1 |
| Carmen Castaneda | LIRTvsUC | -0.5603542 | -1.044883 | -0.0758256 | 1 |
| Carmen Castaneda | MIATvsUC | -0.57485641 | -1.096098 | -0.053615 | 1 |
| Carmen Castaneda | MIRTvsUC | -0.07676964 | -0.4423033 | 0.2887641 | 1 |
| David W Dunstan | ACvsUC | 0.2877263 | -0.4648938 | 1.040346 | 1 |
| David W Dunstan | HIATvsUC | -0.25305574 | -0.9207379 | 0.4146264 | 1 |
| David W Dunstan | HIAT-LIRTvsUC | -0.34186121 | -1.364261 | 0.6805388 | 1 |
| David W Dunstan | HIAT-MIRTvsUC | -0.4360093 | -0.9912248 | 0.1192062 | 1 |
| David W Dunstan | HIRTvsUC | -0.97143124 | -1.446556 | -0.4963063 | 1 |
| David W Dunstan | LIRTvsUC | -0.57212391 | -1.17793 | 0.0336822 | 1 |
| David W Dunstan | MIATvsUC | -0.58539323 | -1.249134 | 0.0783473 | 1 |
| David W Dunstan | MIRTvsUC | -0.06713032 | -0.5539196 | 0.4196589 | 1 |
| Kiwol Sung | ACvsUC | 0.14799445 | -0.5098678 | 0.8058568 | 1 |
| Kiwol Sung | HIATvsUC | -0.2798033 | -0.9470934 | 0.3874868 | 1 |
| Kiwol Sung | HIAT-LIRTvsUC | -0.34186122 | -1.372496 | 0.6887739 | 1 |
| Kiwol Sung | HIAT-MIRTvsUC | -0.45632019 | -1.01169 | 0.0990501 | 1 |
| Kiwol Sung | HIRTvsUC | -0.9159271 | -1.37 | -0.461854 | 1 |
| Kiwol Sung | LIRTvsUC | -0.60707978 | -1.230479 | 0.0163195 | 1 |
| Kiwol Sung | MIATvsUC | -0.70783478 | -1.522507 | 0.1068373 | 1 |
| Kiwol Sung | MIRTvsUC | -0.12577398 | -0.5896137 | 0.3380657 | 1 |
| Hwi Ryun Kwon | ACvsUC | 0.14807927 | -0.5074794 | 0.803638 | 1 |
| Hwi Ryun Kwon | HIATvsUC | -0.27977374 | -0.945058 | 0.3855105 | 1 |
| Hwi Ryun Kwon | HIAT-LIRTvsUC | -0.34186122 | -1.367674 | 0.6839514 | 1 |
| Hwi Ryun Kwon | HIAT-MIRTvsUC | -0.45627833 | -1.010013 | 0.0974562 | 1 |
| Hwi Ryun Kwon | HIRTvsUC | -0.91585288 | -1.368285 | -0.4634206 | 1 |
| Hwi Ryun Kwon | LIRTvsUC | -0.68401418 | -1.382522 | 0.0144932 | 1 |
| Hwi Ryun Kwon | MIATvsUC | -0.62361251 | -1.299333 | 0.0521083 | 1 |
| Hwi Ryun Kwon | MIRTvsUC | -0.12559347 | -0.5879008 | 0.3367138 | 1 |
| Maryam Nadi | ACvsUC | 0.14933352 | -0.4732378 | 0.7719048 | 1 |
| Maryam Nadi | HIATvsUC | -0.27931885 | -0.9160202 | 0.3573825 | 1 |
| Maryam Nadi | HIAT-LIRTvsUC | -0.34186122 | -1.298031 | 0.6143085 | 1 |
| Maryam Nadi | HIAT-MIRTvsUC | -0.45563649 | -0.9860744 | 0.0748014 | 1 |
| Maryam Nadi | HIRTvsUC | -0.91468416 | -1.343612 | -0.4857561 | 1 |
| Maryam Nadi | LIRTvsUC | -0.28369982 | -0.9406313 | 0.3732316 | 1 |
| Maryam Nadi | MIATvsUC | -0.48661208 | -1.128067 | 0.1548424 | 1 |
| Maryam Nadi | MIRTvsUC | -0.12281418 | -0.5632313 | 0.317603 | 1 |
| R C Plotnikoff | ACvsUC | 0.09764116 | -0.5059156 | 0.701198 | 1 |
| R C Plotnikoff | HIATvsUC | -0.28923493 | -0.9079661 | 0.3294962 | 1 |
| R C Plotnikoff | HIAT-LIRTvsUC | -0.34186122 | -1.25307 | 0.5693474 | 1 |
| R C Plotnikoff | HIAT-MIRTvsUC | -0.46295817 | -0.9787513 | 0.052835 | 1 |
| R C Plotnikoff | HIRTvsUC | -1.1097451 | -1.561937 | -0.6575534 | 1 |
| R C Plotnikoff | LIRTvsUC | -0.56867742 | -1.130406 | -0.0069484 | 1 |
| R C Plotnikoff | MIATvsUC | -0.58236244 | -1.194648 | 0.0299229 | 1 |
| R C Plotnikoff | MIRTvsUC | -0.14309682 | -0.5701206 | 0.2839269 | 1 |
| Lauren M Sparks | ACvsUC | 0.09128775 | -0.6070423 | 0.7896178 | 1 |
| Lauren M Sparks | HIATvsUC | -0.20342815 | -1.031624 | 0.6247674 | 1 |
| Lauren M Sparks | HIAT-LIRTvsUC | -0.34186122 | -1.414662 | 0.73094 | 1 |
| Lauren M Sparks | HIAT-MIRTvsUC | -0.46798835 | -1.108854 | 0.172877 | 1 |
| Lauren M Sparks | HIRTvsUC | -0.92383563 | -1.392834 | -0.4548373 | 1 |
| Lauren M Sparks | LIRTvsUC | -0.57349403 | -1.199822 | 0.0528339 | 1 |
| Lauren M Sparks | MIATvsUC | -0.5865865 | -1.274183 | 0.1010097 | 1 |
| Lauren M Sparks | MIRTvsUC | -0.19396768 | -0.7110615 | 0.3231261 | 1 |
| Vanessa Neves de Oliveira | ACvsUC | 0.17363034 | -0.5185379 | 0.8657987 | 1 |
| Vanessa Neves de Oliveira | HIATvsUC | -0.38735476 | -1.207681 | 0.4329717 | 1 |
| Vanessa Neves de Oliveira | HIAT-LIRTvsUC | -0.34186122 | -1.406754 | 0.7230312 | 1 |
| Vanessa Neves de Oliveira | HIAT-MIRTvsUC | -0.557958 | -1.192456 | 0.0765396 | 1 |
| Vanessa Neves de Oliveira | HIRTvsUC | -0.91301825 | -1.379233 | -0.4468037 | 1 |
| Vanessa Neves de Oliveira | LIRTvsUC | -0.57328636 | -1.196376 | 0.0498029 | 1 |
| Vanessa Neves de Oliveira | MIATvsUC | -0.58640604 | -1.27024 | 0.0974284 | 1 |
| Vanessa Neves de Oliveira | MIRTvsUC | -0.09577994 | -0.6057105 | 0.4141506 | 1 |
| Alireza Mehdizadeh | ACvsUC | 0.21120185 | -0.4773915 | 0.8997952 | 1 |
| Alireza Mehdizadeh | HIATvsUC | -0.24641753 | -1.055409 | 0.5625736 | 1 |
| Alireza Mehdizadeh | HIAT-LIRTvsUC | -0.34186122 | -1.400956 | 0.7172334 | 1 |
| Alireza Mehdizadeh | HIAT-MIRTvsUC | -0.38778293 | -1.01693 | 0.2413645 | 1 |
| Alireza Mehdizadeh | HIRTvsUC | -0.90806569 | -1.372269 | -0.4438624 | 1 |
| Alireza Mehdizadeh | LIRTvsUC | -0.57313242 | -1.193852 | 0.047587 | 1 |
| Alireza Mehdizadeh | MIATvsUC | -0.58627219 | -1.267353 | 0.0948088 | 1 |
| Alireza Mehdizadeh | MIRTvsUC | -0.05096491 | -0.5574684 | 0.4555386 | 1 |
| Hwi Ryun Kwon | ACvsUC | 0.14743927 | -0.5257791 | 0.8206577 | 1 |
| Hwi Ryun Kwon | HIATvsUC | -0.27999364 | -0.9606853 | 0.4006981 | 1 |
| Hwi Ryun Kwon | HIAT-LIRTvsUC | -0.34186122 | -1.404519 | 0.7207963 | 1 |
| Hwi Ryun Kwon | HIAT-MIRTvsUC | -0.45659015 | -1.022892 | 0.1097112 | 1 |
| Hwi Ryun Kwon | HIRTvsUC | -0.91639993 | -1.381408 | -0.4513916 | 1 |
| Hwi Ryun Kwon | LIRTvsUC | -0.60615457 | -1.324185 | 0.1118757 | 1 |
| Hwi Ryun Kwon | MIATvsUC | -0.56037222 | -1.384571 | 0.2638266 | 1 |
| Hwi Ryun Kwon | MIRTvsUC | -0.12693586 | -0.6010032 | 0.3471315 | 1 |
| Y H Ku | ACvsUC | 0.14765182 | -0.5196294 | 0.814933 | 1 |
| Y H Ku | HIATvsUC | -0.27992139 | -0.9554251 | 0.3955823 | 1 |
| Y H Ku | HIAT-LIRTvsUC | -0.34186121 | -1.392162 | 0.7084394 | 1 |
| Y H Ku | HIAT-MIRTvsUC | -0.4564876 | -1.018557 | 0.1055817 | 1 |
| Y H Ku | HIRTvsUC | -0.91622148 | -1.377002 | -0.4554405 | 1 |
| Y H Ku | LIRTvsUC | -0.70325327 | -1.419462 | 0.0129552 | 1 |
| Y H Ku | MIATvsUC | -0.54008794 | -1.36503 | 0.2848546 | 1 |
| Y H Ku | MIRTvsUC | -0.12649496 | -0.5966086 | 0.3436188 | 1 |
| C Blioumpa | ACvsUC | 0.15680138 | -0.5029012 | 0.816504 | 1 |
| C Blioumpa | HIATvsUC | -0.26195038 | -0.937598 | 0.4136972 | 1 |
| C Blioumpa | HIAT-LIRTvsUC | -0.34186122 | -1.37189 | 0.6881679 | 1 |
| C Blioumpa | HIAT-MIRTvsUC | -0.41301482 | -1.0264 | 0.2003703 | 1 |
| C Blioumpa | HIRTvsUC | -0.9147788 | -1.368696 | -0.4608613 | 1 |
| C Blioumpa | LIRTvsUC | -0.57233856 | -1.181231 | 0.0365534 | 1 |
| C Blioumpa | MIATvsUC | -0.5855806 | -1.252912 | 0.0817511 | 1 |
| C Blioumpa | MIRTvsUC | -0.11528767 | -0.5831901 | 0.3526148 | 1 |
| Karolina S Khan | ACvsUC | 0.13187499 | -0.5254625 | 0.7892125 | 1 |
| Karolina S Khan | HIATvsUC | -0.28287096 | -0.9481621 | 0.3824202 | 1 |
| Karolina S Khan | HIAT-LIRTvsUC | -0.34186122 | -1.36753 | 0.683808 | 1 |
| Karolina S Khan | HIAT-MIRTvsUC | -0.45862568 | -1.012358 | 0.0951065 | 1 |
| Karolina S Khan | HIRTvsUC | -0.97549918 | -1.462503 | -0.4884954 | 1 |
| Karolina S Khan | LIRTvsUC | -0.57221622 | -1.179344 | 0.0349114 | 1 |
| Karolina S Khan | MIATvsUC | -0.58547382 | -1.250752 | 0.0798047 | 1 |
| Karolina S Khan | MIRTvsUC | -0.13235855 | -0.5950884 | 0.3303713 | 1 |
| Francesca Galle | ACvsUC | 0.14837932 | -0.4991132 | 0.7958719 | 1 |
| Francesca Galle | HIATvsUC | -0.27966804 | -0.9379389 | 0.3786029 | 1 |
| Francesca Galle | HIAT-LIRTvsUC | -0.45612881 | -1.004145 | 0.0918872 | 1 |
| Francesca Galle | HIAT-MIRTvsUC | -0.91558581 | -1.362272 | -0.4688998 | 1 |
| Francesca Galle | HIRTvsUC | -0.57173715 | -1.172093 | 0.0286184 | 1 |
| Francesca Galle | LIRTvsUC | -0.58505521 | -1.242451 | 0.0723406 | 1 |
| Francesca Galle | MIATvsUC | -0.12494795 | -0.5818877 | 0.3319918 | 1 |
| Nafiseh Ghodrati PhD | ACvsUC | 0.15122024 | -0.5093725 | 0.8118129 | 1 |
| Nafiseh Ghodrati PhD | HIATvsUC | -0.27321807 | -0.9494851 | 0.403049 | 1 |
| Nafiseh Ghodrati PhD | HIAT-LIRTvsUC | -0.34186122 | -1.373853 | 0.6901304 | 1 |
| Nafiseh Ghodrati PhD | HIAT-MIRTvsUC | -0.44033767 | -1.053103 | 0.1724272 | 1 |
| Nafiseh Ghodrati PhD | HIRTvsUC | -0.91552693 | -1.370112 | -0.4609418 | 1 |
| Nafiseh Ghodrati PhD | LIRTvsUC | -0.57239335 | -1.18208 | 0.0372936 | 1 |
| Nafiseh Ghodrati PhD | MIATvsUC | -0.5856284 | -1.253885 | 0.0826282 | 1 |
| Nafiseh Ghodrati PhD | MIRTvsUC | -0.12195868 | -0.5903952 | 0.3464778 | 1 |

Supplementary Table20.Leave-one-out sensitivity analysis for FPG in middle-aged and older adults with type 2 diabetes.

| **dropped_id** | **comparison** | **eff** | **lci** | **uci** | **connected** |
| --- | --- | --- | --- | --- | --- |
| Nikolaos P.E.K | ACvsUC | -0.23590596 | -1.132286 | 0.6604738 | 1 |
| Nikolaos P.E.K | HIATvsUC | -0.64374292 | -1.48391 | 0.1964242 | 1 |
| Nikolaos P.E.K | HIAT-MIRTvsUC | -0.45805958 | -1.267931 | 0.3518122 | 1 |
| Nikolaos P.E.K | HIRTvsUC | -0.39500332 | -0.9367346 | 0.1467279 | 1 |
| Nikolaos P.E.K | LIRTvsUC | -0.63795635 | -1.604704 | 0.3287917 | 1 |
| Nikolaos P.E.K | MIATvsUC | -0.72652074 | -1.635834 | 0.1827926 | 1 |
| Nikolaos P.E.K | MIRTvsUC | -0.61214894 | -1.489275 | 0.2649773 | 1 |
| Ping-Lun Hsieh | ACvsUC | -0.11760069 | -0.9899305 | 0.7547292 | 1 |
| Ping-Lun Hsieh | HIATvsUC | -0.60631843 | -1.454744 | 0.2421069 | 1 |
| Ping-Lun Hsieh | HIAT-MIRTvsUC | -0.46673225 | -1.28399 | 0.3505255 | 1 |
| Ping-Lun Hsieh | HIRTvsUC | -0.31713256 | -0.9135175 | 0.2792524 | 1 |
| Ping-Lun Hsieh | LIRTvsUC | -0.63994785 | -1.616572 | 0.3366766 | 1 |
| Ping-Lun Hsieh | MIATvsUC | -0.72743991 | -1.647341 | 0.1924616 | 1 |
| Ping-Lun Hsieh | MIRTvsUC | -0.68420465 | -1.538895 | 0.1704855 | 1 |
| Anderson Rech | ACvsUC | 0.03542697 | -0.8089429 | 0.8797969 | 1 |
| Anderson Rech | HIATvsUC | -0.57548443 | -1.370839 | 0.2198704 | 1 |
| Anderson Rech | HIAT-MIRTvsUC | -0.50009262 | -1.268356 | 0.2681706 | 1 |
| Anderson Rech | HIRTvsUC | -0.35358003 | -0.8600468 | 0.1528868 | 1 |
| Anderson Rech | LIRTvsUC | -0.62679698 | -1.543109 | 0.2895153 | 1 |
| Anderson Rech | MIATvsUC | -0.72138633 | -1.576486 | 0.1337132 | 1 |
| Anderson Rech | MIRTvsUC | -0.85306078 | -1.682248 | -0.0238739 | 1 |
| Theng Choon Ooi | ACvsUC | -0.09884948 | -0.9563603 | 0.7586614 | 1 |
| Theng Choon Ooi | HIATvsUC | -0.59455852 | -1.42874 | 0.2396234 | 1 |
| Theng Choon Ooi | HIAT-MIRTvsUC | -0.46033173 | -1.264156 | 0.3434921 | 1 |
| Theng Choon Ooi | HIRTvsUC | -0.27000446 | -0.8634209 | 0.323412 | 1 |
| Theng Choon Ooi | LIRTvsUC | -0.6365874 | -1.596721 | 0.3235462 | 1 |
| Theng Choon Ooi | MIATvsUC | -0.7258894 | -1.6281 | 0.1763209 | 1 |
| Theng Choon Ooi | MIRTvsUC | -0.67142404 | -1.512106 | 0.1692584 | 1 |
| Xiaojun Ma | ACvsUC | -0.14188547 | -1.031261 | 0.7474902 | 1 |
| Xiaojun Ma | HIATvsUC | -0.6214178 | -1.484986 | 0.2421506 | 1 |
| Xiaojun Ma | HIAT-MIRTvsUC | -0.47479367 | -1.305774 | 0.3561869 | 1 |
| Xiaojun Ma | HIRTvsUC | -0.37913017 | -0.9992191 | 0.2409588 | 1 |
| Xiaojun Ma | LIRTvsUC | -0.64314601 | -1.636361 | 0.3500694 | 1 |
| Xiaojun Ma | MIATvsUC | -0.72891779 | -1.666506 | 0.2086705 | 1 |
| Xiaojun Ma | MIRTvsUC | -0.70031419 | -1.570055 | 0.1694265 | 1 |
| Carmen Castaneda | ACvsUC | -0.17269819 | -1.047219 | 0.7018224 | 1 |
| Carmen Castaneda | HIATvsUC | -0.64026693 | -1.490102 | 0.2095685 | 1 |
| Carmen Castaneda | HIAT-MIRTvsUC | -0.48391255 | -1.302206 | 0.334381 | 1 |
| Carmen Castaneda | HIRTvsUC | -0.46288966 | -1.071195 | 0.1454158 | 1 |
| Carmen Castaneda | LIRTvsUC | -0.64017553 | -1.617966 | 0.3376153 | 1 |
| Carmen Castaneda | MIATvsUC | -0.72754505 | -1.648681 | 0.1935908 | 1 |
| Carmen Castaneda | MIRTvsUC | -0.71866631 | -1.574716 | 0.1373834 | 1 |
| David W Dunstan | ACvsUC | -0.18067799 | -1.260563 | 0.899207 | 1 |
| David W Dunstan | HIATvsUC | -0.645224 | -1.5843 | 0.2938517 | 1 |
| David W Dunstan | HIAT-MIRTvsUC | -0.48664862 | -1.334532 | 0.3612352 | 1 |
| David W Dunstan | HIRTvsUC | -0.37024241 | -0.9539706 | 0.2134858 | 1 |
| David W Dunstan | LIRTvsUC | -0.6419001 | -1.62855 | 0.3447501 | 1 |
| David W Dunstan | MIATvsUC | -0.72834179 | -1.658938 | 0.2022547 | 1 |
| David W Dunstan | MIRTvsUC | -0.72411995 | -1.670956 | 0.2227159 | 1 |
| Kenneth M Madden | ACvsUC | -0.19758012 | -1.10989 | 0.7147295 | 1 |
| Kenneth M Madden | HIATvsUC | -0.57312059 | -1.455627 | 0.3093857 | 1 |
| Kenneth M Madden | HIAT-MIRTvsUC | -0.46591565 | -1.288495 | 0.3566639 | 1 |
| Kenneth M Madden | HIRTvsUC | -0.38962141 | -0.9421787 | 0.1629358 | 1 |
| Kenneth M Madden | LIRTvsUC | -0.64108217 | -1.623494 | 0.3413292 | 1 |
| Kenneth M Madden | MIATvsUC | -0.72796383 | -1.654041 | 0.1981137 | 1 |
| Kenneth M Madden | MIRTvsUC | -0.71417849 | -1.574777 | 0.1464199 | 1 |
| Kenneth M Madden | ACvsUC | -0.15470912 | -1.074319 | 0.7649012 | 1 |
| Kenneth M Madden | HIATvsUC | -0.61129592 | -1.500294 | 0.277702 | 1 |
| Kenneth M Madden | HIAT-MIRTvsUC | -0.47301284 | -1.300985 | 0.3549598 | 1 |
| Kenneth M Madden | HIRTvsUC | -0.38330715 | -0.9403861 | 0.1737717 | 1 |
| Kenneth M Madden | LIRTvsUC | -0.64234438 | -1.631323 | 0.3466339 | 1 |
| Kenneth M Madden | MIATvsUC | -0.72854715 | -1.661622 | 0.2045279 | 1 |
| Kenneth M Madden | MIRTvsUC | -0.70364597 | -1.570122 | 0.1628299 | 1 |
| Kiwol Sung | ACvsUC | -0.14253207 | -1.021744 | 0.7366794 | 1 |
| Kiwol Sung | HIATvsUC | -0.62177737 | -1.47906 | 0.2355053 | 1 |
| Kiwol Sung | HIAT-MIRTvsUC | -0.47485872 | -1.301513 | 0.351796 | 1 |
| Kiwol Sung | HIRTvsUC | -0.38146688 | -0.9368608 | 0.173927 | 1 |
| Kiwol Sung | LIRTvsUC | -0.64957502 | -1.674389 | 0.375239 | 1 |
| Kiwol Sung | MIATvsUC | -0.75303789 | -2.050037 | 0.5439609 | 1 |
| Kiwol Sung | MIRTvsUC | -0.70046179 | -1.563891 | 0.1629672 | 1 |
| Maryam Nadi | ACvsUC | -0.11652853 | -0.7455214 | 0.5124643 | 1 |
| Maryam Nadi | HIATvsUC | -0.60352021 | -1.229267 | 0.0222261 | 1 |
| Maryam Nadi | HIAT-MIRTvsUC | -0.45490538 | -1.069373 | 0.1595622 | 1 |
| Maryam Nadi | HIRTvsUC | -0.36882709 | -0.7381078 | 0.0004536 | 1 |
| Maryam Nadi | LIRTvsUC | 0.53698641 | -0.3965534 | 1.470526 | 1 |
| Maryam Nadi | MIATvsUC | -0.42208938 | -1.085893 | 0.2417146 | 1 |
| Maryam Nadi | MIRTvsUC | -0.66116548 | -1.295128 | -0.0272029 | 1 |
| R C Plotnikoff | ACvsUC | -0.18210602 | -1.042317 | 0.6781045 | 1 |
| R C Plotnikoff | HIATvsUC | -0.64599635 | -1.482745 | 0.1907523 | 1 |
| R C Plotnikoff | HIAT-MIRTvsUC | -0.48641065 | -1.292688 | 0.3198667 | 1 |
| R C Plotnikoff | HIRTvsUC | -0.48984863 | -1.083869 | 0.104172 | 1 |
| R C Plotnikoff | LIRTvsUC | -0.63723001 | -1.600406 | 0.325946 | 1 |
| R C Plotnikoff | MIATvsUC | -0.72618571 | -1.631703 | 0.1793313 | 1 |
| R C Plotnikoff | MIRTvsUC | -0.72373652 | -1.566805 | 0.1193315 | 1 |
| Vanessa Neves de Oliveira | ACvsUC | -0.44247249 | -1.455196 | 0.5702509 | 1 |
| Vanessa Neves de Oliveira | HIATvsUC | -0.99789069 | -2.080539 | 0.0847576 | 1 |
| Vanessa Neves de Oliveira | HIAT-MIRTvsUC | -0.78178659 | -1.792736 | 0.2291632 | 1 |
| Vanessa Neves de Oliveira | HIRTvsUC | -0.42666478 | -0.9877122 | 0.1343826 | 1 |
| Vanessa Neves de Oliveira | LIRTvsUC | -0.64240256 | -1.632048 | 0.347243 | 1 |
| Vanessa Neves de Oliveira | MIATvsUC | -0.72857406 | -1.662056 | 0.204908 | 1 |
| Vanessa Neves de Oliveira | MIRTvsUC | -1.0424241 | -2.13126 | 0.0464116 | 1 |
| Alireza Mehdizadeh | ACvsUC | 0.15632823 | -0.8179166 | 1.130573 | 1 |
| Alireza Mehdizadeh | HIATvsUC | -0.33162435 | -1.368627 | 0.7053782 | 1 |
| Alireza Mehdizadeh | HIAT-MIRTvsUC | -0.19975389 | -1.181154 | 0.7816465 | 1 |
| Alireza Mehdizadeh | HIRTvsUC | -0.33629204 | -0.8835022 | 0.2109181 | 1 |
| Alireza Mehdizadeh | LIRTvsUC | -0.638759 | -1.609781 | 0.3322628 | 1 |
| Alireza Mehdizadeh | MIATvsUC | -0.72689111 | -1.640509 | 0.186727 | 1 |
| Alireza Mehdizadeh | MIRTvsUC | -0.27694537 | -1.318025 | 0.764134 | 1 |
| Y H Ku | ACvsUC | -0.11745705 | -0.7524538 | 0.5175397 | 1 |
| Y H Ku | HIATvsUC | -0.60413013 | -1.235371 | 0.0271103 | 1 |
| Y H Ku | HIAT-MIRTvsUC | -0.4555991 | -1.07503 | 0.1638322 | 1 |
| Y H Ku | HIRTvsUC | -0.36930233 | -0.7432471 | 0.0046424 | 1 |
| Y H Ku | LIRTvsUC | -2.1962594 | -3.34149 | -1.051028 | 1 |
| Y H Ku | MIATvsUC | -0.70220157 | -1.629912 | 0.2255087 | 1 |
| Y H Ku | MIRTvsUC | -0.66255994 | -1.302097 | -0.0230226 | 1 |
| Nafiseh Ghodrati PhD | ACvsUC | -0.13173502 | -1.026531 | 0.7630613 | 1 |
| Nafiseh Ghodrati PhD | HIATvsUC | -0.60907774 | -1.492137 | 0.2739814 | 1 |
| Nafiseh Ghodrati PhD | HIAT-MIRTvsUC | -0.44496416 | -1.437592 | 0.5476635 | 1 |
| Nafiseh Ghodrati PhD | HIRTvsUC | -0.3797149 | -0.9314764 | 0.1720466 | 1 |
| Nafiseh Ghodrati PhD | LIRTvsUC | -0.64104315 | -1.623253 | 0.341167 | 1 |
| Nafiseh Ghodrati PhD | MIATvsUC | -0.7279458 | -1.653809 | 0.1979175 | 1 |
| Nafiseh Ghodrati PhD | MIRTvsUC | -0.68737973 | -1.576796 | 0.2020368 | 1 |

Supplementary Table21.Leave-one-out sensitivity analysis forVO_2_peak in middle-aged and older adults with type 2 diabetes.

| **dropped_id** | **comparison** | **eff** | **lci** | **uci** | **connected** |
| --- | --- | --- | --- | --- | --- |
| Nikolaos P.E.K | ACvsUC | 0.76924658 | 0.2945712 | 1.243922 | 1 |
| Nikolaos P.E.K | HIATvsUC | 2.1388263 | 0.4824626 | 3.79519 | 1 |
| Nikolaos P.E.K | HIAT-HIRTvsUC | 0.47800258 | -0.0006221 | 0.9566272 | 1 |
| Nikolaos P.E.K | HIAT-MIRTvsUC | 0.05752968 | -0.4746575 | 0.5897169 | 1 |
| Nikolaos P.E.K | HIRTvsUC | 0.25475842 | -0.2640124 | 0.7735292 | 1 |
| Ping-Lun Hsieh | ACvsUC | 0.1659374 | -0.6079719 | 0.9398467 | 1 |
| Ping-Lun Hsieh | HIATvsUC | 0.78172966 | 0.2958228 | 1.267637 | 1 |
| Ping-Lun Hsieh | HIAT-HIRTvsUC | 2.2099592 | 0.4510807 | 3.968838 | 1 |
| Ping-Lun Hsieh | HIAT-MIRTvsUC | 0.48278897 | 0.0025103 | 0.9630677 | 1 |
| Ping-Lun Hsieh | HIRTvsUC | 0.1286816 | -0.6672602 | 0.9246234 | 1 |
| Ping-Lun Hsieh | MIRTvsUC | 0.26058704 | -0.2604375 | 0.7816116 | 1 |
| Lauren M Sparks | disconnected |  |  |  | 0 |
| Vanessa Neves de Oliveira | disconnected |  |  |  | 0 |
| Giorgio Orlando | ACvsUC | 0.16010017 | -0.6122816 | 0.9324819 | 1 |
| Giorgio Orlando | HIATvsUC | 0.76925288 | 0.2945799 | 1.243926 | 1 |
| Giorgio Orlando | HIAT-HIRTvsUC | 0.47800153 | -0.0006203 | 0.9566233 | 1 |
| Giorgio Orlando | HIAT-MIRTvsUC | 0.05759661 | -0.4746101 | 0.5898033 | 1 |
| Giorgio Orlando | HIRTvsUC | 0.25474955 | -0.2640032 | 0.7735023 | 1 |
| Niloufar Ghadamyari | ACvsUC | 0.09053828 | -0.6954818 | 0.8765584 | 1 |
| Niloufar Ghadamyari | HIATvsUC | 0.62056787 | 0.0527498 | 1.188386 | 1 |
| Niloufar Ghadamyari | HIAT-HIRTvsUC | 2.0811964 | 0.3571487 | 3.805244 | 1 |
| Niloufar Ghadamyari | HIAT-MIRTvsUC | 0.42094992 | -0.072381 | 0.9142809 | 1 |
| Niloufar Ghadamyari | HIRTvsUC | -0.0001156 | -0.7157587 | 0.7155275 | 1 |
| Niloufar Ghadamyari | MIRTvsUC | 0.18518463 | -0.3536663 | 0.7240355 | 1 |
| Ramin Shabani | ACvsUC | 0.15704396 | -0.6283117 | 0.9423996 | 1 |
| Ramin Shabani | HIATvsUC | 0.76672799 | 0.2778237 | 1.255632 | 1 |
| Ramin Shabani | HIAT-HIRTvsUC | 2.1382707 | 0.4817057 | 3.794836 | 1 |
| Ramin Shabani | HIAT-MIRTvsUC | 0.47133846 | -0.0990651 | 1.041742 | 1 |
| Ramin Shabani | HIRTvsUC | 0.056974 | -0.4758399 | 0.5897879 | 1 |
| Ramin Shabani | MIRTvsUC | 0.25169321 | -0.2861879 | 0.7895743 | 1 |

Supplementary Table22.Leave-one-out sensitivity analysis for SBP in middle-aged and older adults with type 2 diabetes.

| **dropped_id** | **comparison** | **eff** | **lci** | **uci** | **connected** |
| --- | --- | --- | --- | --- | --- |
| Nikolaos P.E.K | ACvsUC | 2.1888593 | 1.342045 | 3.035673 | 1 |
| Nikolaos P.E.K | HIATvsUC | -0.44148416 | -1.016747 | 0.1337791 | 1 |
| Nikolaos P.E.K | HIAT-MIRTvsUC | -0.00894443 | -0.8023463 | 0.7844574 | 1 |
| Nikolaos P.E.K | HIRTvsUC | -0.17647383 | -0.4228903 | 0.0699426 | 1 |
| Nikolaos P.E.K | LIRTvsUC | -0.24162834 | -0.9602968 | 0.4770401 | 1 |
| Nikolaos P.E.K | MIRTvsUC | -0.16052864 | -0.9524466 | 0.6313893 | 1 |
| Theng Choon Ooi | ACvsUC | 1.4498543 | 0.3198304 | 2.579878 | 1 |
| Theng Choon Ooi | HIATvsUC | -0.71366923 | -1.591273 | 0.1639346 | 1 |
| Theng Choon Ooi | HIAT-MIRTvsUC | 0.07208902 | -1.167479 | 1.311657 | 1 |
| Theng Choon Ooi | HIRTvsUC | -0.24910226 | -0.8399284 | 0.3417239 | 1 |
| Theng Choon Ooi | LIRTvsUC | -0.24162834 | -1.499171 | 1.015915 | 1 |
| Theng Choon Ooi | MIRTvsUC | 0.38829976 | -0.7142833 | 1.490883 | 1 |
| Xiaojun Ma | ACvsUC | 1.4664743 | 0.3461179 | 2.586831 | 1 |
| Xiaojun Ma | HIATvsUC | -0.69347837 | -1.563843 | 0.176886 | 1 |
| Xiaojun Ma | HIAT-MIRTvsUC | 0.083586 | -1.145691 | 1.312863 | 1 |
| Xiaojun Ma | HIRTvsUC | -0.17572211 | -0.7617407 | 0.4102965 | 1 |
| Xiaojun Ma | LIRTvsUC | -0.24162834 | -1.487225 | 1.003968 | 1 |
| Xiaojun Ma | MIRTvsUC | 0.40329465 | -0.6902692 | 1.496858 | 1 |
| Carmen Castaneda | ACvsUC | 1.4363439 | 0.3338613 | 2.538826 | 1 |
| Carmen Castaneda | HIATvsUC | -0.72583818 | -1.582452 | 0.130776 | 1 |
| Carmen Castaneda | HIAT-MIRTvsUC | 0.06639065 | -1.143553 | 1.276335 | 1 |
| Carmen Castaneda | HIRTvsUC | -0.2948086 | -0.868358 | 0.2787408 | 1 |
| Carmen Castaneda | LIRTvsUC | -0.24162834 | -1.464911 | 0.9816538 | 1 |
| Carmen Castaneda | MIRTvsUC | 0.38381751 | -0.6921663 | 1.459801 | 1 |
| Kenneth M Madden | ACvsUC | 1.2625382 | 0.0804822 | 2.444594 | 1 |
| Kenneth M Madden | HIATvsUC | -0.67298031 | -1.4959 | 0.1499389 | 1 |
| Kenneth M Madden | HIAT-MIRTvsUC | 0.06314414 | -1.096518 | 1.222806 | 1 |
| Kenneth M Madden | HIRTvsUC | -0.21458206 | -0.6963699 | 0.2672058 | 1 |
| Kenneth M Madden | LIRTvsUC | -0.24162833 | -1.406056 | 0.9227989 | 1 |
| Kenneth M Madden | MIRTvsUC | 0.31952782 | -0.7366722 | 1.375728 | 1 |
| Kenneth M Madden | ACvsUC | 1.1262443 | 0.048081 | 2.204407 | 1 |
| Kenneth M Madden | HIATvsUC | -0.6480199 | -1.407584 | 0.1115446 | 1 |
| Kenneth M Madden | HIAT-MIRTvsUC | 0.0564233 | -1.01416 | 1.127006 | 1 |
| Kenneth M Madden | HIRTvsUC | -0.20869601 | -0.6399823 | 0.2225903 | 1 |
| Kenneth M Madden | LIRTvsUC | -0.24162834 | -1.301486 | 0.8182297 | 1 |
| Kenneth M Madden | MIRTvsUC | 0.27391227 | -0.6993647 | 1.247189 | 1 |
| Maryam Nadi | ACvsUC | 1.4470497 | 0.4175711 | 2.476528 | 1 |
| Maryam Nadi | HIATvsUC | -0.70352873 | -1.503332 | 0.0962749 | 1 |
| Maryam Nadi | HIAT-MIRTvsUC | 0.08175009 | -1.051433 | 1.214933 | 1 |
| Maryam Nadi | HIRTvsUC | -0.2164738 | -0.6838182 | 0.2508705 | 1 |
| Maryam Nadi | LIRTvsUC | 0.41033165 | -0.5957285 | 1.416392 | 1 |
| R C Plotnikoff | ACvsUC | 1.4558253 | 0.326103 | 2.585548 | 1 |
| R C Plotnikoff | HIATvsUC | -0.70680364 | -1.584101 | 0.1704934 | 1 |
| R C Plotnikoff | HIAT-MIRTvsUC | 0.0758871 | -1.163459 | 1.315233 | 1 |
| R C Plotnikoff | HIRTvsUC | -0.22401434 | -0.811217 | 0.3631882 | 1 |
| R C Plotnikoff | LIRTvsUC | -0.24162834 | -1.49895 | 1.015693 | 1 |
| R C Plotnikoff | MIRTvsUC | 0.39298303 | -0.7094186 | 1.495385 | 1 |
| Vanessa Neves de Oliveira | ACvsUC | 1.6623422 | 0.6426068 | 2.682077 | 1 |
| Vanessa Neves de Oliveira | HIATvsUC | -0.96790491 | -1.776564 | -0.1592454 | 1 |
| Vanessa Neves de Oliveira | HIAT-MIRTvsUC | -0.20445668 | -0.4527181 | 0.0438048 | 1 |
| Vanessa Neves de Oliveira | HIRTvsUC | -0.24162834 | -0.9602968 | 0.4770401 | 1 |
| Vanessa Neves de Oliveira | LIRTvsUC | 1.3147998 | 0.1433279 | 2.486272 | 1 |
| Niloufar Ghadamyari | ACvsUC | 1.7243686 | 0.3912945 | 3.057443 | 1 |
| Niloufar Ghadamyari | HIATvsUC | -0.39508668 | -1.60544 | 0.8152665 | 1 |
| Niloufar Ghadamyari | HIAT-MIRTvsUC | 0.24907378 | -1.04771 | 1.545858 | 1 |
| Niloufar Ghadamyari | HIRTvsUC | -0.15980893 | -0.7175285 | 0.3979107 | 1 |
| Niloufar Ghadamyari | LIRTvsUC | -0.24162834 | -1.462749 | 0.9794928 | 1 |
| Niloufar Ghadamyari | MIRTvsUC | 0.60880053 | -0.6109463 | 1.828547 | 1 |

Supplementary Table23.Leave-one-out sensitivity analysis for HR in middle-aged and older adults with type 2 diabetes.

| **dropped_id** | **comparison** | **eff** | **lci** | **uci** | **connected** |
| --- | --- | --- | --- | --- | --- |
| Ping-Lun Hsieh | ACvsUC | 0.03459132 | -2.568418 | 2.6376 | 1 |
| Ping-Lun Hsieh | HIATvsUC | -0.64156087 | -2.721153 | 1.438031 | 1 |
| Ping-Lun Hsieh | HIRTvsUC | -0.04084399 | -1.312544 | 1.230856 | 1 |
| Ping-Lun Hsieh | LIRTvsUC | -0.09336953 | -2.31449 | 2.127751 | 1 |
| Theng Choon Ooi | ACvsUC | 0.10728463 | -2.458807 | 2.673376 | 1 |
| Theng Choon Ooi | HIATvsUC | -0.56807059 | -2.619545 | 1.483404 | 1 |
| Theng Choon Ooi | HIRTvsUC | 0.10466768 | -1.158921 | 1.368257 | 1 |
| Theng Choon Ooi | LIRTvsUC | -0.09336953 | -2.280681 | 2.093942 | 1 |
| Carmen Castaneda | ACvsUC | -0.08868339 | -2.424536 | 2.247169 | 1 |
| Carmen Castaneda | HIATvsUC | -0.75876832 | -2.63 | 1.112463 | 1 |
| Carmen Castaneda | HIRTvsUC | -0.27373076 | -1.418668 | 0.8712062 | 1 |
| Carmen Castaneda | LIRTvsUC | -0.09336953 | -2.076696 | 1.889957 | 1 |
| Kenneth M Madden | ACvsUC | -1.0940791 | -2.758862 | 0.5707039 | 1 |
| Kenneth M Madden | HIATvsUC | -0.6044443 | -1.794925 | 0.5860361 | 1 |
| Kenneth M Madden | HIRTvsUC | 0.02026419 | -0.5713347 | 0.6118631 | 1 |
| Kenneth M Madden | LIRTvsUC | -0.09336953 | -1.306019 | 1.11928 | 1 |
| Kenneth M Madden | ACvsUC | 1.2844679 | -0.4385301 | 3.007466 | 1 |
| Kenneth M Madden | HIATvsUC | -0.604892 | -1.795402 | 0.5856176 | 1 |
| Kenneth M Madden | HIRTvsUC | 0.02020693 | -0.5714066 | 0.6118205 | 1 |
| Kenneth M Madden | LIRTvsUC | -0.09336953 | -1.306031 | 1.119292 | 1 |
| Maryam Nadi | ACvsUC | 0.04554314 | -2.211167 | 2.302253 | 1 |
| Maryam Nadi | HIATvsUC | -0.62276996 | -2.423826 | 1.178286 | 1 |
| Maryam Nadi | HIRTvsUC | -0.00523995 | -0.9617782 | 0.9512982 | 1 |
| Niloufar Ghadamyari | disconnected |  |  |  | 0 |

Supplementary Table24.Meta-regression analysis of country for HbA1c in middle-aged and older adults with type 2 diabetes.

| **Intervention** | **Covariate** | **Coefficient** | **Standard Error** | **Z-statistic** | **P>z** | **lower confidence interval** | **upper confidence interval** |
| --- | --- | --- | --- | --- | --- | --- | --- |
| ACvsHIAT | country | -0.0969416 | 0.1542032 | -0.63 | 0.53 | -0.3991743 | 0.205291 |
|  | _cons | -0.0986312 | 0.8338379 | -0.12 | 0.906 | -1.732923 | 1.535661 |
| ACvsHIAT-LIRT | _cons | -0.962637 | 1.361613 | -0.71 | 0.48 | -3.631349 | 1.706075 |
| ACvsHIAT-MIRT | country | -0.0870477 | 0.1473135 | -0.59 | 0.555 | -0.3757768 | 0.2016815 |
|  | _cons | -0.3637545 | 0.7011033 | -0.52 | 0.604 | -1.737892 | 1.010383 |
| ACvsHIRT | country | -0.0717684 | 0.1431713 | -0.5 | 0.616 | -0.3523789 | 0.2088421 |
|  | _cons | -0.5513402 | 0.5084246 | -1.08 | 0.278 | -1.547834 | 0.4451537 |
| ACvsLIRT | country | -1.165709 | 0.568503 | -2.05 | 0.04 | -2.279954 | -0.0514635 |
|  | _cons | 8.597265 | 4.581745 | 1.88 | 0.061 | -0.3827899 | 17.57732 |
| ACvsMIAT | _cons | -0.9418252 | 0.8262001 | -1.14 | 0.254 | -2.561148 | 0.6774973 |
| ACvsMIRT | country | -0.0736171 | 0.124985 | -0.59 | 0.556 | -0.3185832 | 0.1713491 |
|  | _cons | -0.0326745 | 0.3666675 | -0.09 | 0.929 | -0.7513295 | 0.6859805 |
| ACvsUC | country | -0.0494792 | 0.1376123 | -0.36 | 0.719 | -0.3191943 | 0.2202359 |
|  | _cons | -0.0689663 | 0.4743111 | -0.15 | 0.884 | -0.9985988 | 0.8606663 |

Supplementary Table25.Meta-regression analysis of follow-up duration for HbA1c in middle-aged and older adults with type 2 diabetes.

| **Intervention** | **Covariate** | **Coefficient** | **Standard Error** | **Z-statistic** | **P>z** | **lower confidence interval** | **upper confidence interval** |
| --- | --- | --- | --- | --- | --- | --- | --- |
| ACvsHIAT | time | 0.0220206 | 0.1193413 | 0.18 | 0.854 | -0.2118839 | 0.2559252 |
|  | _cons | -0.5755213 | 0.8066121 | -0.71 | 0.476 | -2.156452 | 1.005409 |
| ACvsHIAT-LIRT | _cons | -0.4263355 | 0.5735891 | -0.74 | 0.457 | -1.550549 | 0.6978785 |
| ACvsHIAT-MIRT | time | 0.0754416 | 0.1065828 | 0.71 | 0.479 | -0.1334569 | 0.2843402 |
|  | _cons | -1.042046 | 0.6627466 | -1.57 | 0.116 | -2.341006 | 0.2569134 |
| ACvsHIRT | time | -0.1409578 | 0.1828859 | -0.77 | 0.441 | -0.4994077 | 0.217492 |
|  | _cons | -0.1983763 | 0.7581484 | -0.26 | 0.794 | -1.68432 | 1.287567 |
| ACvsLIRT | _cons | -0.6918923 | 0.408969 | -1.69 | 0.091 | -1.493457 | 0.1096721 |
| ACvsMIAT | time | 0.0532599 | 0.2336637 | 0.23 | 0.82 | -0.4047126 | 0.5112324 |
|  | _cons | -0.9124776 | 0.9934077 | -0.92 | 0.358 | -2.859521 | 1.034566 |
| ACvsMIRT | time | 0.0509576 | 0.0566474 | 0.9 | 0.368 | -0.0600692 | 0.1619845 |
|  | _cons | -0.5012322 | 0.3561321 | -1.41 | 0.159 | -1.199238 | 0.1967739 |
| ACvsUC | time | 0.0021979 | 0.0770789 | 0.03 | 0.977 | -0.1488739 | 0.1532697 |
|  | _cons | -0.1461453 | 0.4848588 | -0.3 | 0.763 | -1.096451 | 0.8041606 |

Supplementary Table26.Meta-regression analysis of mean age for HbA1c in middle-aged and older adults with type 2 diabetes.

| **Intervention** | **Covariate** | **Coefficient** | **Standard Error** | **Z-statistic** | **P>z** | **lower confidence interval** | **upper confidence interval** |
| --- | --- | --- | --- | --- | --- | --- | --- |
| ACvsHIAT | ma | -0.1211841 | 0.2686822 | -0.45 | 0.652 | -0.6477917 | 0.4054234 |
|  | _cons | 6.081063 | 15.53952 | 0.39 | 0.696 | -24.37583 | 36.53796 |
| ACvsHIAT-LIRT | _cons | -0.5427407 | 0.5165139 | -1.05 | 0.293 | -1.555089 | 0.469608 |
| ACvsHIAT-MIRT | ma | -0.1428529 | 0.175778 | -0.81 | 0.416 | -0.4873714 | 0.2016656 |
|  | _cons | 7.167236 | 10.33012 | 0.69 | 0.488 | -13.07943 | 27.41391 |
| ACvsHIRT | ma | 0.0157474 | 0.0776512 | 0.2 | 0.839 | -0.1364462 | 0.1679411 |
|  | _cons | -1.843323 | 5.144223 | -0.36 | 0.72 | -11.92581 | 8.239168 |
| ACvsLIRT | ma | 0.526002 | 0.3317528 | 1.59 | 0.113 | -0.1242215 | 1.176226 |
|  | _cons | -30.57681 | 18.76977 | -1.63 | 0.103 | -67.36488 | 6.211271 |
| ACvsMIAT | ma | 0.0438065 | 0.0838891 | 0.52 | 0.602 | -0.120613 | 0.2082261 |
|  | _cons | -3.503985 | 5.403857 | -0.65 | 0.517 | -14.09535 | 7.087381 |
| ACvsMIRT | ma | 0.0430142 | 0.0604085 | 0.71 | 0.476 | -0.0753843 | 0.1614127 |
|  | _cons | -3.068336 | 4.044089 | -0.76 | 0.448 | -10.9946 | 4.857932 |
| ACvsUC | ma | 0.0375894 | 0.0687025 | 0.55 | 0.584 | -0.0970651 | 0.1722439 |
|  | _cons | -2.629706 | 4.565328 | -0.58 | 0.565 | -11.57758 | 6.318172 |

Supplementary Table27.Meta-regression analysis of country for FPG in middle-aged and older adults with type 2 diabetes.

| **Intervention** | **Covariate** | **Coefficient** | **Standard Error** | **Z-statistic** | **P>z** | **lower confidence interval** | **upper confidence interval** |
| --- | --- | --- | --- | --- | --- | --- | --- |
| ACvsHIAT | country | 1.161955 | 7.345784 | 0.16 | 0.874 | -13.23552 | 15.55943 |
|  | _cons | -21.51429 | 49.38812 | -0.44 | 0.663 | -118.3132 | 75.28466 |
| ACvsHIAT-MIRT | country | 0.6418689 | 7.554533 | 0.08 | 0.932 | -14.16474 | 15.44848 |
|  | _cons | -12.37311 | 49.46957 | -0.25 | 0.802 | -109.3317 | 84.58548 |
| ACvsHIRT | country | 13.10652 | 8.728656 | 1.5 | 0.133 | -4.001337 | 30.21437 |
|  | _cons | -80.85788 | 52.16818 | -1.55 | 0.121 | -183.1056 | 21.38987 |
| ACvsLIRT | country | -18.97657 | 23.29325 | -0.81 | 0.415 | -64.6305 | 26.67735 |
|  | _cons | 188.7573 | 196.017 | 0.96 | 0.336 | -195.429 | 572.9435 |
| ACvsMIAT | _cons | 10.67216 | 23.57616 | 0.45 | 0.651 | -35.53625 | 56.88058 |
| ACvsMIRT | country | -0.4130904 | 4.569427 | -0.09 | 0.928 | -9.369002 | 8.542821 |
|  | _cons | -15.29432 | 17.45291 | -0.88 | 0.381 | -49.50139 | 18.91276 |
| ACvsUC | country | 9.75426 | 7.448216 | 1.31 | 0.19 | -4.843975 | 24.3525 |
|  | _cons | -53.56851 | 47.66734 | -1.12 | 0.261 | -146.9948 | 39.85776 |

Supplementary Table28.Meta-regression analysis of follow-up duration for FPG in middle-aged and older adults with type 2 diabetes.

| **Intervention** | **Covariate** | **Coefficient** | **Standard Error** | **Z-statistic** | **P>z** | **lower confidence interval** | **upper confidence interval** |
| --- | --- | --- | --- | --- | --- | --- | --- |
| ACvsHIAT | _cons | -11.71317 | 10.99306 | -1.07 | 0.287 | -33.25918 | 9.832844 |
| ACvsHIAT-MIRT | _cons | -8.23999 | 16.13568 | -0.51 | 0.61 | -39.86535 | 23.38537 |
| ACvsHIRT | time | 2.703524 | 197.9878 | 0.01 | 0.989 | -385.3455 | 390.7526 |
|  | _cons | -16.10654 | 594.7561 | -0.03 | 0.978 | -1181.807 | 1149.594 |
| ACvsLIRT | _cons | 12.57248 | 19.47004 | 0.65 | 0.518 | -25.5881 | 50.73307 |
| ACvsMIAT | time | -10.1407 | 198.1289 | -0.05 | 0.959 | -398.4662 | 378.1848 |
|  | _cons | 33.47665 | 595.7302 | 0.06 | 0.955 | -1134.133 | 1201.086 |
| ACvsMIRT | _cons | -16.56093 | 11.51173 | -1.44 | 0.15 | -39.1235 | 6.001638 |
|  | time | -3.44115 | 197.9389 | -0.02 | 0.986 | -391.3944 | 384.5121 |
| ACvsUC | _cons | 24.8671 | 594.4125 | 0.04 | 0.967 | -1140.16 | 1189.894 |

Supplementary Table29.Meta-regression analysis of mean age for FPG in middle-aged and older adults with type 2 diabetes.

| **Intervention** | **Covariate** | **Coefficient** | **Standard Error** | **Z-statistic** | **P>z** | **lower confidence interval** | **upper confidence interval** |
| --- | --- | --- | --- | --- | --- | --- | --- |
| ACvsHIAT | ma | 1.363957 | 2.550318 | 0.53 | 0.593 | -3.634575 | 6.362489 |
|  | _cons | -111.5105 | 176.2691 | -0.63 | 0.527 | -456.9917 | 233.9706 |
| ACvsHIAT-MIRT | ma | -7.314313 | 8.749931 | -0.84 | 0.403 | -24.46386 | 9.835237 |
|  | _cons | 381.2355 | 507.6415 | 0.75 | 0.453 | -613.7236 | 1376.194 |
| ACvsHIRT | ma | 1.260412 | 3.88963 | 0.32 | 0.746 | -6.363123 | 8.883946 |
|  | _cons | -88.44133 | 250.9001 | -0.35 | 0.724 | -580.1965 | 403.3138 |
| ACvsLIRT | ma | 36.96908 | 23.67169 | 1.56 | 0.118 | -9.426578 | 83.36473 |
|  | _cons | -2067.044 | 1316.698 | -1.57 | 0.116 | -4647.724 | 513.6366 |
| ACvsMIAT | ma | 0.7796311 | 4.235997 | 0.18 | 0.854 | -7.52277 | 9.082032 |
|  | _cons | -61.43457 | 268.6538 | -0.23 | 0.819 | -587.9863 | 465.1172 |
| ACvsMIRT | ma | 4.127289 | 3.010741 | 1.37 | 0.17 | -1.773655 | 10.02823 |
|  | _cons | -282.0517 | 196.2208 | -1.44 | 0.151 | -666.6373 | 102.534 |
| ACvsUC | ma | 2.822016 | 3.79817 | 0.74 | 0.457 | -4.62226 | 10.26629 |
|  | _cons | -172.8104 | 242.4198 | -0.71 | 0.476 | -647.9444 | 302.3236 |

Supplementary Table30.Meta-regression analysis of country for VO_2_peak in middle-aged and older adults with type 2 diabetes.

| **Intervention** | **Covariate** | **Coefficient** | **Standard Error** | **Z-statistic** | **P>z** | **lower confidence interval** | **upper confidence interval** |
| --- | --- | --- | --- | --- | --- | --- | --- |
| ACvsHIAT | country | 0.0658671 | 8.738032 | 0.01 | 0.994 | -17.06036 | 17.1921 |
|  | _cons | 1.9219 | 10.404 | 0.18 | 0.853 | -18.46956 | 22.31336 |
| ACvsHIAT-HIRT | _cons | 2.539464 | 34.63229 | 0.07 | 0.942 | -65.33859 | 70.41751 |
| ACvsHIAT-MIRT | country | -1.204273 | 8.735651 | -0.14 | 0.89 | -18.32583 | 15.91729 |
|  | _cons | 5.570624 | 10.42668 | 0.53 | 0.593 | -14.8653 | 26.00655 |
| ACvsHIRT | country | -0.6999989 | 8.74575 | -0.08 | 0.936 | -17.84135 | 16.44136 |
|  | _cons | 2.73953 | 10.72593 | 0.26 | 0.798 | -18.28291 | 23.76197 |
| ACvsMIRT | country | 0.0444128 | 8.696279 | 0.01 | 0.996 | -16.99998 | 17.08881 |
|  | _cons | 0.4151812 | 8.858615 | 0.05 | 0.963 | -16.94739 | 17.77775 |
| ACvsUC | country | -1.052695 | 8.737469 | -0.12 | 0.904 | -18.17782 | 16.07243 |
|  | _cons | 3.445158 | 10.52347 | 0.33 | 0.743 | -17.18047 | 24.07078 |

Supplementary Table31.Meta-regression analysis of follow-up duration for VO_2_peak in middle-aged and older adults with type 2 diabetes.

| **Intervention** | **Covariate** | **Coefficient** | **Standard Error** | **Z-statistic** | **P>z** | **lower confidence interval** | **upper confidence interval** |
| --- | --- | --- | --- | --- | --- | --- | --- |
| ACvsHIAT | time | -0.0949965 | 17.62598 | -0.01 | 0.996 | -34.64129 | 34.45129 |
|  | _cons | 3.17833 | 52.91827 | 0.06 | 0.952 | -100.5396 | 106.8962 |
| ACvsHIAT-HIRT | _cons | 3.239292 | 17.79946 | 0.18 | 0.856 | -31.647 | 38.12559 |
| ACvsHIAT-MIRT | time | 0.4110255 | 17.62643 | 0.02 | 0.981 | -34.13614 | 34.95819 |
|  | _cons | -1.05161 | 52.9179 | -0.02 | 0.984 | -104.7688 | 102.6656 |
| ACvsHIRT | time | -1.035683 | 17.72465 | -0.06 | 0.953 | -35.77535 | 33.70398 |
|  | _cons | 2.010812 | 53.11964 | 0.04 | 0.97 | -102.1018 | 106.1234 |
| ACvsMIRT | time | 0.0731571 | 17.62748 | 0 | 0.997 | -34.47607 | 34.62238 |
|  | _cons | 0.2392054 | 52.89709 | 0 | 0.996 | -103.4372 | 103.9156 |
| ACvsUC | time | 0.242247 | 17.62563 | 0.01 | 0.989 | -34.30336 | 34.78785 |
|  | _cons | -1.823067 | 52.90847 | -0.03 | 0.973 | -105.5218 | 101.8756 |

Supplementary Table32.Meta-regression analysis of mean age for VO_2_peak in middle-aged and older adults with type 2 diabetes.

| **Intervention** | **Covariate** | **Coefficient** | **Standard Error** | **Z-statistic** | **P>z** | **lower confidence interval** | **upper confidence interval** |
| --- | --- | --- | --- | --- | --- | --- | --- |
| ACvsHIAT | ma | -0.249824 | 5.553392 | -0.04 | 0.964 | -11.13427 | 10.63462 |
|  | _cons | 16.25172 | 339.9413 | 0.05 | 0.962 | -650.0211 | 682.5245 |
| ACvsHIAT-HIRT | _cons | 2.983861 | 34.69036 | 0.09 | 0.931 | -65.00799 | 70.97572 |
| ACvsHIAT-MIRT | ma | 0.8095561 | 5.545975 | 0.15 | 0.884 | -10.06035 | 11.67947 |
|  | _cons | -45.02118 | 339.5613 | -0.13 | 0.895 | -710.5491 | 620.5067 |
| ACvsHIRT | ma | 0.3456111 | 5.5214 | 0.06 | 0.95 | -10.47613 | 11.16736 |
|  | _cons | -19.32463 | 338.106 | -0.06 | 0.954 | -682.0002 | 643.351 |
| ACvsMIRT | ma | 0.0817244 | 5.519304 | 0.01 | 0.988 | -10.73591 | 10.89936 |
|  | _cons | -4.551863 | 338.2969 | -0.01 | 0.989 | -667.6017 | 658.4979 |
| ACvsUC | ma | 0.4200507 | 5.52317 | 0.08 | 0.939 | -10.40516 | 11.24527 |
|  | _cons | -24.62435 | 338.2277 | -0.07 | 0.942 | -687.5384 | 638.2897 |

Supplementary Table33.Meta-regression analysis of country for SBP in middle-aged and older adults with type 2 diabetes.

| **Intervention** | **Covariate** | **Coefficient** | **Standard Error** | **Z-statistic** | **P>z** | **lower confidence interval** | **upper confidence interval** |
| --- | --- | --- | --- | --- | --- | --- | --- |
| ACvsHIAT | country | 6.061571 | 86.8118 | 0.07 | 0.944 | -164.0864 | 176.2096 |
|  | _cons | -39.81629 | 434.0605 | -0.09 | 0.927 | -890.5593 | 810.9267 |
| ACvsHIAT-MIRT | _cons | 11.17874 | 260.4111 | 0.04 | 0.966 | -499.2176 | 521.5751 |
| ACvsHIRT | country | -1.043101 | 86.75951 | -0.01 | 0.99 | -171.0886 | 169.0024 |
|  | _cons | 10.90308 | 433.9756 | 0.03 | 0.98 | -839.6736 | 861.4797 |
| ACvsLIRT | _cons | 4.910761 | 87.27606 | 0.06 | 0.955 | -166.1472 | 175.9687 |
| ACvsMIRT | country | 1.954106 | 37.20897 | 0.05 | 0.958 | -70.97413 | 74.88234 |
|  | _cons | -6.954106 | 37.49404 | -0.19 | 0.853 | -80.44107 | 66.53285 |
| ACvsUC | country | -0.9144241 | 86.75687 | -0.01 | 0.992 | -170.9548 | 169.1259 |
|  | _cons | 14.39776 | 433.9599 | 0.03 | 0.974 | -836.148 | 864.9436 |

Supplementary Table34.Meta-regression analysis of follow-up duration for SBP in middle-aged and older adults with type 2 diabetes.

| **Intervention** | **Covariate** | **Coefficient** | **Standard Error** | **Z-statistic** | **P>z** | **lower confidence interval** | **upper confidence interval** |
| --- | --- | --- | --- | --- | --- | --- | --- |
| ACvsHIAT | time | 16.60319 | 109.3589 | 0.15 | 0.879 | -197.7364 | 230.9427 |
|  | _cons | -59.27639 | 328.0772 | -0.18 | 0.857 | -702.296 | 583.7432 |
| ACvsHIAT-MIRT | _cons | -5.631204 | 6.000199 | -0.94 | 0.348 | -17.39138 | 6.12897 |
| ACvsHIRT | time | 3.775936 | 108.9541 | 0.03 | 0.972 | -209.7702 | 217.3221 |
|  | _cons | -26.02168 | 327.0561 | -0.08 | 0.937 | -667.0399 | 614.9966 |
| ACvsLIRT | _cons | -13.72333 | 8.525748 | -1.61 | 0.107 | -30.43349 | 2.986828 |
| ACvsMIRT | _cons | -6.075014 | 3.550628 | -1.71 | 0.087 | -13.03412 | 0.8840899 |
| ACvsUC | time | 3.148339 | 108.9503 | 0.03 | 0.977 | -210.3903 | 216.687 |
|  | _cons | -19.16912 | 327.0146 | -0.06 | 0.953 | -660.1059 | 621.7676 |

Supplementary Table35.Meta-regression analysis of mean age for SBP in middle-aged and older adults with type 2 diabetes.

| **Intervention** | **Covariate** | **Coefficient** | **Standard Error** | **Z-statistic** | **P>z** | **lower confidence interval** | **upper confidence interval** |
| --- | --- | --- | --- | --- | --- | --- | --- |
| ACvsHIAT | ma | 0.4549787 | 0.7236819 | 0.63 | 0.53 | -0.9634118 | 1.873369 |
|  | _cons | -41.53107 | 50.94008 | -0.82 | 0.415 | -141.3718 | 58.30964 |
| ACvsHIAT-MIRT | _cons | -13.75491 | 12.88251 | -1.07 | 0.286 | -39.00417 | 11.49435 |
| ACvsHIRT | ma | 13.62015 | 8.217493 | 1.66 | 0.097 | -2.485838 | 29.72614 |
|  | _cons | -771.0269 | 458.6162 | -1.68 | 0.093 | -1669.898 | 127.8444 |
| ACvsLIRT | _cons | -21.56599 | 14.18561 | -1.52 | 0.128 | -49.36928 | 6.237291 |
| ACvsMIRT | ma | 1.786494 | 2.204465 | 0.81 | 0.418 | -2.534178 | 6.107165 |
|  | _cons | -114.5121 | 133.9976 | -0.85 | 0.393 | -377.1425 | 148.1183 |
| ACvsUC | ma | 13.56753 | 8.167584 | 1.66 | 0.097 | -2.440646 | 29.5757 |
|  | _cons | -763.7812 | 455.3534 | -1.68 | 0.093 | -1656.258 | 128.6951 |

Supplementary Table36.Meta-regression analysis of country for HR in middle-aged and older adults with type 2 diabetes.

| **Intervention** | **Covariate** | **Coefficient** | **Standard Error** | **Z-statistic** | **P>z** | **lower confidence interval** | **upper confidence interval** |
| --- | --- | --- | --- | --- | --- | --- | --- |
| ACvsHIAT | country | -3.303091 | 170.3325 | -0.02 | 0.985 | -337.1487 | 330.5425 |
|  | _cons | 11.86107 | 681.3615 | 0.02 | 0.986 | -1323.583 | 1347.305 |
| ACvsHIRT | country | -0.2189088 | 78.65093 | 0 | 0.998 | -154.3719 | 153.9341 |
|  | _cons | 0.3373287 | 248.035 | 0 | 0.999 | -485.8023 | 486.477 |
| ACvsLIRT | _cons | 0.9958706 | 170.3389 | 0.01 | 0.995 | -332.8623 | 334.854 |
| ACvsUC | country | 0.7534525 | 78.64682 | 0.01 | 0.992 | -153.3915 | 154.8984 |
|  | _cons | -1.771214 | 248.0297 | -0.01 | 0.994 | -487.9006 | 484.3581 |

Supplementary Table37.Meta-regression analysis of follow-up duration for HR in middle-aged and older adults with type 2 diabetes.

| **Intervention** | **Covariate** | **Coefficient** | **Standard Error** | **Z-statistic** | **P>z** | **lower confidence interval** | **upper confidence interval** |
| --- | --- | --- | --- | --- | --- | --- | --- |
| ACvsHIAT | time | 2.45955 | 225.1088 | 0.01 | 0.991 | -438.7456 | 443.6647 |
|  | _cons | -8.730177 | 675.3113 | -0.01 | 0.99 | -1332.316 | 1314.856 |
| ACvsHIAT | time | 0.3358761 | 121.3746 | 0 | 0.998 | -237.554 | 238.2258 |
|  | _cons | -1.16291 | 462.0195 | 0 | 0.998 | -906.7044 | 904.3786 |
| ACvsLIRT | _cons | 0.5178382 | 116.1215 | 0 | 0.996 | -227.0761 | 228.1118 |
| ACvsUC | time | -1.791135 | 121.3719 | -0.01 | 0.988 | -239.6758 | 236.0935 |
|  | _cons | 6.891358 | 462.0075 | 0.01 | 0.988 | -898.6267 | 912.4094 |

Supplementary Table38.Meta-regression analysis of mean age for HR in middle-aged and older adults with type 2 diabetes.

| **Intervention** | **Covariate** | **Coefficient** | **Standard Error** | **Z-statistic** | **P>z** | **lower confidence interval** | **upper confidence interval** |
| --- | --- | --- | --- | --- | --- | --- | --- |
| ACvsHIAT | ma | -2.13359 | 0.4036974 | -5.29 | 0 | -2.924822 | -1.342357 |
|  | _cons | 148.8545 | 28.42968 | 5.24 | 0 | 93.13331 | 204.5756 |
| ACvsHIRT | ma | -3.104978 | 7.804577 | -0.4 | 0.691 | -18.40167 | 12.19171 |
|  | _cons | 205.844 | 437.7217 | 0.47 | 0.638 | -652.0748 | 1063.763 |
| ACvsLIRT | _cons | 39.81942 | 11.38109 | 3.5 | 0 | 17.5129 | 62.12595 |
| ACvsUC | ma | -3.786142 | 7.803776 | -0.49 | 0.628 | -19.08126 | 11.50898 |
|  | _cons | 249.0603 | 437.6605 | 0.57 | 0.569 | -608.7385 | 1106.859 |


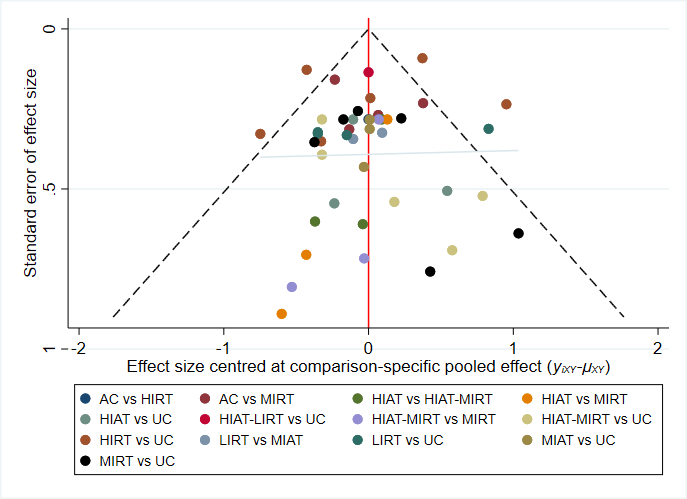


Supplementary Figure1.Comparison-adjusted funnel plot for the HbA1c outcome in middle-aged and older adults with type 2 diabetes.


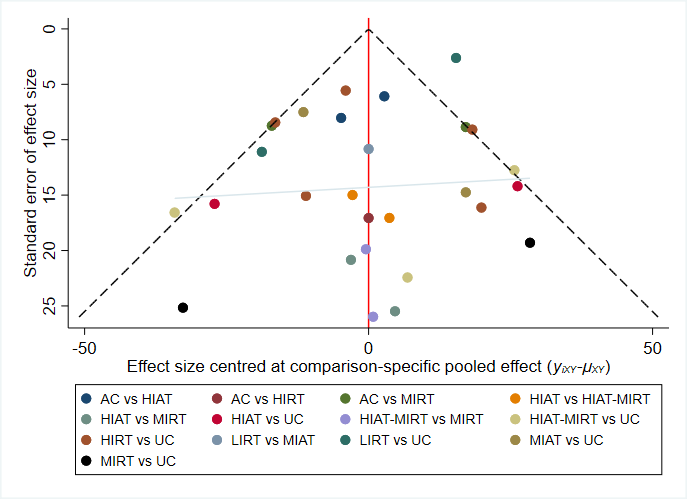


Supplementary Figure2.Comparison-adjusted funnel plot for the FPG outcome in middle-aged and older adults with type 2 diabetes.


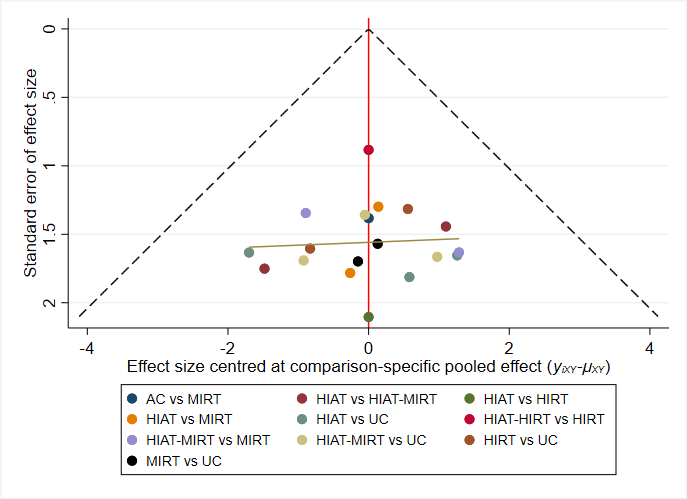


Supplementary Figure3.Comparison-adjusted funnel plot for the VO_2_peak outcome in middle-aged and older adults with type 2 diabetes.


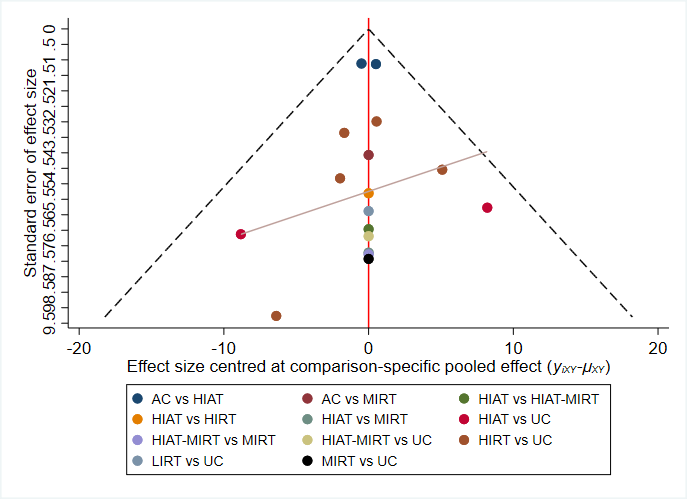


Supplementary Figure4.Comparison-adjusted funnel plot for the SBP outcome in middle-aged and older adults with type 2 diabetes.


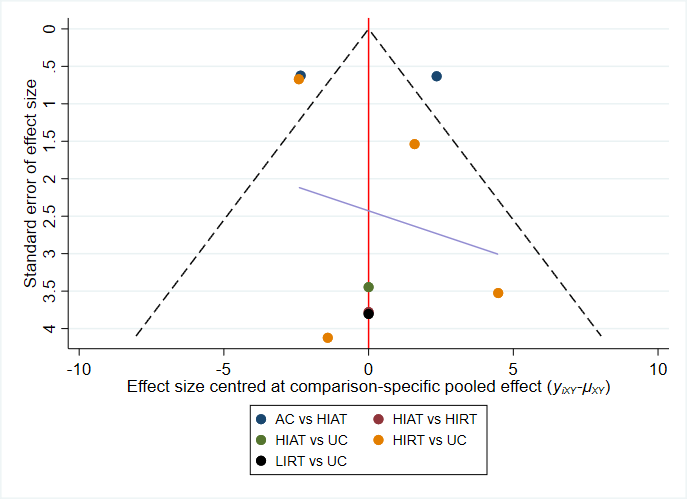


Supplementary Figure5.Comparison-adjusted funnel plot for the HR outcome in middle-aged and older adults with type 2 diabetes.

Supplementary Table39.GRADE certainty of evidence for the HbA1c outcome in middle-aged and older adults with type 2 diabetes.

| **Comparison** | **Number of studies** | **Within-study bias** | **Reporting bias** | **Indirectness** | **Imprecision** | **Heterogeneity** | **Incoherence** | **Confidence rating** | **Reason(s) for downgrading** |
| --- | --- | --- | --- | --- | --- | --- | --- | --- | --- |
| AC:HIRT | 1 | No concerns | Low risk | No concerns | No concerns | Some concerns | No concerns | Moderate | ["Heterogeneity"] |
| AC:MIRT | 4 | No concerns | Low risk | No concerns | Some concerns | Some concerns | No concerns | Low | ["Imprecision","Heterogeneity"] |
| HIAT:HIAT-MIRT | 3 | No concerns | Low risk | No concerns | Some concerns | No concerns | No concerns | Moderate | ["Imprecision"] |
| HIAT:MIRT | 3 | No concerns | Low risk | No concerns | Some concerns | No concerns | No concerns | Moderate | ["Imprecision"] |
| HIAT:UC | 3 | No concerns | Low risk | No concerns | Some concerns | Some concerns | No concerns | Low | ["Imprecision","Heterogeneity"] |
| HIAT-LIRT:UC | 1 | No concerns | Low risk | No concerns | Some concerns | No concerns | No concerns | Moderate | ["Imprecision"] |
| HIAT-MIRT:MIRT | 3 | No concerns | Low risk | No concerns | Some concerns | Some concerns | No concerns | Low | ["Imprecision","Heterogeneity"] |
| HIAT-MIRT:UC | 5 | No concerns | Low risk | No concerns | Some concerns | Some concerns | No concerns | Low | ["Imprecision","Heterogeneity"] |
| HIRT:UC | 6 | No concerns | Low risk | No concerns | No concerns | Some concerns | No concerns | Moderate | ["Heterogeneity"] |
| LIRT:MIAT | 2 | No concerns | Low risk | No concerns | Some concerns | No concerns | No concerns | Moderate | ["Imprecision"] |
| LIRT:UC | 4 | No concerns | Low risk | No concerns | Some concerns | Some concerns | No concerns | Low | ["Imprecision","Heterogeneity"] |
| MIAT:UC | 3 | No concerns | Low risk | No concerns | Some concerns | Some concerns | No concerns | Low | ["Imprecision","Heterogeneity"] |
| MIRT:UC | 6 | No concerns | Low risk | No concerns | Some concerns | Some concerns | No concerns | Low | ["Imprecision","Heterogeneity"] |
| AC:HIAT | 0 | No concerns | Low risk | No concerns | Some concerns | Some concerns | No concerns | Low | ["Imprecision","Heterogeneity"] |
| AC:HIAT-LIRT | 0 | No concerns | Low risk | No concerns | Some concerns | No concerns | No concerns | Moderate | ["Imprecision"] |
| AC:HIAT-MIRT | 0 | No concerns | Low risk | No concerns | Some concerns | Some concerns | No concerns | Low | ["Imprecision","Heterogeneity"] |
| AC:LIRT | 0 | No concerns | Low risk | No concerns | Some concerns | Some concerns | No concerns | Low | ["Imprecision","Heterogeneity"] |
| AC:MIAT | 0 | No concerns | Low risk | No concerns | Some concerns | Some concerns | No concerns | Low | ["Imprecision","Heterogeneity"] |
| AC:UC | 0 | No concerns | Low risk | No concerns | Some concerns | No concerns | No concerns | Moderate | ["Imprecision"] |
| HIAT:HIAT-LIRT | 0 | No concerns | Low risk | No concerns | Some concerns | No concerns | No concerns | Moderate | ["Imprecision"] |
| HIAT:HIRT | 0 | No concerns | Low risk | No concerns | Some concerns | Some concerns | No concerns | Low | ["Imprecision","Heterogeneity"] |
| HIAT:LIRT | 0 | No concerns | Low risk | No concerns | Some concerns | No concerns | No concerns | Moderate | ["Imprecision"] |
| HIAT:MIAT | 0 | No concerns | Low risk | No concerns | Some concerns | No concerns | No concerns | Moderate | ["Imprecision"] |
| HIAT-LIRT:HIAT-MIRT | 0 | No concerns | Low risk | No concerns | Some concerns | No concerns | No concerns | Moderate | ["Imprecision"] |
| HIAT-LIRT:HIRT | 0 | No concerns | Low risk | No concerns | Some concerns | No concerns | No concerns | Moderate | ["Imprecision"] |
| HIAT-LIRT:LIRT | 0 | No concerns | Low risk | No concerns | Some concerns | No concerns | No concerns | Moderate | ["Imprecision"] |
| HIAT-LIRT:MIAT | 0 | No concerns | Low risk | No concerns | Some concerns | No concerns | No concerns | Moderate | ["Imprecision"] |
| HIAT-LIRT:MIRT | 0 | No concerns | Low risk | No concerns | Some concerns | No concerns | No concerns | Moderate | ["Imprecision"] |
| HIAT-MIRT:HIRT | 0 | No concerns | Low risk | No concerns | Some concerns | Some concerns | No concerns | Low | ["Imprecision","Heterogeneity"] |
| HIAT-MIRT:LIRT | 0 | No concerns | Low risk | No concerns | Some concerns | No concerns | No concerns | Moderate | ["Imprecision"] |
| HIAT-MIRT:MIAT | 0 | No concerns | Low risk | No concerns | Some concerns | No concerns | No concerns | Moderate | ["Imprecision"] |
| HIRT:LIRT | 0 | No concerns | Low risk | No concerns | Some concerns | Some concerns | No concerns | Low | ["Imprecision","Heterogeneity"] |
| HIRT:MIAT | 0 | No concerns | Low risk | No concerns | Some concerns | Some concerns | No concerns | Low | ["Imprecision","Heterogeneity"] |
| HIRT:MIRT | 0 | No concerns | Low risk | No concerns | No concerns | Some concerns | No concerns | Moderate | ["Heterogeneity"] |
| LIRT:MIRT | 0 | No concerns | Low risk | No concerns | Some concerns | Some concerns | No concerns | Low | ["Imprecision","Heterogeneity"] |
| MIAT:MIRT | 0 | No concerns | Low risk | No concerns | Some concerns | Some concerns | No concerns | Low | ["Imprecision","Heterogeneity"] |

Supplementary Table40.GRADE certainty of evidence for the FPG outcome in middle-aged and older adults with type 2 diabetes.

| **Comparison** | **Number of studies** | **Within-study bias** | **Reporting bias** | **Indirectness** | **Imprecision** | **Heterogeneity** | **Incoherence** | **Confidence rating** | **Reason(s) for downgrading** |
| --- | --- | --- | --- | --- | --- | --- | --- | --- | --- |
| AC:HIAT | 2 | No concerns | Low risk | No concerns | No concerns | Some concerns | Some concerns | Low | ["Heterogeneity","Incoherence"] |
| AC:HIRT | 1 | No concerns | Low risk | No concerns | Some concerns | Some concerns | No concerns | Low | ["Imprecision","Heterogeneity"] |
| AC:MIRT | 2 | No concerns | Low risk | No concerns | No concerns | Some concerns | Some concerns | Low | ["Heterogeneity","Incoherence"] |
| HIAT:HIAT-MIRT | 2 | No concerns | Low risk | No concerns | Some concerns | Some concerns | Some concerns | Very low | ["Imprecision","Heterogeneity","Incoherence"] |
| HIAT:MIRT | 2 | No concerns | Low risk | No concerns | Some concerns | Some concerns | Some concerns | Very low | ["Imprecision","Heterogeneity","Incoherence"] |
| HIAT:UC | 2 | No concerns | Low risk | No concerns | No concerns | Some concerns | Some concerns | Low | ["Heterogeneity","Incoherence"] |
| HIAT-MIRT:MIRT | 2 | No concerns | Low risk | No concerns | Some concerns | No concerns | No concerns | Moderate | ["Imprecision"] |
| HIAT-MIRT:UC | 3 | No concerns | Low risk | No concerns | Some concerns | Some concerns | No concerns | Low | ["Imprecision","Heterogeneity"] |
| HIRT:UC | 5 | No concerns | Low risk | No concerns | Some concerns | Some concerns | No concerns | Low | ["Imprecision","Heterogeneity"] |
| LIRT:MIAT | 1 | No concerns | Low risk | No concerns | Some concerns | No concerns | No concerns | Moderate | ["Imprecision"] |
| LIRT:UC | 2 | No concerns | Low risk | No concerns | Some concerns | Some concerns | No concerns | Low | ["Imprecision","Heterogeneity"] |
| MIAT:UC | 2 | No concerns | Low risk | No concerns | Some concerns | Some concerns | Some concerns | Very low | ["Imprecision","Heterogeneity","Incoherence"] |
| MIRT:UC | 2 | No concerns | Low risk | No concerns | Some concerns | No concerns | No concerns | Moderate | ["Imprecision"] |
| AC:HIAT-MIRT | 0 | No concerns | Low risk | No concerns | Some concerns | Some concerns | No concerns | Low | ["Imprecision","Heterogeneity"] |
| AC:LIRT | 0 | No concerns | Low risk | No concerns | Some concerns | Some concerns | Some concerns | Very low | ["Imprecision","Heterogeneity","Incoherence"] |
| AC:MIAT | 0 | No concerns | Low risk | No concerns | Some concerns | Some concerns | Some concerns | Very low | ["Imprecision","Heterogeneity","Incoherence"] |
| AC:UC | 0 | No concerns | Low risk | No concerns | Some concerns | Some concerns | Some concerns | Very low | ["Imprecision","Heterogeneity","Incoherence"] |
| HIAT:HIRT | 0 | No concerns | Low risk | No concerns | Some concerns | Some concerns | Some concerns | Very low | ["Imprecision","Heterogeneity","Incoherence"] |
| HIAT:LIRT | 0 | No concerns | Low risk | No concerns | Some concerns | No concerns | Some concerns | Low | ["Imprecision","Incoherence"] |
| HIAT:MIAT | 0 | No concerns | Low risk | No concerns | Some concerns | No concerns | Some concerns | Low | ["Imprecision","Incoherence"] |
| HIAT-MIRT:HIRT | 0 | No concerns | Low risk | No concerns | Some concerns | No concerns | Some concerns | Low | ["Imprecision","Incoherence"] |
| HIAT-MIRT:LIRT | 0 | No concerns | Low risk | No concerns | Some concerns | No concerns | Some concerns | Low | ["Imprecision","Incoherence"] |
| HIAT-MIRT:MIAT | 0 | No concerns | Low risk | No concerns | Some concerns | No concerns | No concerns | Moderate | ["Imprecision"] |
| HIRT:LIRT | 0 | No concerns | Low risk | No concerns | Some concerns | No concerns | No concerns | Moderate | ["Imprecision"] |
| HIRT:MIAT | 0 | No concerns | Low risk | No concerns | Some concerns | No concerns | Some concerns | Low | ["Imprecision","Incoherence"] |
| HIRT:MIRT | 0 | No concerns | Low risk | No concerns | Some concerns | No concerns | Some concerns | Low | ["Imprecision","Incoherence"] |
| LIRT:MIRT | 0 | No concerns | Low risk | No concerns | Some concerns | No concerns | Some concerns | Low | ["Imprecision","Incoherence"] |
| MIAT:MIRT | 0 | No concerns | Low risk | No concerns | Some concerns | No concerns | Some concerns | Low | ["Imprecision","Incoherence"] |

Supplementary Table41.GRADE certainty of evidence for the VO_2_peak outcome in middle-aged and older adults with type 2 diabetes.

| **Comparison** | **Number of studies** | **Within-study bias** | **Reporting bias** | **Indirectness** | **Imprecision** | **Heterogeneity** | **Incoherence** | **Confidence rating** | **Reason(s) for downgrading** |
| --- | --- | --- | --- | --- | --- | --- | --- | --- | --- |
| HIAT:UC | 3 | No concerns | Low risk | No concerns | No concerns | No concerns | No concerns | High | [] |
| HIAT-HIRT:HIRT | 1 | No concerns | Low risk | No concerns | No concerns | No concerns | No concerns | High | [] |
| HIAT-HIRT:UC | 0 | No concerns | Low risk | No concerns | No concerns | No concerns | No concerns | High | [] |
| AC:HIAT | 0 | Some concerns | Low risk | No concerns | Some concerns | No concerns | No concerns | Low | ["Within-study bias","Imprecision"] |
| AC:HIRT | 0 | Some concerns | Low risk | No concerns | Some concerns | No concerns | No concerns | Low | ["Within-study bias","Imprecision"] |
| AC:UC | 0 | Some concerns | Low risk | No concerns | Some concerns | No concerns | No concerns | Low | ["Within-study bias","Imprecision"] |
| HIAT:HIAT-HIRT | 0 | No concerns | Low risk | No concerns | Some concerns | Some concerns | No concerns | Low | ["Imprecision","Heterogeneity"] |
| HIAT:HIAT-MIRT | 2 | No concerns | Low risk | No concerns | Some concerns | No concerns | No concerns | Moderate | ["Imprecision"] |
| HIAT:HIRT | 1 | No concerns | Low risk | No concerns | No concerns | Some concerns | No concerns | Moderate | ["Heterogeneity"] |
| HIAT:MIRT | 2 | No concerns | Low risk | No concerns | Some concerns | No concerns | No concerns | Moderate | ["Imprecision"] |
| HIAT-MIRT:MIRT | 2 | No concerns | Low risk | No concerns | Some concerns | No concerns | No concerns | Moderate | ["Imprecision"] |
| HIAT-MIRT:UC | 3 | No concerns | Low risk | No concerns | Some concerns | No concerns | No concerns | Moderate | ["Imprecision"] |
| MIRT:UC | 2 | No concerns | Low risk | No concerns | Some concerns | No concerns | No concerns | Moderate | ["Imprecision"] |
| AC:HIAT-HIRT | 0 | No concerns | Low risk | No concerns | No concerns | Some concerns | No concerns | Moderate | ["Heterogeneity"] |
| HIAT-HIRT:HIAT-MIRT | 0 | No concerns | Low risk | No concerns | No concerns | Some concerns | No concerns | Moderate | ["Heterogeneity"] |
| HIAT-HIRT:MIRT | 0 | No concerns | Low risk | No concerns | No concerns | Some concerns | No concerns | Moderate | ["Heterogeneity"] |
| HIAT-MIRT:HIRT | 0 | No concerns | Low risk | No concerns | Some concerns | No concerns | No concerns | Moderate | ["Imprecision"] |
| HIRT:MIRT | 0 | No concerns | Low risk | No concerns | Some concerns | No concerns | No concerns | Moderate | ["Imprecision"] |
| AC:MIRT | 1 | Some concerns | Low risk | No concerns | Some concerns | Some concerns | No concerns | Very low | ["Within-study bias","Imprecision","Heterogeneity"] |
| HIRT:UC | 2 | Some concerns | Low risk | No concerns | Some concerns | Some concerns | No concerns | Very low | ["Within-study bias","Imprecision","Heterogeneity"] |
| AC:HIAT-MIRT | 0 | Some concerns | Low risk | No concerns | Some concerns | Some concerns | No concerns | Very low | ["Within-study bias","Imprecision","Heterogeneity"] |

Supplementary Table42.GRADE certainty of evidence for the SBP outcome in middle-aged and older adults with type 2 diabetes.

| **Comparison** | **Number of studies** | **Within-study bias** | **Reporting bias** | **Indirectness** | **Imprecision** | **Heterogeneity** | **Incoherence** | **Confidence rating** | **Reason(s) for downgrading** |
| --- | --- | --- | --- | --- | --- | --- | --- | --- | --- |
| AC:HIAT | 2 | No concerns | Low risk | No concerns | No concerns | No concerns | Some concerns | Moderate | ["Incoherence"] |
| AC:MIRT | 1 | Some concerns | Low risk | No concerns | No concerns | Some concerns | Some concerns | Very low | ["Within-study bias","Heterogeneity","Incoherence"] |
| HIAT:HIAT-MIRT | 1 | Some concerns | Low risk | No concerns | Some concerns | Some concerns | No concerns | Very low | ["Within-study bias","Imprecision","Heterogeneity"] |
| HIAT:HIRT | 1 | No concerns | Low risk | No concerns | Some concerns | Some concerns | No concerns | Low | ["Imprecision","Heterogeneity"] |
| HIAT:MIRT | 1 | Some concerns | Low risk | No concerns | No concerns | Some concerns | No concerns | Low | ["Within-study bias","Heterogeneity"] |
| HIAT:UC | 2 | Some concerns | Low risk | No concerns | Some concerns | Some concerns | No concerns | Very low | ["Within-study bias","Imprecision","Heterogeneity"] |
| HIAT-MIRT:MIRT | 1 | Some concerns | Low risk | No concerns | Some concerns | No concerns | No concerns | Low | ["Within-study bias","Imprecision"] |
| HIAT-MIRT:UC | 1 | Some concerns | Low risk | No concerns | Some concerns | No concerns | No concerns | Low | ["Within-study bias","Imprecision"] |
| HIRT:UC | 5 | No concerns | Low risk | No concerns | Some concerns | Some concerns | No concerns | Low | ["Imprecision","Heterogeneity"] |
| LIRT:UC | 1 | No concerns | Low risk | No concerns | Some concerns | No concerns | Some concerns | Low | ["Imprecision","Incoherence"] |
| MIRT:UC | 1 | Some concerns | Low risk | No concerns | Some concerns | No concerns | No concerns | Low | ["Within-study bias","Imprecision"] |
| AC:HIAT-MIRT | 0 | Some concerns | Low risk | No concerns | No concerns | Some concerns | Some concerns | Very low | ["Within-study bias","Heterogeneity","Incoherence"] |
| AC:HIRT | 0 | No concerns | Low risk | No concerns | No concerns | No concerns | Some concerns | Moderate | ["Incoherence"] |
| AC:LIRT | 0 | No concerns | Low risk | No concerns | No concerns | Some concerns | Some concerns | Low | ["Heterogeneity","Incoherence"] |
| AC:UC | 0 | Some concerns | Low risk | No concerns | No concerns | Some concerns | Some concerns | Very low | ["Within-study bias","Heterogeneity","Incoherence"] |
| HIAT:LIRT | 0 | No concerns | Low risk | No concerns | Some concerns | No concerns | Some concerns | Low | ["Imprecision","Incoherence"] |
| HIAT-MIRT:HIRT | 0 | Some concerns | Low risk | No concerns | Some concerns | No concerns | No concerns | Low | ["Within-study bias","Imprecision"] |
| HIAT-MIRT:LIRT | 0 | Some concerns | Low risk | No concerns | No concerns | No concerns | No concerns | Moderate | ["Within-study bias"] |
| HIRT:LIRT | 0 | No concerns | Low risk | No concerns | Some concerns | No concerns | Some concerns | Low | ["Imprecision","Incoherence"] |
| HIRT:MIRT | 0 | Some concerns | Low risk | No concerns | Some concerns | Some concerns | No concerns | Very low | ["Within-study bias","Imprecision","Heterogeneity"] |
| LIRT:MIRT | 0 | No concerns | Low risk | No concerns | Some concerns | No concerns | Some concerns | Low | ["Imprecision","Incoherence"] |

Supplementary Table43.GRADE certainty of evidence for the HR outcome in middle-aged and older adults with type 2 diabetes.

| **Comparison** | **Number of studies** | **Within-study bias** | **Reporting bias** | **Indirectness** | **Imprecision** | **Heterogeneity** | **Incoherence** | **Confidence rating** | **Reason(s) for downgrading** |
| --- | --- | --- | --- | --- | --- | --- | --- | --- | --- |
| AC:HIAT | 2 | No concerns | Low risk | No concerns | Some concerns | No concerns | No concerns | Moderate | ["Imprecision"] |
| HIAT:HIRT | 1 | No concerns | Low risk | No concerns | Some concerns | No concerns | No concerns | Moderate | ["Imprecision"] |
| HIAT:UC | 1 | No concerns | Low risk | No concerns | Some concerns | No concerns | No concerns | Moderate | ["Imprecision"] |
| HIRT:UC | 4 | No concerns | Low risk | No concerns | Some concerns | No concerns | No concerns | Moderate | ["Imprecision"] |
| LIRT:UC | 1 | No concerns | Low risk | No concerns | Some concerns | No concerns | No concerns | Moderate | ["Imprecision"] |
| AC:HIRT | 0 | No concerns | Low risk | No concerns | Some concerns | No concerns | No concerns | Moderate | ["Imprecision"] |
| AC:LIRT | 0 | No concerns | Low risk | No concerns | Some concerns | No concerns | No concerns | Moderate | ["Imprecision"] |
| AC:UC | 0 | No concerns | Low risk | No concerns | Some concerns | No concerns | No concerns | Moderate | ["Imprecision"] |
| HIAT:LIRT | 0 | No concerns | Low risk | No concerns | Some concerns | No concerns | No concerns | Moderate | ["Imprecision"] |
| HIRT:LIRT | 0 | No concerns | Low risk | No concerns | Some concerns | No concerns | No concerns | Moderate | ["Imprecision"] |


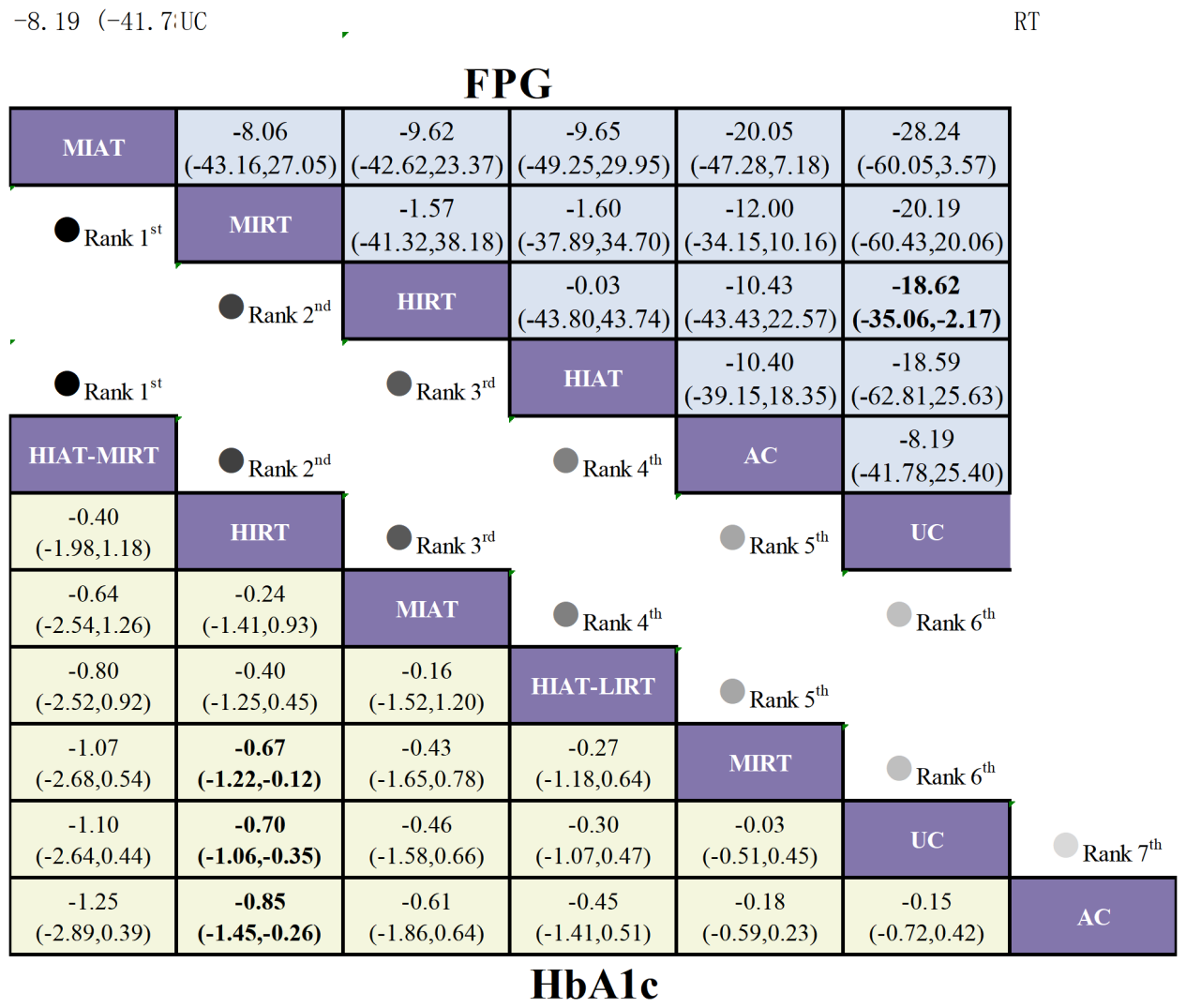
Supplementary Figure6.Subgroup analyses of HbA1c and FPG outcomes in middle-aged and older adults with type 2 diabetes

Supplementary Table44.Meta-regression analysis of baseline HbA1c for HbA1c in middle-aged and older adults with type 2 diabetes.

| **Intervention** | **Covariate** | **Coefficient** | **Standard Error** | **Z-statistic** | **P>z** | **lower confidence interval** | **upper confidence interval** |
| --- | --- | --- | --- | --- | --- | --- | --- |
| ACvsHIAT | HbA1c | 0.078726 | 0.7914574 | 0.1 | 0.921 | -1.472502 | 1.629954 |
|  | _cons | -1.299081 | 5.892717 | -0.22 | 0.826 | -12.84859 | 10.25043 |
| ACvsHIAT-LIRT | _cons | -1.17979 | 0.6891087 | -1.71 | 0.087 | -2.530418 | 0.1708386 |
| ACvsHIAT-MIRT | HbA1c | 0.182688 | 0.70517 | 0.26 | 0.796 | -1.19942 | 1.564796 |
|  | _cons | -2.220997 | 5.237804 | -0.42 | 0.672 | -12.4869 | 8.04491 |
| ACvsHIRT | HbA1c | -0.2610464 | 0.6035688 | -0.43 | 0.665 | -1.44402 | 0.9219267 |
|  | _cons | 1.3049 | 4.560765 | 0.29 | 0.775 | -7.634035 | 10.24383 |
| ACvsLIRT | HbA1c | -0.6215533 | 0.6829481 | -0.91 | 0.363 | -1.960107 | 0.7170003 |
|  | _cons | 3.8765 | 5.1639 | 0.75 | 0.453 | -6.244557 | 13.99756 |
| ACvsMIAT | HbA1c | 0.3543003 | 2.273326 | 0.16 | 0.876 | -4.101337 | 4.809938 |
|  | _cons | -3.544053 | 16.91258 | -0.21 | 0.834 | -36.6921 | 29.604 |
| ACvsMIRT | HbA1c | -0.5800143 | 0.4728478 | -1.23 | 0.22 | -1.506779 | 0.3467504 |
|  | _cons | 3.951511 | 3.464768 | 1.14 | 0.254 | -2.83931 | 10.74233 |
| ACvsUC | HbA1c | 0.4381211 | 0.5875903 | 0.75 | 0.456 | -0.7135347 | 1.589777 |
|  | _cons | -3.683765 | 4.418533 | -0.83 | 0.404 | -12.34393 | 4.9764 |

Supplementary Table45.Meta-regression analysis of baseline HbA1c for FPG in middle-aged and older adults with type 2 diabetes.

| **Intervention** | **Covariate** | **Coefficient** | **Standard Error** | **Z-statistic** | **P>z** | **lower confidence interval** | **upper confidence interval** |
| --- | --- | --- | --- | --- | --- | --- | --- |
| ACvsHIAT | HbA1c | -16.23688 | 19.24452 | -0.84 | 0.399 | -53.95544 | 21.48168 |
|  | _cons | 93.85425 | 131.1693 | 0.72 | 0.474 | -163.2328 | 350.9413 |
| ACvsHIAT-MIRT | HbA1c | 22.24298 | 50.6271 | 0.44 | 0.66 | -76.9843 | 121.4703 |
|  | _cons | -210.1179 | 389.994 | -0.54 | 0.59 | -974.4921 | 554.2563 |
| ACvsHIRT | HbA1c | 25.49839 | 50.56998 | 0.5 | 0.614 | -73.61695 | 124.6137 |
|  | _cons | -218.0414 | 391.0557 | -0.56 | 0.577 | -984.4966 | 548.4137 |
| ACvsLIRT | HbA1c | 0.5145985 | 53.36205 | 0.01 | 0.992 | -104.0731 | 105.1023 |
|  | _cons | -7.440713 | 414.7589 | -0.02 | 0.986 | -820.3532 | 805.4718 |
| ACvsMIAT | HbA1c | -133.4617 | 139.1089 | -0.96 | 0.337 | -406.1101 | 139.1867 |
|  | _cons | 967.0031 | 1045.883 | 0.92 | 0.355 | -1082.89 | 3016.897 |
| ACvsMIRT | HbA1c | -57.54537 | 32.66229 | -1.76 | 0.078 | -121.5623 | 6.471539 |
|  | _cons | 406.0054 | 238.6741 | 1.7 | 0.089 | -61.78721 | 873.798 |
| ACvsUC | HbA1c | 34.71467 | 50.0473 | 0.69 | 0.488 | -63.37623 | 132.8056 |
|  | _cons | -279.5473 | 386.6031 | -0.72 | 0.47 | -1037.275 | 478.1808 |

Supplementary Table46.Meta-regression analysis of baseline HbA1c for VO2peak in middle-aged and older adults with type 2 diabetes.

| **Intervention** | **Covariate** | **Coefficient** | **Standard Error** | **Z-statistic** | **P>z** | **lower confidence interval** | **upper confidence interval** |
| --- | --- | --- | --- | --- | --- | --- | --- |
| ACvsHIAT | HbA1c | 2.88611 | 653.4693 | 0 | 0.996 | -1277.89 | 1283.662 |
|  | _cons | -19.46533 | 4868.34 | 0 | 0.997 | -9561.237 | 9522.306 |
| ACvsHIAT-HIRT | _cons | 2.168695 | 620.7744 | 0 | 0.997 | -1214.527 | 1218.864 |
| ACvsHIAT-MIRT | HbA1c | -0.4933126 | 653.4604 | 0 | 0.999 | -1281.252 | 1280.266 |
|  | _cons | 4.69843 | 4868.272 | 0 | 0.999 | -9536.939 | 9546.336 |
| ACvsHIRT | HbA1c | 1.278062 | 653.4583 | 0 | 0.998 | -1279.477 | 1282.033 |
|  | _cons | -9.438708 | 4868.25 | 0 | 0.998 | -9551.034 | 9532.156 |
| ACvsMIRT | HbA1c | 2.953639 | 653.4785 | 0 | 0.996 | -1277.841 | 1283.748 |
|  | _cons | -21.54462 | 4868.409 | 0 | 0.996 | -9563.45 | 9520.361 |
| ACvsUC | HbA1c | 0.2179879 | 653.4621 | 0 | 1 | -1280.544 | 1280.98 |
|  | _cons | -1.753167 | 4868.282 | 0 | 1 | -9543.41 | 9539.904 |

Supplementary Table47.Meta-regression analysis of baseline HbA1c for SBP in middle-aged and older adults with type 2 diabetes.

| **Intervention** | **Covariate** | **Coefficient** | **Standard Error** | **Z-statistic** | **P>z** | **lower confidence interval** | **upper confidence interval** |
| --- | --- | --- | --- | --- | --- | --- | --- |
| ACvsHIAT | HbA1c | -6.665931 | 10.62801 | -0.63 | 0.531 | -27.49645 | 14.16458 |
|  | _cons | 34.66179 | 70.42828 | 0.49 | 0.623 | -103.3751 | 172.6987 |
| ACvsHIAT-MIRT | _cons | -13.69915 | 12.49946 | -1.1 | 0.273 | -38.19764 | 10.79933 |
| ACvsHIRT | HbA1c | 7.535033 | 13.87446 | 0.54 | 0.587 | -19.6584 | 34.72847 |
|  | _cons | -79.16978 | 101.9285 | -0.78 | 0.437 | -278.9459 | 120.6064 |
| ACvsLIRT | _cons | -15.24738 | 20.57023 | -0.74 | 0.459 | -55.56428 | 25.06953 |
| ACvsMIRT | HbA1c | -62.21752 | 75.18657 | -0.83 | 0.408 | -209.5805 | 85.14544 |
|  | _cons | 458.5205 | 561.3772 | 0.82 | 0.414 | -641.7586 | 1558.8 |
| ACvsUC | HbA1c | 8.189578 | 13.84947 | 0.59 | 0.554 | -18.95488 | 35.33403 |
|  | _cons | -80.28552 | 101.4902 | -0.79 | 0.429 | -279.2026 | 118.6316 |

Supplementary Table48.Meta-regression analysis of baseline HbA1c for HR in middle-aged and older adults with type 2 diabetes.

| **Intervention** | **Covariate** | **Coefficient** | **Standard Error** | **Z-statistic** | **P>z** | **lower confidence interval** | **upper confidence interval** |
| --- | --- | --- | --- | --- | --- | --- | --- |
| ACvsHIAT | HbA1c | 31.18359 | 24.0886 | 1.29 | 0.195 | -16.0292 | 78.39637 |
|  | _cons | -207.9417 | 159.5961 | -1.3 | 0.193 | -520.7443 | 104.8609 |
| ACvsHIAT | HbA1c | -16.59532 | 373.5188 | -0.04 | 0.965 | -748.6787 | 715.4881 |
|  | _cons | 207.3264 | 3204.996 | 0.06 | 0.948 | -6074.349 | 6489.002 |
| ACvsLIRT | _cons | 66.44436 | 73.34195 | 0.91 | 0.365 | -77.30321 | 210.1919 |
| ACvsUC | HbA1c | -14.99934 | 373.5108 | -0.04 | 0.968 | -747.0672 | 717.0685 |
|  | _cons | 193.8889 | 3204.925 | 0.06 | 0.952 | -6087.649 | 6475.426 |

Supplementary Table49.Meta-regression analysis of session duration for HbA1c in middle-aged and older adults with type 2 diabetes

| **Intervention** | **Covariate** | **Coefficient** | **Standard Error** | **Z-statistic** | **P>z** | **lower confidence interval** | **upper confidence interval** |
| --- | --- | --- | --- | --- | --- | --- | --- |
| ACvsHIAT | duration | -0.0108385 | 0.0504184 | -0.21 | 0.83 | -0.1096568 | 0.0879798 |
|  | _cons | -0.0057064 | 2.686723 | 0 | 0.998 | -5.271588 | 5.260175 |
| ACvsHIAT-LIRT | _cons | -0.5778122 | 0.3724137 | -1.55 | 0.121 | -1.30773 | 0.1521052 |
| ACvsHIAT-MIRT | duration | -0.0355386 | 0.0387111 | -0.92 | 0.359 | -0.1114109 | 0.0403336 |
|  | _cons | 1.224344 | 2.115559 | 0.58 | 0.563 | -2.922075 | 5.370763 |
| ACvsHIRT | duration | 0.0202802 | 0.01624 | 1.25 | 0.212 | -0.0115495 | 0.05211 |
|  | _cons | -1.712256 | 0.7964746 | -2.15 | 0.052 | -3.273318 | -0.1511946 |
| ACvsLIRT | duration | 0.0269161 | 0.0204116 | 1.32 | 0.187 | -0.01309 | 0.0669221 |
|  | _cons | -2.157767 | 1.034219 | -2.09 | 0.057 | -4.184798 | -0.1307353 |
| ACvsMIAT | duration | -0.0124774 | 0.0546807 | -0.23 | 0.82 | -0.1196495 | 0.0946948 |
|  | _cons | -0.0114352 | 3.137941 | 0 | 0.997 | -6.161686 | 6.138816 |
| ACvsMIRT | duration | -0.0190908 | 0.0124235 | -1.54 | 0.124 | -0.0434403 | 0.0052588 |
|  | _cons | 0.6395297 | 0.6079557 | 1.05 | 0.293 | -0.5520417 | 1.831101 |
| ACvsUC | duration | -0.0102509 | 0.0147655 | -0.69 | 0.488 | -0.0391908 | 0.018689 |
|  | _cons | 0.3372418 | 0.7041759 | 0.48 | 0.632 | -1.042918 | 1.717401 |

Supplementary Table50.Meta-regression analysis of session duration for FPG in middle-aged and older adults with type 2 diabetes.

| **Intervention** | **Covariate** | **Coefficient** | **Standard Error** | **Z-statistic** | **P>z** | **lower confidence interval** | **upper confidence interval** |
| --- | --- | --- | --- | --- | --- | --- | --- |
| ACvsHIAT | _cons | -14.13883 | 9.394205 | -1.51 | 0.132 | -32.55113 | 4.273474 |
| ACvsHIAT-MIRT | duration | 3.250671 | 8.563046 | 0.38 | 0.704 | -13.53259 | 20.03393 |
|  | _cons | -207.1099 | 513.0403 | -0.4 | 0.686 | -1212.65 | 798.4305 |
| ACvsHIRT | duration | 5.317047 | 5.851605 | 0.91 | 0.364 | -6.151889 | 16.78598 |
|  | _cons | -306.7538 | 337.4254 | -0.91 | 0.363 | -968.0954 | 354.5878 |
| ACvsLIRT | duration | 5.750932 | 5.711472 | 1.01 | 0.314 | -5.443347 | 16.94521 |
|  | _cons | -316.1272 | 333.5263 | -0.95 | 0.343 | -969.8267 | 337.5723 |
| ACvsMIAT | duration | 7.469996 | 6.167082 | 1.21 | 0.226 | -4.617262 | 19.55725 |
|  | _cons | -440.2711 | 359.4503 | -1.22 | 0.221 | -1144.781 | 264.2386 |
| ACvsMIRT | duration | -2.250731 | 1.317379 | -1.71 | 0.088 | -4.832746 | 0.331285 |
|  | _cons | 106.4329 | 71.92177 | 1.48 | 0.139 | -34.5312 | 247.3969 |
| ACvsUC | duration | 4.611007 | 5.667933 | 0.81 | 0.416 | -6.497937 | 15.71995 |
|  | _cons | -265.7317 | 332.1152 | -0.8 | 0.424 | -916.6656 | 385.2023 |

Supplementary Table51.Meta-regression analysis of session duration for VO2peak in middle-aged and older adults with type 2 diabetes

| **Intervention** | **Covariate** | **Coefficient** | **Standard Error** | **Z-statistic** | **P>z** | **lower confidence interval** | **upper confidence interval** |
| --- | --- | --- | --- | --- | --- | --- | --- |
| ACvsHIAT | duration | 0.23898 | 44.44101 | 0.01 | 0.996 | -86.86379 | 87.34175 |
|  | _cons | -11.41854 | 2666.449 | 0 | 0.997 | -5237.562 | 5214.725 |
| ACvsHIAT-HIRT | _cons | 2.282914 | 444.3983 | 0.01 | 0.996 | -868.7218 | 873.2876 |
| ACvsHIAT-MIRT | duration | -0.0432966 | 44.44088 | 0 | 0.999 | -87.14581 | 87.05922 |
|  | _cons | 2.897549 | 2666.443 | 0 | 0.999 | -5223.235 | 5229.03 |
| ACvsHIRT | duration | 0.1023031 | 44.44072 | 0 | 0.998 | -86.99991 | 87.20452 |
|  | _cons | -6.132241 | 2666.428 | 0 | 0.998 | -5232.235 | 5219.971 |
| ACvsMIRT | duration | 0.1399534 | 44.44051 | 0 | 0.997 | -86.96184 | 87.24175 |
|  | _cons | -7.937206 | 2666.426 | 0 | 0.998 | -5234.036 | 5218.161 |
| ACvsUC | duration | 0.0185925 | 44.44057 | 0 | 1 | -87.08333 | 87.12051 |
|  | _cons | -2.365262 | 2666.424 | 0 | 0.999 | -5228.46 | 5223.73 |

Supplementary Table52.Meta-regression analysis of session duration for SBP in middle-aged and older adults with type 2 diabetes.

| **Intervention** | **Covariate** | **Coefficient** | **Standard Error** | **Z-statistic** | **P>z** | **lower confidence interval** | **upper confidence interval** |
| --- | --- | --- | --- | --- | --- | --- | --- |
| ACvsHIAT | _cons | -9.505838 | 0.7930946 | -11.99 | 0.329 | -11.06028 | -7.951401 |
| ACvsHIAT-MIRT | _cons | -2.685475 | 5.739224 | -0.47 | 0.64 | -13.93415 | 8.563197 |
| ACvsHIRT | duration | -0.2152136 | 79.16714 | 0 | 0.998 | -155.38 | 154.9495 |
|  | _cons | 6.013775 | 4750.023 | 0 | 0.999 | -9303.86 | 9315.888 |
| ACvsLIRT | _cons | -2.106287 | 2375.016 | 0 | 0.999 | -4657.051 | 4652.839 |
| ACvsMIRT | _cons | -5.063678 | 3.499406 | -1.45 | 0.148 | -11.92239 | 1.795032 |
| ACvsUC | duration | -0.1273233 | 79.16709 | 0 | 0.999 | -155.292 | 155.0373 |
|  | _cons | 5.713412 | 4750.021 | 0 | 0.999 | -9304.156 | 9315.583 |

Supplementary Table53.Meta-regression analysis of session duration for HR in middle-aged and older adults with type 2 diabetes.

| **Intervention** | **Covariate** | **Coefficient** | **Standard Error** | **Z-statistic** | **P>z** | **lower confidence interval** | **upper confidence interval** |
| --- | --- | --- | --- | --- | --- | --- | --- |
| ACvsHIAT | _cons | -1.351183 | 2.299224 | -0.59 | 0.557 | -5.857579 | 3.155213 |
| ACvsHIAT | duration | 0.1582253 | 31.64388 | 0.01 | 0.996 | -61.86264 | 62.17909 |
|  | _cons | -5.667956 | 1898.609 | 0 | 0.998 | -3726.874 | 3715.538 |
| ACvsLIRT | _cons | -1.268837 | 949.3113 | 0 | 0.999 | -1861.885 | 1859.347 |
| ACvsUC | duration | 0.1508475 | 31.64387 | 0 | 0.996 | -61.87 | 62.17169 |
|  | _cons | -4.794263 | 1898.609 | 0 | 0.998 | -3725.999 | 3716.411 |

Supplementary Table54.Meta-regression analysis of frequency for HbA1c in middle-aged and older adults with type 2 diabetes.

| **Intervention** | **Covariate** | **Coefficient** | **Standard Error** | **Z-statistic** | **P>z** | **lower confidence interval** | **upper confidence interval** |
| --- | --- | --- | --- | --- | --- | --- | --- |
| ACvsHIAT | _cons | -0.4582921 | 0.3132536 | -1.46 | 0.143 | -1.072258 | 0.1556737 |
| ACvsHIAT-LIRT | _cons | -0.3565527 | 0.3679542 | -0.97 | 0.333 | -1.07773 | 0.3646243 |
| ACvsHIAT-MIRT | _cons | -0.6038851 | 0.2896925 | -2.08 | 0.057 | -1.171672 | -0.0360983 |
| ACvsHIRT | freguency | -0.4183921 | 41.68961 | -0.01 | 0.992 | -82.12853 | 81.29175 |
|  | _cons | 0.5911332 | 125.0693 | 0 | 0.996 | -244.5401 | 245.7224 |
| ACvsLIRT | freguency | 0.3009998 | 41.68988 | 0.01 | 0.994 | -81.40966 | 82.01166 |
|  | _cons | -1.766505 | 125.0714 | -0.01 | 0.989 | -246.9019 | 243.3689 |
| ACvsMIAT | freguency | 0.0282075 | 41.69032 | 0 | 0.999 | -81.68332 | 81.73974 |
|  | _cons | -0.601175 | 125.0753 | 0 | 0.996 | -245.7443 | 244.5419 |
| ACvsMIRT | freguency | 0.1471693 | 41.68946 | 0 | 0.997 | -81.56267 | 81.85701 |
|  | _cons | -0.6728353 | 125.0686 | -0.01 | 0.996 | -245.8028 | 244.4571 |
| ACvsUC | freguency | 0.0464844 | 41.68942 | 0 | 0.999 | -81.66328 | 81.75625 |
|  | _cons | -0.1960061 | 125.0687 | 0 | 0.999 | -245.3261 | 244.9341 |

Supplementary Table55.Meta-regression analysis of frequency for FPG in middle-aged and older adults with type 2 diabetes

| **Intervention** | **Covariate** | **Coefficient** | **Standard Error** | **Z-statistic** | **P>z** | **lower confidence interval** | **upper confidence interval** |
| --- | --- | --- | --- | --- | --- | --- | --- |
| ACvsHIAT | freguency | -5.434692 | 8.589684 | -0.63 | 0.527 | -22.27016 | 11.40078 |
|  | _cons | 19.56645 | 49.69658 | 0.39 | 0.694 | -77.83707 | 116.97 |
| ACvsHIAT-MIRT | freguency | -3.616946 | 10.07965 | -0.36 | 0.72 | -23.37269 | 16.1388 |
|  | _cons | 17.87619 | 50.37962 | 0.35 | 0.723 | -80.86605 | 116.6184 |
| ACvsHIRT | freguency | -8.959335 | 10.73349 | -0.83 | 0.404 | -29.99659 | 12.07792 |
|  | _cons | 35.03283 | 59.15095 | 0.59 | 0.554 | -80.90089 | 150.9666 |
| ACvsLIRT | freguency | -55.28858 | 19.57454 | -2.82 | 0.055 | -93.65396 | -16.92319 |
|  | _cons | 302.3245 | 110.2344 | 2.74 | 0.076 | 86.26915 | 518.3799 |
| ACvsMIAT | freguency | -49.67875 | 22.96009 | -2.16 | 0.06 | -94.67969 | -4.677799 |
|  | _cons | 253.2754 | 122.5489 | 2.07 | 0.059 | 13.08399 | 493.4668 |
| ACvsMIRT | freguency | -2.175998 | 9.505157 | -0.23 | 0.819 | -20.80576 | 16.45377 |
|  | _cons | -5.509499 | 32.53018 | -0.17 | 0.866 | -69.26748 | 58.24848 |
| ACvsUC | freguency | -21.0888 | 9.018654 | -2.34 | 0.089 | -38.76503 | -3.412558 |
|  | _cons | 113.3256 | 48.02853 | 2.36 | 0.068 | 19.19146 | 207.4598 |

Supplementary Table56.Meta-regression analysis of frequency for VO2peak in middle-aged and older adults with type 2 diabetes

| **Intervention** | **Covariate** | **Coefficient** | **Standard Error** | **Z-statistic** | **P>z** | **lower confidence interval** | **upper confidence interval** |
| --- | --- | --- | --- | --- | --- | --- | --- |
| ACvsHIAT | _cons | 2.508009 | 1.7057 | 1.47 | 0.141 | -0.8351018 | 5.851119 |
| ACvsHIAT-HIRT | _cons | 3.116343 | 2.158882 | 1.44 | 0.149 | -1.114988 | 7.347675 |
| ACvsHIAT-MIRT | _cons | 1.1644 | 1.69347 | 0.69 | 0.492 | -2.15474 | 4.48354 |
| ACvsHIRT | _cons | -0.1836551 | 1.969898 | -0.09 | 0.926 | -4.044584 | 3.677273 |
| ACvsMIRT | _cons | 0.4599847 | 1.382307 | 0.33 | 0.739 | -2.249287 | 3.169257 |
| ACvsUC | _cons | -0.6315561 | 1.704657 | -0.37 | 0.711 | -3.972622 | 2.70951 |

Supplementary Table57.Meta-regression analysis of frequency for SBP in middle-aged and older adults with type 2 diabetes

| **Intervention** | **Covariate** | **Coefficient** | **Standard Error** | **Z-statistic** | **P>z** | **lower confidence interval** | **upper confidence interval** |
| --- | --- | --- | --- | --- | --- | --- | --- |
| ACvsHIAT | _cons | -9.503907 | 0.793087 | -11.98 | 0.146 | -11.05833 | -7.949485 |
| ACvsHIAT-MIRT | _cons | -2.832074 | 5.73315 | -0.49 | 0.621 | -14.06884 | 8.404694 |
| ACvsHIRT | freguency | 2.569232 | 3328.139 | 0 | 0.999 | -6520.463 | 6525.602 |
|  | _cons | -13.95462 | 9984.421 | 0 | 0.999 | -19583.06 | 19555.15 |
| ACvsLIRT | _cons | -6.314022 | 7.122849 | -0.89 | 0.375 | -20.27455 | 7.646507 |
| ACvsMIRT | _cons | -5.114009 | 3.498232 | -1.46 | 0.144 | -11.97042 | 1.7424 |
| ACvsUC | freguency | 1.657782 | 3328.138 | 0 | 1 | -6521.373 | 6524.689 |
|  | _cons | -7.287369 | 9984.418 | 0 | 0.999 | -19576.39 | 19561.81 |

Supplementary Table58.Meta-regression analysis of frequency for HR in middle-aged and older adults with type 2 diabetes

| **Intervention** | **Covariate** | **Coefficient** | **Standard Error** | **Z-statistic** | **P>z** | **lower confidence interval** | **upper confidence interval** |
| --- | --- | --- | --- | --- | --- | --- | --- |
| ACvsHIAT | _cons | -1.351345 | 2.173132 | -0.62 | 0.534 | -5.610605 | 2.907916 |
| ACvsHIAT | freguency | -2.628086 | 926.6419 | 0 | 0.998 | -1818.813 | 1813.557 |
|  | _cons | 12.02374 | 2779.942 | 0 | 0.997 | -5436.563 | 5460.61 |
| ACvsLIRT | _cons | 3.027885 | 6.782253 | 0.45 | 0.655 | -10.26509 | 16.32086 |
| ACvsUC | freguency | -1.572289 | 926.6418 | 0 | 0.999 | -1817.757 | 1814.612 |
|  | _cons | 8.744757 | 2779.941 | 0 | 0.997 | -5439.84 | 5457.33 |

Supplementary Table59.Sensitivity analysis among middle-aged and older adults with type 2 diabetes mellitus not using R values.

|  | **Octcomes** | **R=0.5** | **R=0.75** | **R=0.25** |
| --- | --- | --- | --- | --- |
| HIRT vs UC | HbA1c | 0.62 (0.30,0.93) | 0.64 (0.28,0.91) | 0.59 (0.34,0.93) |
| MIAT vs UC | HbA1c | 0.58 (0.05,1.10) | 0.55 (0.08,1.06) | 0.61 (0.06,1.13) |
| HIAT-MIRT vs UC | HbA1c | 0.54 (0.06,1.02) | 0.57 (0.09,1.06) | 0.51 (0.04,1.01) |
| LIRT vs UC | HbA1c | 0.54 (0.09,1.00) | 0.55 (0.12,1.03) | 0.52 (0.08,1.04) |
| HIAT vs UC | HbA1c | 0.36 (-0.21,0.93) | 0.39 (-0.19,0.91) | 0.35 (-0.23,0.89) |
| HIAT-LIRT vs UC | HbA1c | 0.30 (-0.43,1.03) | 0.32 (-0.46,1.07) | 0.29 (-0.36,1.01) |
| MIRT vs UC | HbA1c | 0.08 (-0.30,0.45) | 0.06 (-0.28,0.41) | 0.09 (-0.33,0.47) |
| AC vs UC | HbA1c | 0.13 (-0.36,0.62) | 0.15 (-0.32,0.67) | 0.17 (-0.29,0.58) |
| MIRT vs UC | FPG | 29.13 (0.58,57.68) | 29.09 (0.56,57.64) | 29.24 (0.48,57.56) |
| HIAT vs UC | FPG | 24.72 (-0.51,49.95) | 24.59 (-0.47,49.86) | 24.76 (-0.53,49.84) |
| HIAT-MIRT vs UC | FPG | 22.15 (-3.29,47.59) | 22.04 (-3.16,47.46) | 22.08 (-3.35,47.64) |
| MIAT vs UC | FPG | 18.79 (-6.74,44.31) | 18.61 (-6.53,44.36) | 18.84 (-6.66,44.24) |
| HIRT vs UC | FPG | 14.78 (-1.20,30.76) | 14.76 (-1.09,30.54) | 14.63 (-1.31,30.62) |
| AC vs UC | FPG | 12.08 (-14.40,38.55) | 12.20 (-14.21,38.36) | 12.15 (-14.31,38.63) |
| LIRT vs UC | FPG | 4.03 (-19.69,27.75) | 4.14 (-19.51,27.63) | 4.21 (-19.53,27.52) |
| HIAT-HIRT vs UC | VO2peak | -3.75 (-6.38,-1.11) | -3.68 (-6.24,-1.26) | -3.79 (-6.45,-1.04) |
| HIAT vs UC | VO2peak | -3.14 (-4.95,-1.33) | -3.05 (-4.76,-1.21) | -3.16 (-4.85,-1.46) |
| HIAT-MIRT vs UC | VO2peak | -1.80 (-3.48,-0.11) | -1.76 (-3.31,-0.19) | -1.74 (-3.69,-0.26) |
| MIRT vs UC | VO2peak | -1.09 (-3.05,0.86) | -1.13 (-3.24,0.63) | -1.21 (-3.16,0.59) |
| AC vs UC | VO2peak | -0.63 (-3.97,2.70) | -0.52 (-3.76,2.58) | -0.71 (-3.86,2.77) |
| HIRT vs UC | VO2peak | -0.45 (-2.43,1.54) | -0.39 (-2.26,1.49) | -0.53 (-2.61,1.47) |
| HIAT vs UC | SBP | 7.10 (-0.64,14.84) | 7.06 (-0.56,14.64) | 7.24 (-0.83,14.63) |
| HIRT vs UC | SBP | 3.68 (0.16,7.20) | 3.59 (0.23,7.15) | 3.49 (0.26,7.32) |
| LIRT vs UC | SBP | 4.00 (-7.53,15.53) | 4.13 (-7.42,15.68) | 4.24 (-7.31,15.42) |
| MIRT vs UC | SBP | 2.72 (-6.87,12.31) | 2.68 (-6.79,12.42) | 2.81 (-6.93,12.27) |
| HIAT vs UC | SBP | 0.46 (-11.34,12.26) | 0.41 (-11.13,12.34) | 0.54 (-11.41,12.14) |
| AC vs UC | SBP | 2.41 (-5.45,10.26) | 2.36 (-5.31,10.42) | 2.53 (-5.39,10.08) |
| HIAT vs UC | HR | 5.65 (-2.21,13.52) | 5.47 (-2.14,13.42) | 5.72 (-2.19,13.63) |
| AC vs UC | HR | 4.30 (-4.48,13.07) | 4.24 (-4.56,13.14) | 4.41 (-4.36,13.21) |
| LIRT vs UC | HR | 1.00 (-8.19,10.19) | 1.05 (-8.24,10.16) | 1.17 (-8.02,10.14) |
| HIRT vs UC | HR | 0.49 (-3.05,4.03) | 0.46 (-3.16,4.21) | 0.41 (-3.13,4.26) |

Supplementary Table60.Baseline classification of exercise interventions and assigned network nodes in middle-aged and older adults with type 2 diabetes

| **First Author** | **Original intervention description** | **Reported intensity indicator** | **Classification rule applied** | **Final assigned network node** |
| --- | --- | --- | --- | --- |
| [Cíntia E Botton](https://pubmed.ncbi.nlm.nih.gov/?term=Botton+CE&cauthor_id=30296453) | A 12-week resistance-training programme was performed three times per week. Conventional exercises included leg press, leg extension, leg curl, hip abduction, inclined bench press, low row, biceps curl, triceps exercise, and abdominal crunches. During weeks 1–8, exercises were prescribed at 15RM for 12 repetitions; during weeks 9–12, they progressed to 12RM for 10 repetitions. Functional exercises were progressed using the OMNI scale. | Progression from 15RM to 12RM; load or step height was increased for functional exercises when OMNI-RPE was <6. | For resistance training, 8–12RM was classified as moderate intensity. Where a progression scheme was specified, classification was based on the highest target intensity. The highest clearly prescribed intensity was 12RM, with no evidence of progression to ≤6–8RM or ≥70% 1RM. | Moderate-intensity resistance training |
| N W Cheung | The 16-week programme began with five supervised group sessions, followed by home-based elastic-band training for 30 minutes, five days per week. Each exercise was performed for 2 sets of 12 repetitions. Band resistance was increased when participants could complete 12 repetitions with proper technique. Exercises included chest press, seated row, leg abduction, leg extension, seated leg press, triceps exercise, and biceps exercise. | 2 × 12 repetitions; progressive increase in elastic-band tension; the authors estimated the 30-minute session to correspond to approximately 3 METs, comparable to moderate-intensity walking. | Neither 12RM nor %1RM was explicitly reported; 12 ordinary repetitions cannot be assumed to represent 12RM. If classification by training prescription is required for the network, the intervention may be classified as moderate-intensity resistance training, but it does not meet criteria for high intensity. | Moderate-intensity resistance training |
| Yutaro Yamamoto | A 48-week home-based programme combined elastic-band and body-weight resistance exercise for approximately 15 minutes daily. Elastic bands provided 1.3–3.3 kg of resistance, with different starting colours. Upper-limb exercises included tube fly, front raise, and hammer curl; lower-limb exercises included leg extension, calf raise, and squat. Each exercise was performed for 20 repetitions daily, and participants progressed to a higher-resistance band when 20 repetitions could be completed easily. | 20 repetitions per exercise; absolute band resistance of 1.3–3.3 kg; no %1RM, RM, or RPE was reported. | Neither 12RM nor %1RM was explicitly reported; 20 ordinary repetitions cannot be equated with RM-based intensity. If classification by training prescription is required for the network, the intervention may be treated as moderate-intensity resistance training, but it does not meet criteria for high intensity. | Moderate-intensity resistance training |
| Yu-Hsuan Chien | A 12-week home-based sandbag resistance-training programme included arm curl, overhead press, hip adduction/abduction, step exercise, and tiptoe exercise. The initial sandbag load was 0.5 kg. Each exercise was performed for 8–15 repetitions at RPE 13, three times per week; when participants could easily complete 20 repetitions, the load was increased to 1 kg. | RM 9; 8–15 repetitions; progression to 20 repetitions. | For resistance training, 8–12RM was classified as moderate intensity. Where progression was specified, classification was based on the highest target intensity. The highest clearly reported intensity was 9RM, with no progression to ≤6–8RM or ≥70% 1RM. | Moderate-intensity resistance training |
| Nikolaos P. E. Kadoglou | A 3-month supervised resistance-training programme was performed three times per week, with session duration progressing from 45 to 60 minutes. Machine-based resistance training involved 2–3 sets of eight major exercises, including seated leg press, knee extension/flexion, chest press, lat pulldown, overhead press, and biceps/triceps exercises. Rest intervals were approximately 1 minute between sets or exercises and 3 minutes between exercise blocks. | 60–80% 1RM; 6–8 repetitions per exercise; 1RM was adjusted during the first 4 weeks. | For resistance training, ≥70% 1RM or ≤6–8RM was classified as high intensity. The reported range of 60–80% 1RM crossed the high-intensity threshold, and 6–8 repetitions also met the high-intensity criterion. Classification was therefore based on the highest prescribed intensity. | High-intensity resistance training |
| Ping-Lun Hsieh | A 12-week supervised resistance-training programme was performed three times per week. Exercises included chest press, shoulder press, biceps curl, hip abduction, standing hip flexion, leg press, standing calf raise, and abdominal crunch. Each exercise was performed for 3 sets of 8–12 repetitions, with 60–90 seconds of rest between sets. Training began at 40–50% 1RM or Borg RPE 12–13 and progressed to a target of 75% 1RM or Borg RPE 14–16 by week 12. Actual week-12 intensity was 74.3% 1RM for upper-limb exercises and 76.9% 1RM for lower-limb exercises. | 40–50% 1RM progressing to 75% 1RM; actual intensity approximately 74–77% 1RM; Borg RPE 14–16. | Where resistance training included a clearly defined progression plan, classification was based on the highest target intensity. The target intensity reached ≥70% 1RM; therefore, the intervention was classified as high intensity. | High-intensity resistance training |
| Anderson Rech | A 12-week progressive resistance-training programme was performed three times per week. It included partial squat, bench stepping, unilateral leg press, unilateral knee extension, knee flexion, plantar flexion, bench press, low row, biceps curl, elbow extension, hip abduction, and abdominal crunches. Conventional resistance exercises progressed from 12 to 10 repetitions for 2–3 sets. The initial load was determined from 15RM; later training used a load equivalent to 12RM for 10 repetitions to avoid failure. | 15RM load for 12 repetitions; later 12RM load for 10 repetitions; functional exercises controlled using the OMNI scale. | The highest explicit target intensity was 12RM. Under the prespecified rule, 8–12RM was classified as moderate intensity. The intervention did not reach ≤6–8RM or ≥70% 1RM. | Moderate-intensity resistance training |
| George Mavros | A 12-month supervised high-intensity, high-velocity progressive resistance-training programme was performed three times per week using Keiser pneumatic equipment. Exercises included seated row, chest press, leg press, knee extension, hip flexion, hip extension, and hip abduction. Each exercise was performed for 3 sets of 8 repetitions; unilateral hip exercises were performed for 2 sets of 8 repetitions per side. 1RM was reassessed every 4 weeks. | 80% of the most recently measured 1RM; if 1RM could not be tested, intensity was adjusted using Borg RPE 15–18. | ≥70% 1RM was classified as high intensity. Eight repetitions were also close to the high-intensity/moderate-to-high boundary. The primary intensity indicator, 80% 1RM, clearly met the high-intensity criterion. | High-intensity resistance training |
| Theng Choon Ooi | A 16-week home-based progressive resistance-training programme used elastic tubing. Twelve exercises were prescribed: chest press, shoulder press, overhead pulldown, lateral shoulder raise, biceps curl, triceps extension, hip flexion, hip extension, calf raise, leg extension, squat, and seated row. The formal training prescription was 3 sets of 8–10 repetitions, emphasizing rapid concentric and slow eccentric contractions. Resistance was progressed by switching to higher-resistance tubing. | No baseline 1RM testing was performed; Borg RPE 16–18 was used to define high intensity; each exercise was performed for 8–10 repetitions per set. | The study explicitly prescribed Borg RPE 16–18, which satisfies the criterion for high-intensity progressive resistance training. | High-intensity resistance training |
| Xiaojun Ma | A 6-month supervised group-based resistance-training programme was performed three times per week for 50 minutes per session. Equipment included barbells, dumbbells, elastic bands/ropes, and kettlebells. Exercises targeted the upper limbs, hips and legs, and core. The first 2 weeks served as familiarisation; formal training began at 55–60% 1RM and progressed to 60–70% 1RM. Each exercise was performed for 8–15 repetitions and 2–4 sets, with 60 seconds of rest between sets. | 55–60% progressing to 60–70% 1RM; RPE 12–14; 8–15 repetitions. | The programme reached 70% 1RM, and ≥70% 1RM was the threshold for high-intensity resistance training. | High-intensity resistance training |
| Carmen Castaneda | A 16-week supervised progressive resistance-training programme was performed three times per week. Each 45-minute session included a 5-minute warm-up, 35 minutes of progressive resistance training, and a 5-minute cool-down. Five pneumatic resistance machines were used: chest press, leg press, upper back, knee extension, and knee flexion. Each machine exercise was performed for 3 sets of 8 repetitions. Training intensity was 60–80% of baseline 1RM during weeks 1–8 and 70–80% of mid-study 1RM during weeks 10–14; intensity was reduced by approximately 10% during weeks 9 and 15 to reduce the risk of injury or overtraining. | 60–80% 1RM progressing to 70–80% 1RM; mean actual training intensity 70.2% 1RM, range 66–75%. | For resistance training, ≥70% 1RM was classified as high intensity. The study specified progression to 70–80% 1RM, and the mean actual intensity was 70.2% 1RM; classification was therefore based on the highest target intensity. | High-intensity resistance training |
| David W Dunstan | In this 6-month randomized controlled trial, participants trained in the laboratory three times per week. Each session included a 5-minute low-intensity stationary cycling warm-up, approximately 45 minutes of high-intensity dynamic resistance training, and a 5-minute cool-down. Nine exercises were performed: bench press, leg extension, upright row, lateral pulldown, standing leg curl, seated shoulder press, biceps curl, triceps kickback, and abdominal curls. Except for abdominal curls, each exercise was performed for 3 sets of 8–10 repetitions. Load was increased when participants could complete 3 sets of 10 repetitions with proper technique. 1RM was reassessed every 12 weeks. | Weeks 1–2: 50–60% 1RM; thereafter, target intensity 75–85% of current 1RM; 3 sets of 8–10 repetitions. | The resistance-training prescription clearly progressed from 50–60% 1RM to 75–85% 1RM. Because the highest target intensity exceeded ≥70% 1RM, the intervention was classified as high intensity. | High-intensity resistance training |
| Kenneth M Madden | A 3-month supervised aerobic-training programme was performed three times per week. Each 60-minute session included a 10-minute warm-up, 20 minutes on a treadmill, 20 minutes on a cycle ergometer, and a 10-minute cool-down/stretching period. Target heart rate was prescribed from HRmax measured during a maximal exercise test. | Weeks 1–2: 50–60% HRmax; thereafter, progression to 80–85% HRmax. The abstract also described the intervention as vigorous aerobic exercise at 80–85% HRmax. | For aerobic training, ≥77% HRmax was classified as high intensity. The intervention progressed to 80–85% HRmax; therefore, classification was based on the highest target intensity. | High-intensity aerobic training |
| Kenneth M Madden | A 3-month supervised aerobic-training programme was performed three times per week. Each 60-minute session comprised a 10-minute warm-up, 40 minutes of aerobic exercise, and a 10-minute cool-down/stretching period. Moderate-to-vigorous aerobic exercise, including treadmill and cycle-ergometer training, was supervised by a clinical exercise physiologist. | Target training heart rate was 60–75% HRR, calculated using the Karvonen formula, with continuous heart-rate monitoring. | For aerobic training, ≥60% HRR was classified as high intensity. Because the target range was 60–75% HRR, the intervention reached the high-intensity threshold and was classified according to the highest target intensity. | High-intensity aerobic training |
| Kiwol Sung | A 6-month regular walking programme was performed three times per week for 50 minutes per session, alongside dietary counselling and education on diabetes-complication management. Walking was progressed in stages: normal walking for 30 minutes during weeks 1–4, brisk walking for 35 minutes during weeks 5–14, and brisk walking for 40 minutes during weeks 15–24. Each stage also included 5–10 minutes of warm-up and cool-down. | Weeks 1–4: 55–64% HRmax or RPE 11–13; weeks 5–24: 65–75% HRmax or RPE 13–15. | For aerobic training, 64–76% HRmax or RPE 12–13 was classified as moderate intensity, whereas ≥77% HRmax or RPE ≥14 was classified as high intensity. The highest HRmax target was 75%, corresponding to moderate intensity; although RPE 13–15 partly crossed the high-intensity threshold, the primary heart-rate target did not reach ≥77% HRmax. A conservative moderate-intensity classification was therefore applied, with the RPE boundary noted. | Moderate-intensity aerobic training |
| Hwi Ryun Kwon | A 12-week elastic-band resistance-training programme was performed three days per week. Each 60-minute session included a 10-minute warm-up, 40 minutes of resistance training, and a 10-minute cool-down. Exercises included biceps curls, triceps extensions, upright rows, shoulder presses, chest presses, seated rows, leg presses, hip flexion, leg flexion, leg extensions, and side bends. | Elastic-band intensity was progressively increased, ultimately reaching 40–50% 1RM; each exercise was performed for 3 sets of 10–15 repetitions. | The moderate-intensity threshold for resistance training was 50–69% 1RM. The maximum reported intensity was 40–50% 1RM, reaching only the lower boundary of moderate intensity; the intervention was predominantly low intensity. | Low-intensity resistance training |
| Maryam Nadi | A 12-week programme, performed three times per week, included leg press, arm curls, military press, push-ups, squats, knee extensions, heel raises, back extensions, knee sit-ups, and upright rowing. Each session included 10-minute warm-up and cool-down periods. | 30% repetition maximum. | If interpreted as %1RM, 30% is below the 50–69% 1RM range used for moderate intensity and does not meet criteria for moderate or high intensity. | Low-intensity resistance training |
| R C Plotnikoff | A 16-week home-based resistance-training programme was performed three days per week using a multigym and dumbbells, with supervision by a qualified exercise specialist that was gradually tapered. Core exercises included squats, seated row, chest press, and shoulder press; supplementary exercises included lunges, lat pulldown, triceps extension, abdominal twists, biceps curl, and reverse fly. | Week 1: 50–60% 1RM, 2 sets of 10–12 repetitions; weeks 3–8: progressive loading, with a target of 70–80% 1RM by weeks 5–8; weeks 10–15: 70–85% 1RM; week 16: recovery week at 80% 1RM. | The programme included explicit progression to a highest target intensity of 70–85% 1RM. Because ≥70% 1RM was classified as high intensity, classification was based on the highest target intensity. | High-intensity resistance training |
| Lauren M Sparks | A 9-month supervised aerobic-training programme was prescribed at 12 kcal/kg/week, with 5-minute warm-up and cool-down periods in each session. Speed, grade, heart rate, and Borg RPE were recorded every 5 minutes. A 9-month supervised resistance-training programme was performed three days per week and included 2 sets of four upper-body exercises—bench press, seated row, shoulder press, and lat pulldown—and 3 sets of three lower-body exercises—leg press, leg extension, and leg flexion. Abdominal crunches and back extensions were also included. | Exercise intensity ranged from 50% to 80% VO₂peak. Resistance exercises were performed for 10–12 repetitions per set, and load was increased after participants completed 12 repetitions in two consecutive sessions. No %1RM or explicit RM was reported. | VO₂R, HRR, HRmax, and RPE are conventional aerobic-intensity metrics; VO₂peak is not fully equivalent to VO₂R. Nevertheless, the highest prescribed intensity reached 80% VO₂peak, clearly exceeding moderate intensity and entering a higher aerobic-intensity range. Under strict ACSM-based classification, intensity would be considered incompletely quantified. If network classification is required, the resistance component may be treated as a moderate-intensity progressive resistance-training format. | High-intensity aerobic training plus moderate-intensity resistance training |
| Vanessa Neves de Oliveira | A 12-week resistance-training programme was performed three times per week and included seven exercises targeting large muscle groups: leg press, bench press, lat pulldown, seated row, shoulder press, abdominal curls, and knee curls. During weeks 1–2, participants performed 2–4 sets of 10–15 repetitions at 50% 1RM; during weeks 3–12, they performed 4 sets of 8–12RM to failure. A 12-week combined aerobic and strength-training programme was also performed three times per week, using the same intensities as the aerobic-training and strength-training groups but with half the training volume of each. | Weeks 1–2: 50% 1RM; weeks 3–12: 8–12RM. Aerobic training was prescribed at LT. Resistance training used 50% 1RM during weeks 1–2 and 8–12RM during weeks 3–12; combined training used a lower volume. | Both 50% 1RM and 8–12RM met the moderate-intensity criterion. The resistance component did not reach ≥70% 1RM or ≤6–8RM. | Moderate-intensity aerobic training plus moderate-intensity resistance training |
| Alireza Mehdizadeh | A 12-week aerobic-training programme was performed three times per week using treadmill walking/running. Each session began with a 10-minute warm-up, followed by aerobic exercise that progressed from lower intensity to longer duration and higher intensity. A 12-week resistance-training programme was also performed three times per week. It included nine exercises, each performed for 3 sets of 10 repetitions with 60–90 seconds of rest between sets. Each session lasted approximately 1 hour, including 10 minutes of warm-up, 40–45 minutes of main training, and 5 minutes of cool-down. Exercises included bench press, shoulder press, standing cable curl, rope press-down, leg press, leg extension, leg flexion, abdominal exercise, and back extension. The combined-training programme included both aerobic and resistance training at the same intensity, duration, and frequency as the respective single-mode groups. During the first 2 weeks, participants completed two aerobic and one resistance session per week; during the next 2 weeks, they completed two resistance and one aerobic session per week, with this pattern alternating through week 12. | The abstract reported 20–50 min/day at 60–80% HRmax. The methods described progression from 40–50% HRmax for 20 minutes to 70–80% HRmax for 45–50 minutes. Resistance training progressed from 40–45% 1RM in weeks 1–4, to 50–55% 1RM in weeks 5–8, and to 60–65% 1RM in weeks 9–12. Thus, aerobic training reached 70–80% HRmax, whereas resistance training reached 60–65% 1RM. | For aerobic training, 64–76% HRmax was classified as moderate intensity and ≥77% HRmax as high intensity. Because the programme explicitly progressed to 70–80% HRmax, the highest target intensity reached the high-intensity threshold. For resistance training, 50–69% 1RM was classified as moderate intensity and ≥70% 1RM as high intensity. The highest resistance-training intensity was 60–65% 1RM, corresponding to moderate intensity. | Moderate-intensity resistance training plus high-intensity aerobic training |
| Giorgio Orlando | An 8-week resistance-training programme was performed three times per week for 24 sessions, with each session lasting approximately 50 minutes. Exercises included unilateral leg extension, step-up, bilateral lat pulldown, and chest press. Each exercise was performed for 3 sets of 10 repetitions plus a fourth set to failure, with 2 minutes of passive recovery between sets. Load was increased when participants could complete at least 12 repetitions in two consecutive sessions. An 8-week combined-training programme was performed three times per week for 75 minutes per session and included the same 50-minute resistance-training protocol followed by HIIT. HIIT consisted of 10 × 1-minute high-intensity cycling intervals, each separated by 1 minute of low-intensity cycling. | Resistance training: 70–80% 1RM; 3 sets of 10 repetitions plus a fourth set to failure. HIIT targeted 90% HRmax, with recovery at 30–40% HRmax; workload was increased if mean heart rate across the 10 aerobic intervals was <85% HRmax. | For resistance training, ≥70% 1RM was classified as high intensity. The prescribed resistance intensity was 70–80% 1RM, clearly meeting this threshold. For the aerobic/HIIT component, ≥77% HRmax was classified as high intensity, and the target was 90% HRmax. | High-intensity aerobic training plus high-intensity resistance training |
| Hwi Ryun Kwon | A 12-week brisk-walking programme was performed five days per week for 60 minutes per day, with daily activity and training intensity monitored using a multi-sensor accelerometer. A 12-week elastic-band resistance-training programme was performed three times per week; each 60-minute session included a 10-minute warm-up, 40 minutes of resistance training, and a 10-minute cool-down. Exercises included biceps curls, triceps extensions, upright rows, shoulder/chest press, seated rows, trunk side bends, leg press, hip flexion, leg flexion, and leg extension. | Accelerometer intensity level 4–6, corresponding to 3.6–6.0 METs; the article described the programme as moderate intensity. Resistance training used the lowest band setting during the first 2 weeks, followed by gradual progression, with the maximum intensity reaching 40–50%; each exercise was performed for 3 sets of 10–15 repetitions. | An intensity of 3.6–6.0 METs generally corresponds to moderate intensity. For resistance training, 50–69% 1RM was defined as moderate intensity. The resistance component reached only 40–50%, touching the lower boundary of moderate intensity, with most training below the moderate-intensity range. | Moderate-intensity aerobic training plus low-intensity resistance training |
| Y H Ku | A 12-week elastic-band resistance-training programme was performed five times per week, including three hospital-based group sessions and two home-based sessions. Each session included 10 exercises: biceps curl, triceps extension, upright row, shoulder/chest press, trunk side-bending, seated row, leg press, hip flexion, leg flexion, and leg extension. Each exercise was performed for 3 sets of 15–20 repetitions. A 12-week walking programme was also performed five times per week for 60 minutes per session. | Resistance intensity was 40–50% of maximal exercise capacity, with 3 sets of 15–20 repetitions. Walking intensity was 3.6–5.2 METs and described as moderate intensity. | The reported resistance intensity was maximal exercise capacity rather than 1RM. Even if approximated as %1RM, 40–50% is below, or at most reaches, the lower boundary of moderate intensity; the 15–20-repetition prescription also indicates high-repetition, low-load training. | Moderate-intensity aerobic training plus low-intensity resistance training |
| Niloufar Ghadamyari | An 8-week endurance-training programme was performed three days per week, totalling 180–190 minutes per week, and consisted of walking and jogging. Each session included a 10-minute warm-up and a 5-minute cool-down. An 8-week resistance-training programme was performed three times per week for 60 minutes per session. Nine stations were used: leg extension, leg curl, chest press, leg press, seated cable row, lat pulldown behind the neck, cable crunch, dumbbell biceps curl, and dumbbell triceps curl. Each session included warm-up and cool-down periods. | Aerobic training: week 1, 50–60% HRmaxR; week 2, 60–70% HRmaxR; final 6 weeks, 70–75% HRmaxR. Here, HRmaxR corresponds to heart-rate reserve. Resistance training: week 1, 50–60% 1RM; week 2, 60–70% 1RM; final 6 weeks, 70–75% 1RM; each exercise was performed for 3 sets of 8–9 repetitions. | For aerobic training, ≥60% HRR was classified as high intensity. For resistance training, ≥70% 1RM or ≤6–8RM was classified as high intensity. The programme explicitly progressed to 70–75% 1RM; classification was therefore based on the highest target intensity. | High-intensity aerobic training plus high-intensity resistance training |
| C Blioumpa | A 6-week home-based, real-time video-supervised exercise programme was performed three times per week for 60 minutes per session. Each session included a 10-minute warm-up, 40 minutes of combined aerobic and strength training, and a 10-minute recovery period. The telerehabilitation programme was supervised live by video conference. The 20-minute aerobic component included marching in place, modified jumping jacks, mini-squats, knee-to-elbow movements, lunges, step-ups, and boxing movements. The 20-minute strength component included mini-squats, sit-to-stand, biceps curl, wall press, knee extension, hip abduction/extension, and hamstring curl. | Aerobic training began at 60% HRR and increased weekly, targeting 60–80% HRR. Strength training was prescribed at Borg RPE 13–14, with 2 sets of 10 repetitions. The authors noted that RPE 14 may correspond in older adults to 8–12 repetitions and approximately 40% 1RM. | For aerobic training, ≥60% HRR was classified as high intensity. The programme reached 80% HRR and was therefore classified according to the highest target intensity. | High-intensity aerobic training plus low-intensity resistance training |
| Karolina S Khan | A 12-week supervised progressive resistance-training programme was conducted in a university department of exercise biology. Sessions lasted approximately 1 hour and were supervised by two coaches. Exercises included leg press, bench press, pulldowns, knee flexion/extension, ankle plantar/dorsal flexion, abdominal crunches, and back extensions. Each session began with a 10-minute moderate-intensity stationary cycling warm-up. A total of 30 sessions were planned, at a frequency of two to three sessions per week. | 70–75% 1RM; each exercise was performed for 3 sets of 8–9 repetitions. | For resistance training, ≥70% 1RM or ≤6–8RM was classified as high intensity. The programme explicitly progressed to 70–75% 1RM; classification was therefore based on the highest target intensity. | High-intensity resistance training |
| Francesca Galle | A 9-month community-based exercise programme combined exercise training with motivational interviewing. Exercise was performed two to three times per week on non-consecutive days, for 1 hour per session. Sessions included a 5-minute warm-up, 20 minutes of aerobic exercise, 20 minutes of circuit resistance training, 10 minutes of agility/balance training, and 5 minutes of flexibility training. Twelve group motivational-interviewing sessions were also provided. | Aerobic training involved moderate- to high-intensity brisk/interval walking using external loads, obstacles, and stair circuits, with Borg RPE 12–17. Resistance training involved 20 bilateral or 30 unilateral repetitions; %1RM and RM were not reported. | For aerobic training, RPE 12–13 was classified as moderate intensity and RPE ≥14 as high intensity. Because the programme reached RPE 17, it was classified according to the highest target intensity. The resistance component reported only ordinary repetition counts, not RM; therefore, it could not be classified using 8–12RM or ≤6–8RM criteria. | High-intensity aerobic training plus low-intensity resistance training |
| Naﬁseh Ghodrati | A 12-week combined-training programme was performed three times per week, with each session lasting approximately 65 minutes. Sessions included a 10-minute warm-up, 20 minutes of aerobic exercise, 30 minutes of resistance training, approximately 3 minutes of balance training, and a 5-minute cool-down. Training included forward/backward and lateral movements, elliptical exercise, and stationary cycling. Resistance exercises included leg press, chest press, latissimus exercises, forearm exercises, and hamstring exercises. Balance exercises included tandem walking, standing on tiptoes, and single-leg standing. | Aerobic training: weeks 1–6, 55% HRR; weeks 7–12, 65–75% HRR. Table 1 also reported weeks 1–6 as 55–65% HRR and weeks 7–12 as 65–75% HRR. Resistance training: weeks 1–4, 65–75% 1RM; weeks 5–8, 75–80% 1RM; weeks 9–12, 80–85% 1RM. Repetitions progressed from 10–12 to 6–8. | For aerobic training, ≥60% HRR was classified as high intensity; the later phase reached 65–75% HRR. For resistance training, ≥70% 1RM or ≤6–8RM was classified as high intensity; the programme reached 80–85% 1RM and 6–8 repetitions in the later phase. Under the progression rule, classification was based on the highest target intensity. | High-intensity aerobic training plus high-intensity resistance training |
